# Supplementary material for: CHD8 interacts with BCL11A to induce oncogenic transcription in triple negative breast cancer
Source: EMBO J. 2025 May 6;44(12):3448–67. doi: 10.1038/s44318-025-00447-8 (PMC12170886; doi:10.1038/s44318-025-00447-8)
Supplement: Supplementary file 7 — Appendix [file 44318_2025_447_MOESM7_ESM.pdf]

CHD8 interacts with BCL11A to induce oncogenic transcription in triple negative breast cancer

Table of contents

Appendix Figure S1 - Investigation of CHD8 as a novel mediator of TNBC	2
Appendix Figure S2 - Analysis of bulk RNAseq data	4
Appendix Figure S3 - Geneset enrichment analysis of quadrant A and C genes using gProfiler	6
Appendix Figure S4 - Analysis of ChIPseq data	7
Appendix Figure S5 - Analysis of genomic binding for directly regulated genes	9
Appendix Figure S6 - Truncated protein variants used for interface mapping	10
Appendix Figure S7 - SDS-PAGE and Western Blot of full-length BCL11B used for counter-screening of fragment hits	11
Appendix Figure S8 - Western Blot comparing levels of BCL11A and CHD8	12
Appendix Figure S9 Representative brightfield images comparing effect of fragments on colony size	13

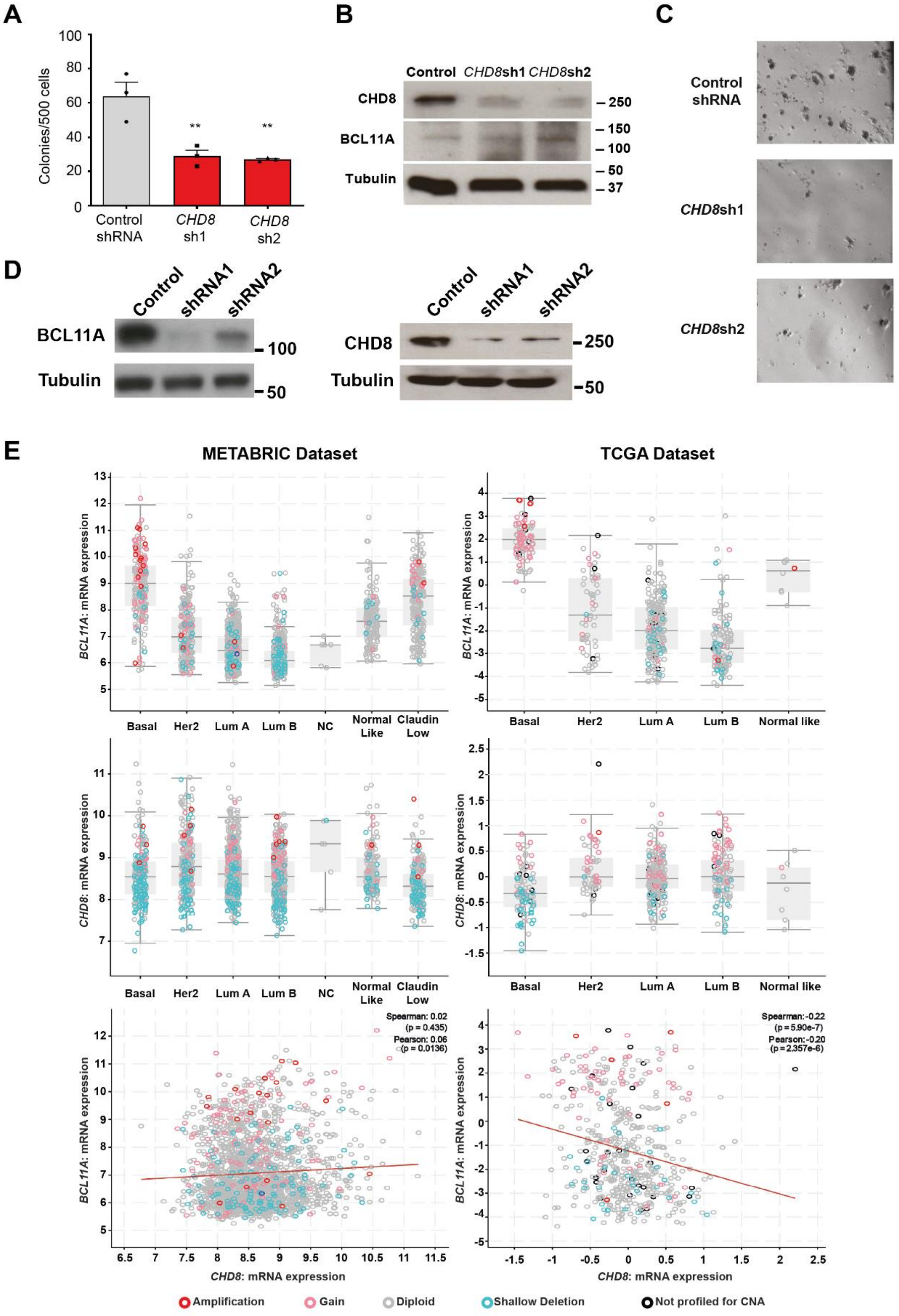
A
Colonies/500 cells
Control shRNA
CHD8 sh1
CHD8 sh2
**
B
Control
CHD8sh1
CHD8sh2
CHD8
BCL11A
Tubulin
250
150
100
50
37
C
Control shRNA
CHD8sh1
CHD8sh2
D
Control
shRNA1
shRNA2
BCL11A
Tubulin
100
50
CHD8
250
E
METABRIC Dataset
TCGA Dataset
BCL11A: mRNA expression
CHD8: mRNA expression
Basal
Her2
Lum A
Lum B
NC
Normal Like
Claudin Low
Normal like
Spearman: 0.02
(p = 0.435)
Pearson: 0.06
(p = 0.0136)
Spearman: -0.22
(p = 5.90e-7)
Pearson: -0.20
(p = 2.357e-6)
Amplification
Gain
Diploid
Shallow Deletion
Not profiled for CNA

Appendix Figure S1. Investigation of CHD8 as a novel mediator of TNBC

(A) shRNA-mediated knockdown of CHD8 in human TNBC MDA-MB-231 cells leads to a reduction in tumour growth in 3D colony assays, demonstrateing that CHD8 also plays a role in human TNBC cells. An ordinary one-way ANOVA with a Dunnett multiple comparison correction was used for statistical analysis of this plot. Means of each knockdown condition were compared to the mean of the control condition. ** = $p<0.01$. (B) Western Blot of shRNA-mediated CHD8 knockdown in MDA-MB-231 cells. (C) Representative example of raw images used to determine colony counts for panel A. (D) Cell lines and proteins used for Multi-OMICS and SPR analysis. Western Blots showing knockdown of BCL11A and CHD8 in 4T1 cells. 4T1 cells were transfected with BCL11A-targeting shRNA (left panel) or CHD8-targeting shRNA (right panel). Knockdown cells were submitted for multi-OMICS analysis. These panels are also shown in Fig. 1B&E. (B) mRNA expression of BCL11A and CHD8 in both the METABRIC and TCGA datasets. No correlation is observed between BCL11A and CHD8 mRNA expression in these datasets.

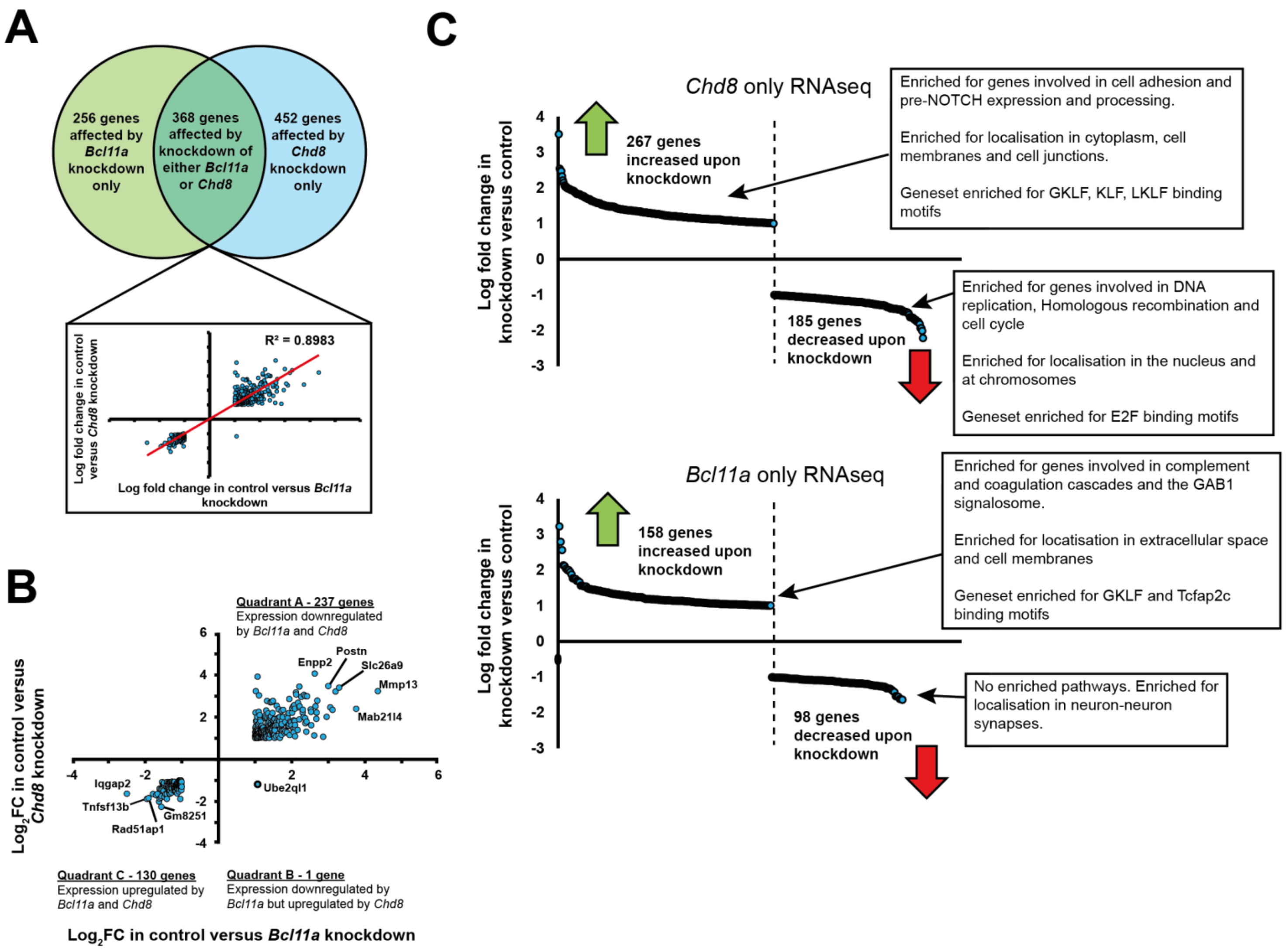
A
256 genes affected by Bcl11a knockdown only
368 genes affected by knockdown of either Bcl11a or Chd8
452 genes affected by Chd8 knockdown only
R² = 0.8983
Log fold change in control versus Chd8 knockdown
Log fold change in control versus Bcl11a knockdown
B
Quadrant A - 237 genes
Expression downregulated by Bcl11a and Chd8
Postn
Enpp2
Slc26a9
Mmp13
Mab21l4
Iqgap2
Tnfsf13b
Gm8251
Rad51ap1
Ube2ql1
Quadrant C - 130 genes
Expression upregulated by Bcl11a and Chd8
Quadrant B - 1 gene
Expression downregulated by Bcl11a but upregulated by Chd8
Log2FC in control versus Chd8 knockdown
Log2FC in control versus Bcl11a knockdown
C
Chd8 only RNAseq
Log fold change in knockdown versus control
267 genes increased upon knockdown
185 genes decreased upon knockdown
Enriched for genes involved in cell adhesion and pre-NOTCH expression and processing.
Enriched for localisation in cytoplasm, cell membranes and cell junctions.
Geneset enriched for GKLF, KLF, LKLF binding motifs
Enriched for genes involved in DNA replication, Homologous recombination and cell cycle
Enriched for localisation in the nucleus and at chromosomes
Geneset enriched for E2F binding motifs
Bcl11a only RNAseq
Log fold change in knockdown versus control
158 genes increased upon knockdown
98 genes decreased upon knockdown
Enriched for genes involved in complement and coagulation cascades and the GAB1 signalosome.
Enriched for locatisation in extracellular space and cell membranes
Geneset enriched for GKLF and Tcfap2c binding motifs
No enriched pathways. Enriched for localisation in neuron-neuron synapses.

Appendix Figure S2. Analysis of bulk RNAseq data

(A) Bulk RNAseq identified a number of gene targets affected only by Bcl11a or Chd8 knockdown, as well as a large subset of shared gene targets. Comparison of log fold change (logFC) values for the shared gene targets shows a high degree of similarity and high correlation (R2 = 0.8983) between Bcl11a and Chd8 knockdown, suggesting overlapping regulatory roles of these proteins for these gene targets. (B) Annotation of genes from the cross-plot in panel A with the largest LogFC changes, and annotation of quadrants A, B and C. In quadrant A, knockdown of either Bcl11a or Chd8 leads to an increase in expression (positive logFC), such that under normal circumstances this gene set is downregulated by these proteins. Conversely, in quadrant C, knockdown results in reduced expression (negative logFC), such that this gene set is normally upregulated by BCL11A and CHD8. (C) Gene set enrichment analysis of gene targets affected only by Bcl11a or Chd8 knockdown using gProfiler. Each point in the plot represents a gene within the dataset.

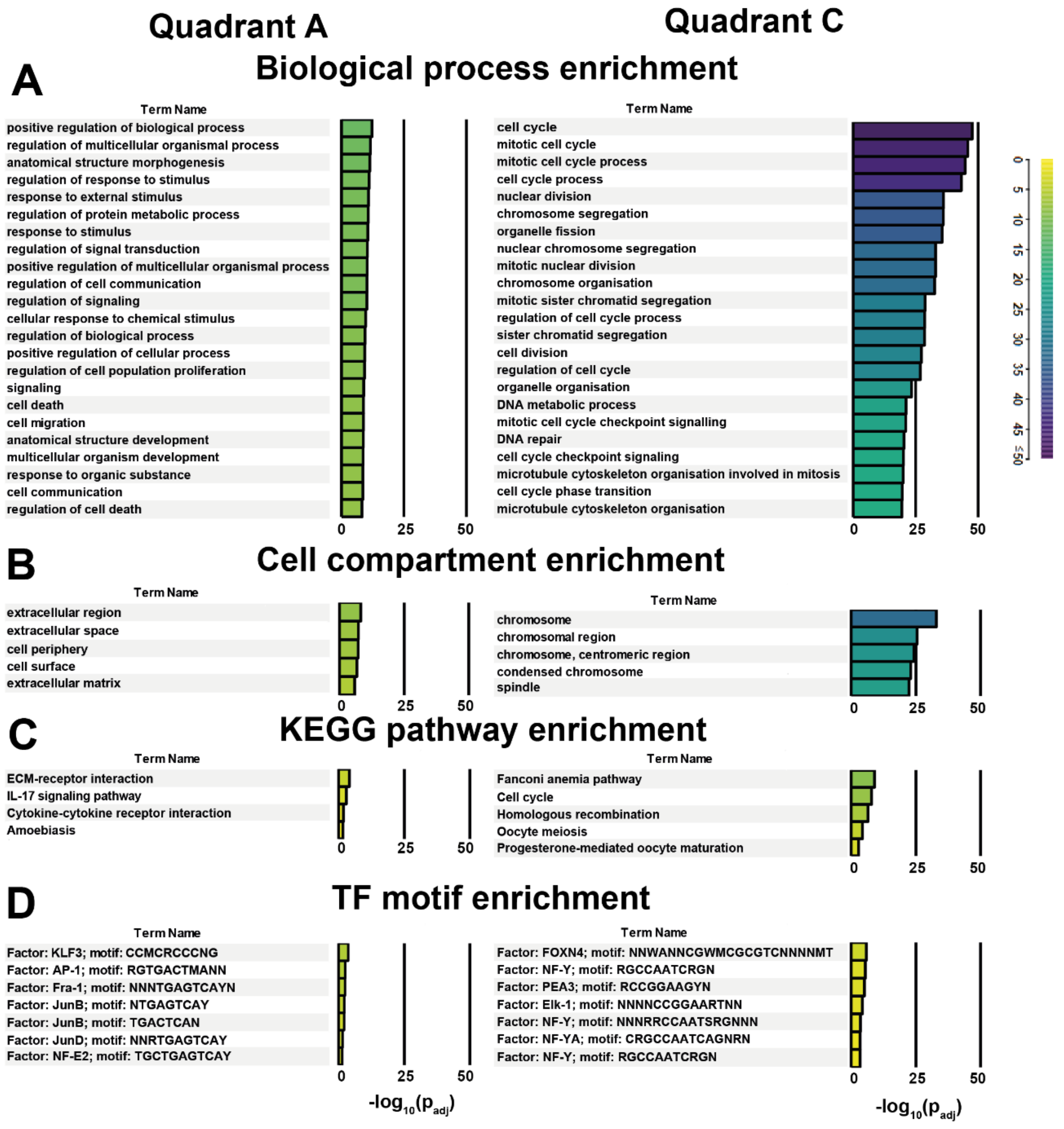

Appendix Figure S3. Geneset enrichment analysis of quadrant A and C genes using gprofiler. Genes identified in quadrants A and C in Appendix Figure S2 were submitted for GSEA using gProfiler, which identified the following: Biological process enrichment terms (A), cell compartment enrichment terms (B), KEGG pathway enrichment terms (C) and transcription factor binding motif enrichment terms (D).

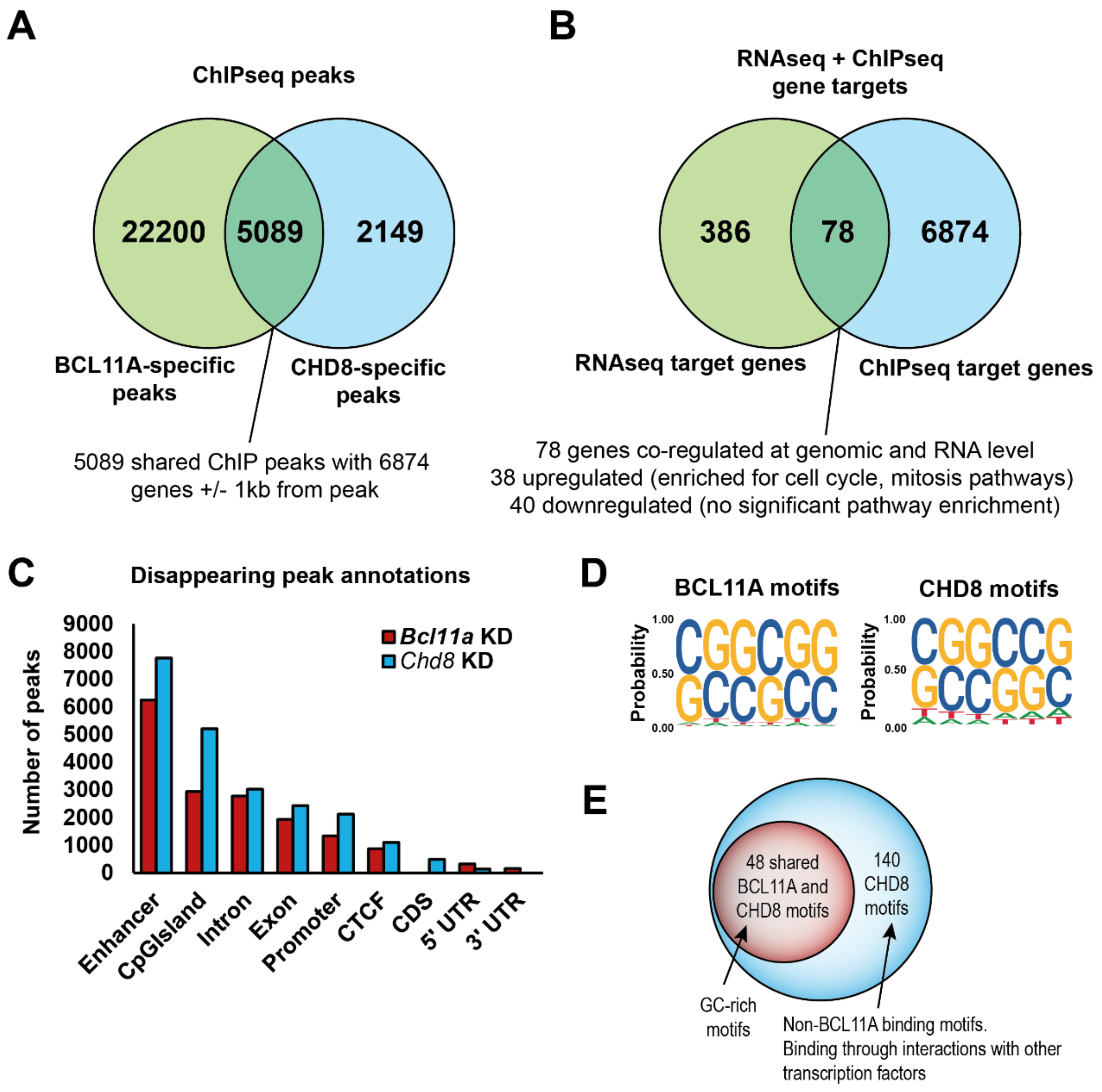

Appendix Figure S4. Analysis of ChIPseq data

(A) BCL11A and CHD8 specific peaks were characterised by identifying genomic binding peaks that were reduced or "disappeared" in knockdown samples versus control. This identified a number of BCL11A and CHD8-specific peaks as well as a subset of directly overlapping peaks. 6874 genes were identified in the vicinity (+/- 1kb) of this shared peak set. (B) Intersection of the 6874 genes identified from panel A with the shared RNAseq target gene set identifies genes likely to be directly regulated by BCL11A and CHD8. This identified 78 genes that were genomically bound by BCL11A and CHD8 and were also altered at the RNA level upon knockdown. (C) Assignment of genomic feature annotations to the disappearing peaks identified in panel A. All disappearing peaks were considered, whether protein-specific or shared. This showed that BCL11A and CHD8

have similar genomic binding biases, with most binding observed in enhancer, CpG island, introns, exons and promoter regions. (D) Motif enrichment analysis of the shared disappearing peak subset. This was performed to identify sequences that are likely binding sites of BCL11A and CHD8, which identified a strong bias for binding GC-rich sequences. (E) Motif enrichment analysis of all disappearing peaks. This showed a complete overlap of BCL11A motifs with CHD8 motifs, but identified a large number of additional motifs that are enriched for CHD8 binding, suggesting that BCL11A is completely dependent on CHD8 for genomic binding but that CHD8 is only partly dependent on BCL11A, suggesting a number of additional CHD8-binding partners present that facilitate wide ranging genomic binding.

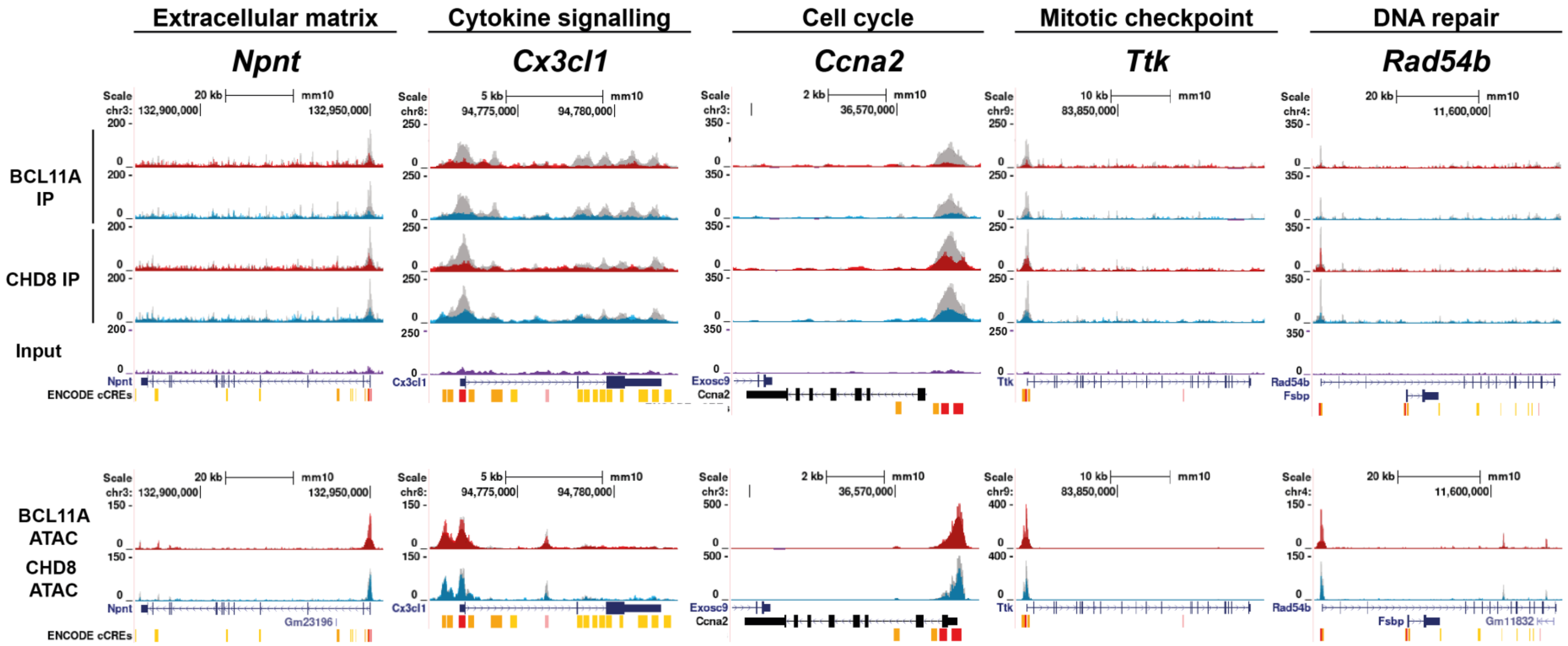

Appendix Figure S5. Analysis of genomic binding for directly regulated genes

Manual inspection of genes within the RNAseq-ChIPseq intersect show consistent reduction of genomic binding by ChIPseq (top panels) regardless of which interaction partner is knocked down. Genomic binding peaks are also heavily biased towards promoter regions. Manual inspection of ATACseq data (bottom panels) shows that knockdown of Chd8, but not Bcl11a, reduces chromatin accessibility at the promoter regions of shared gene targets.

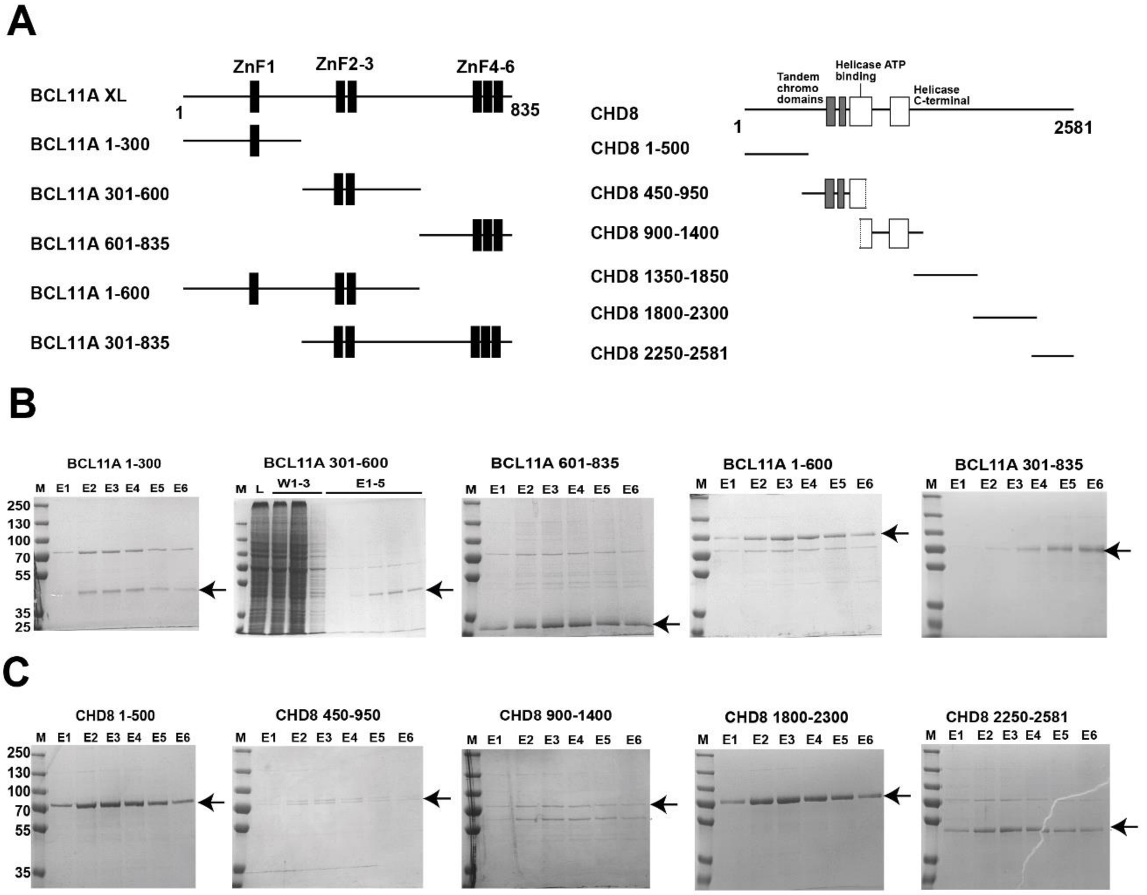

Appendix Figure S6. Truncated protein variants used for interface mapping

(A) Schematic of the truncated variants cloned and produced for mapping. Motifs and domains are indicated. (B and C) Data shown corresponds to the collection of consecutive elution fractions from StrepTactin XT 4-Flow resin. The majority of protein fragments are well purified with arrows depicting the protein of interest. In a number of cases, a contaminating band can be observed at ~70kDa. We were unable to express and purify CHD8 1350-1850 due to poor expression and solubility. M: Marker, L: Lysate, W: Wash fractions; E: Elution fractions

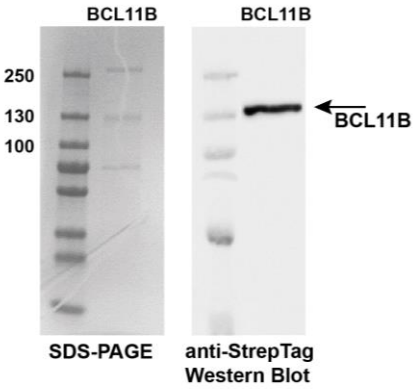

Appendix Figure S7 SDS-PAGE and Western Blot of full-length BCL11B used for counter-screening of fragment hits.

To investigate the specificity of the identified BCL11A binders, the homologous family member BCL11B was produced and purified in Expi293F cells. Purified protein was analysed by SDS-PAGE and Western Blotting, which demonstrated highly purified protein.

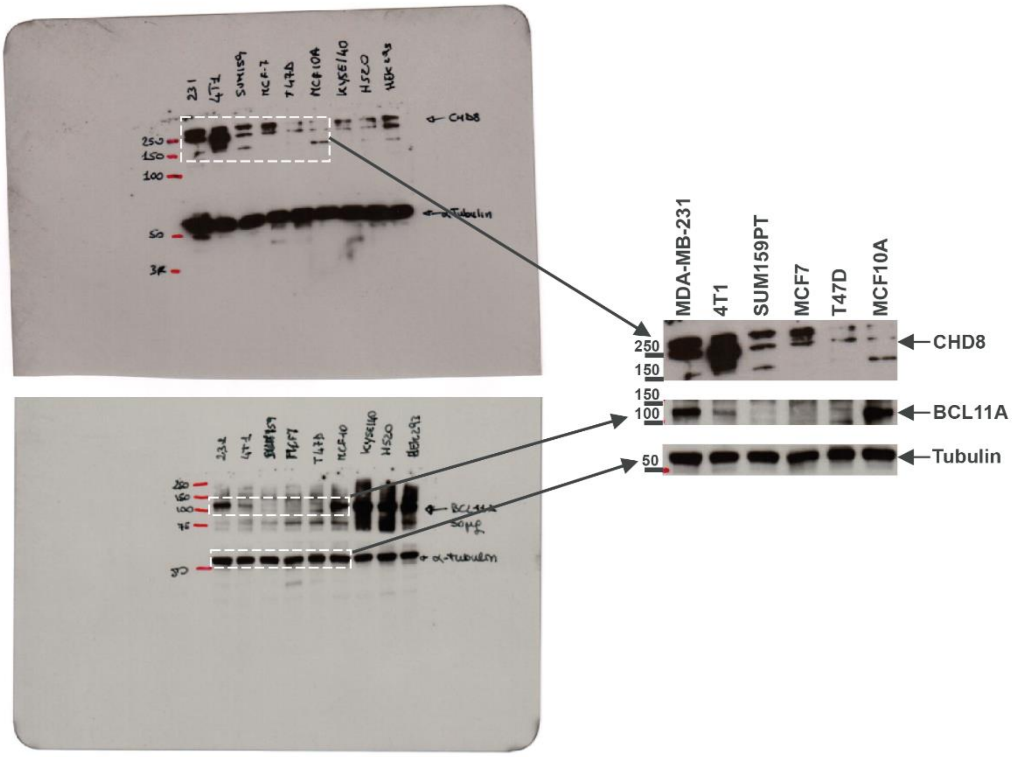

Appendix Figure S8 - Western Blot comparing levels of BCL11A and CHD8 expression across different mammary epithelial cell lines. The original Western Blot x-ray films (left panels) are included in a side-by-side comparison with the cropped figure (right panel)

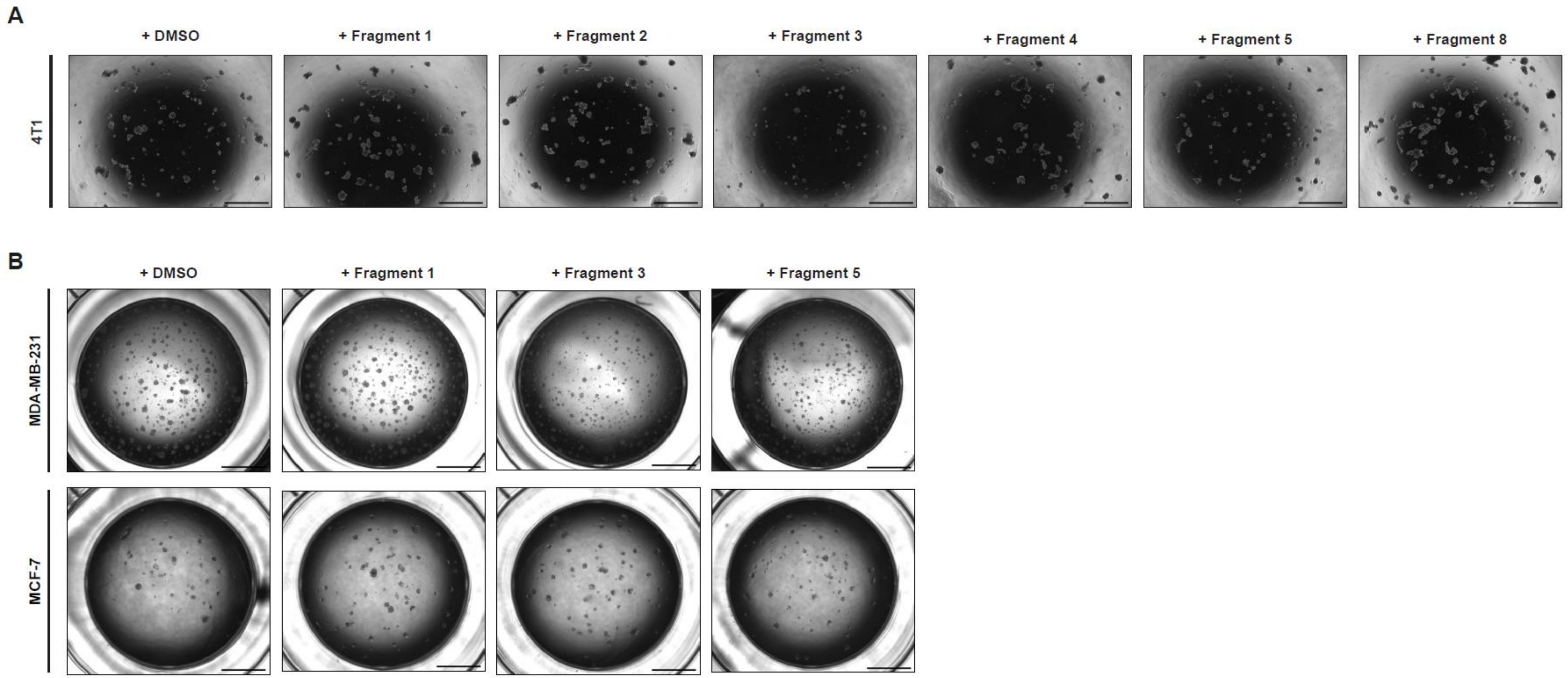

Appendix Figure S9 Representative brightfield images comparing effect of fragments on colony size.

3D colony assay images visualising the phenotypic changes in (A) 4T1 colony size following 6 days of treatment with all 6 binders at 200uM, (B) MDA-MB-231 and MCF7 colony size following 6 days of treatment with 3 selected binders at 200uM. All scale bars represents 2000um.
