# Supplementary figures and images for: CHD8 interacts with BCL11A to induce oncogenic transcription in triple negative breast cancer
Source: EMBO J. 2025 May 6;44(12):3448–67. doi: 10.1038/s44318-025-00447-8 (PMC12170886; doi:10.1038/s44318-025-00447-8)

## Slide 1
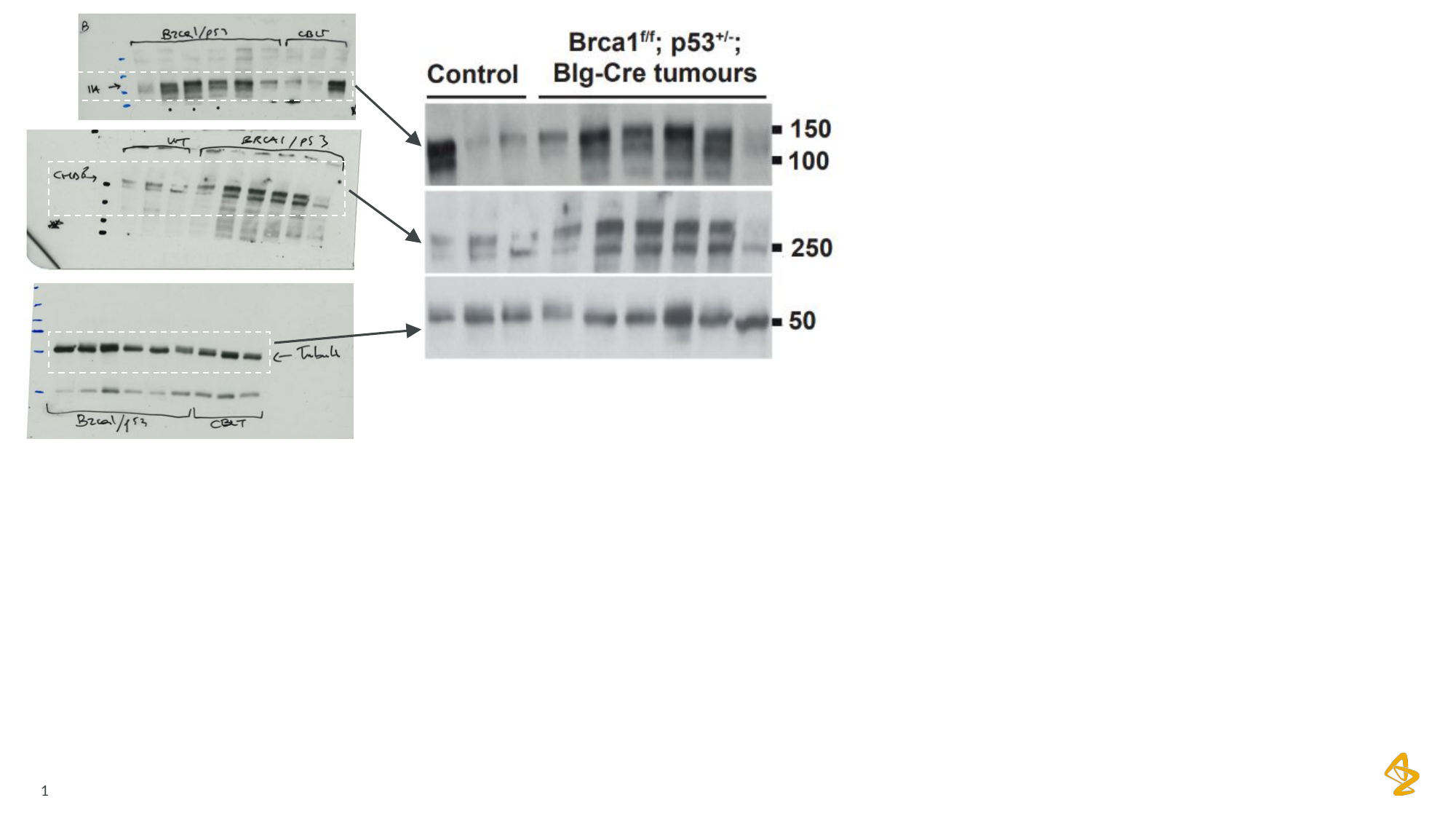

1

Supplement: Supplementary file 30 — Source data Fig. 1 [file 44318_2025_447_MOESM30_ESM.zip › Figure 1/Fig 1d/Fig 1d comparison.pptx]

## Slide 1
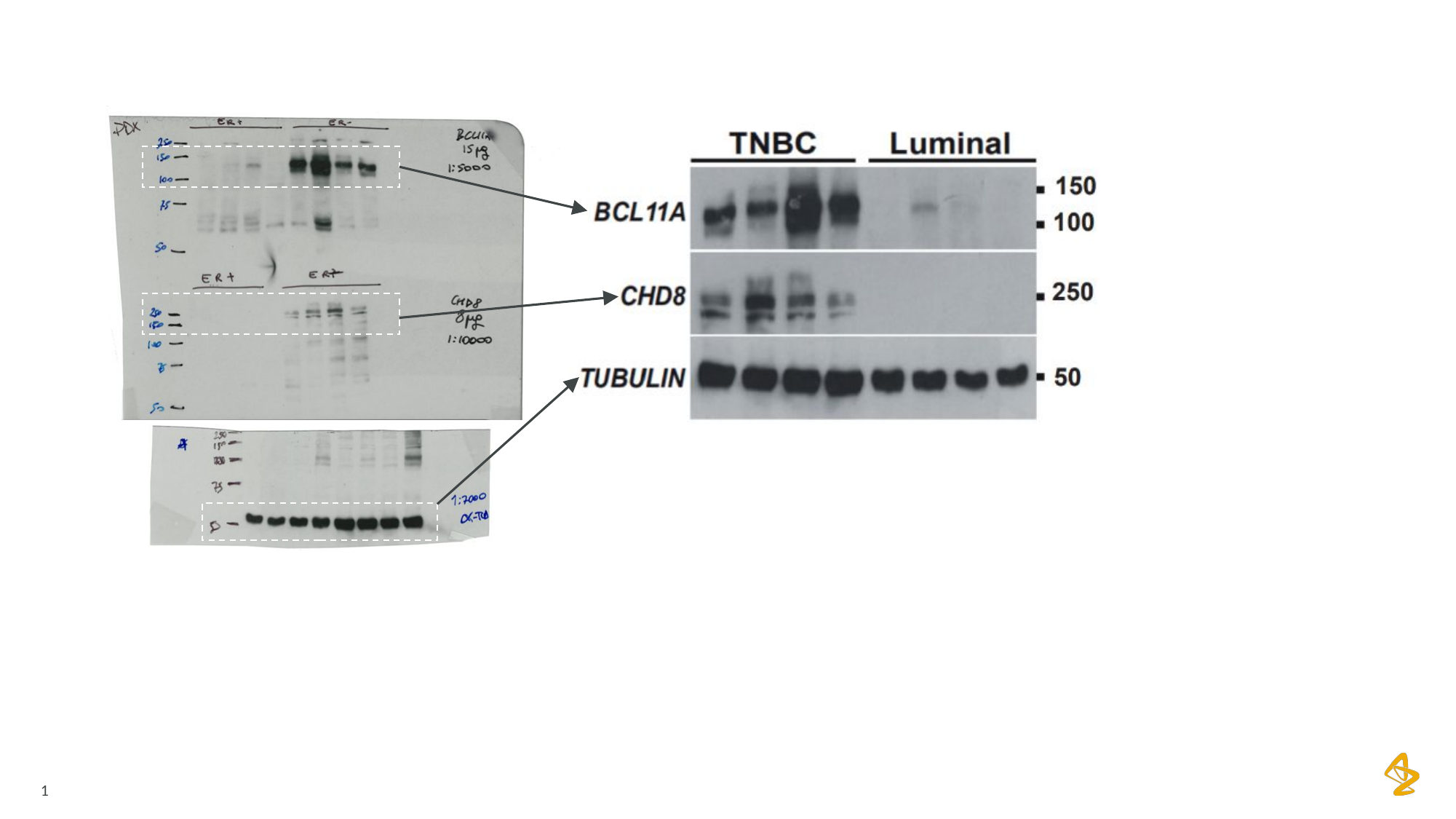

1

Supplement: Supplementary file 30 — Source data Fig. 1 [file 44318_2025_447_MOESM30_ESM.zip › Figure 1/Fig 1c/Fig 1c comparison.pptx]

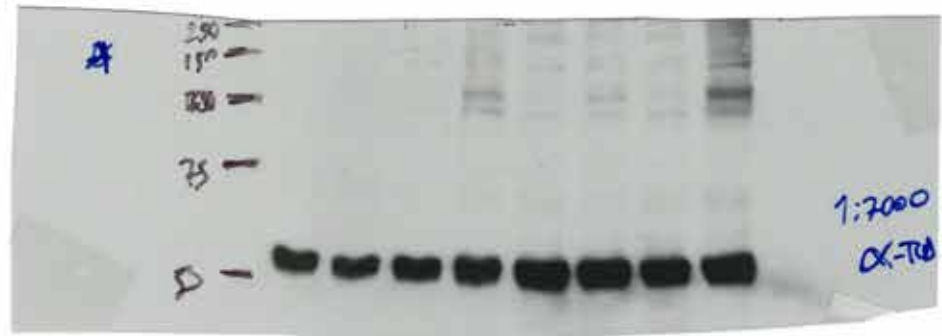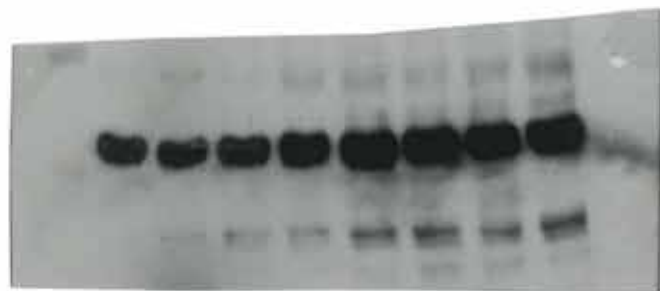

Supplement: Supplementary file 30 — Source data Fig. 1 [file 44318_2025_447_MOESM30_ESM.zip › Figure 1/Fig 1c/Fig 1c-2.pdf]

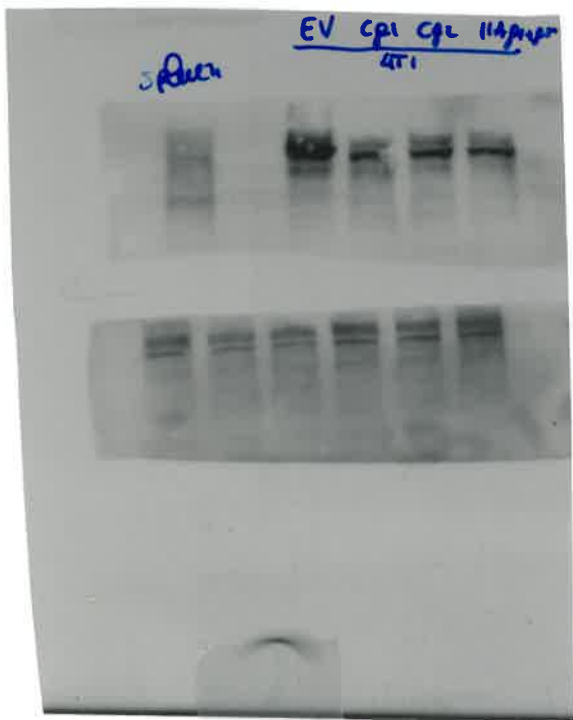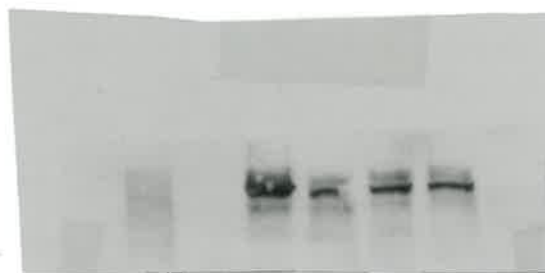

WB  
 p-CHD3

Stripped membrane

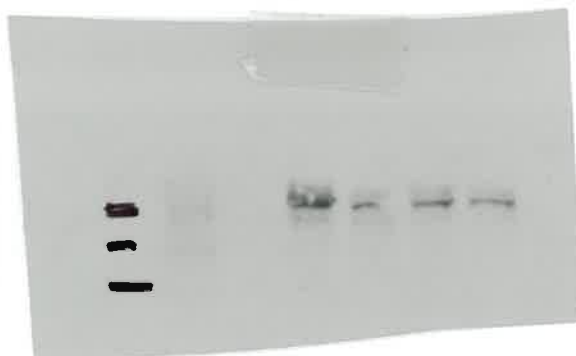

WB  
 p-CHD3

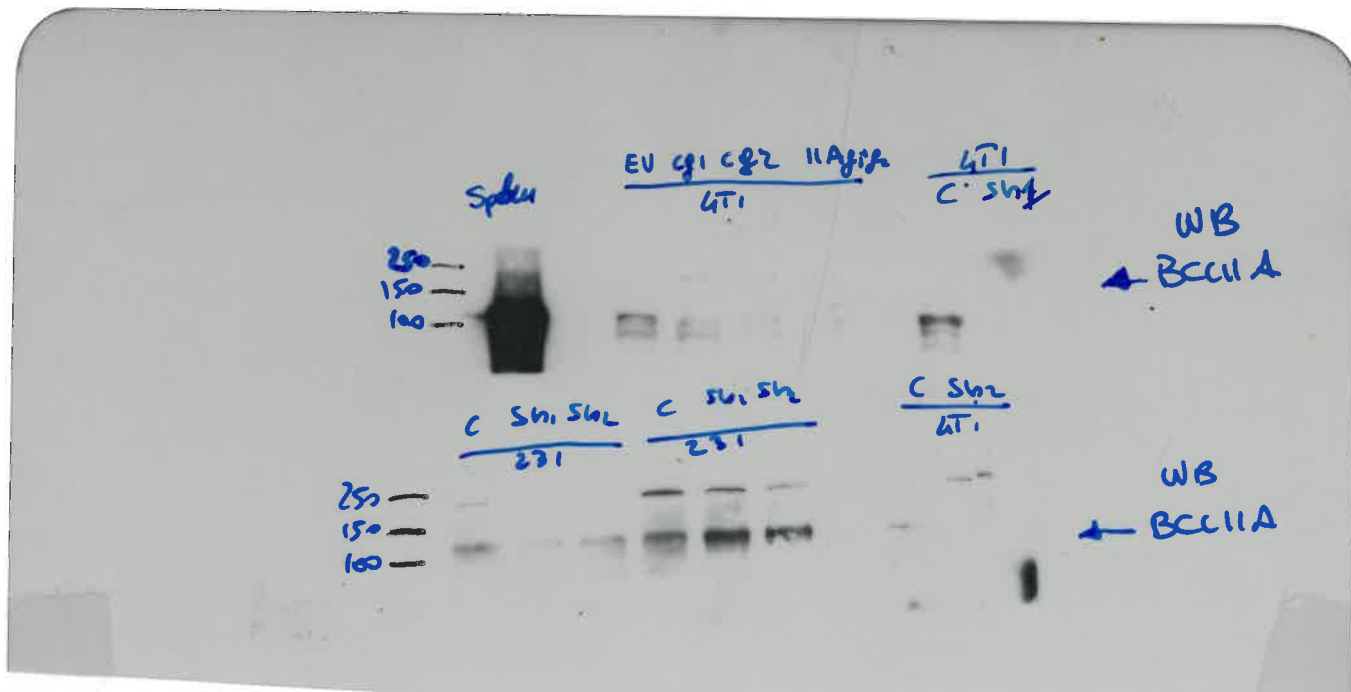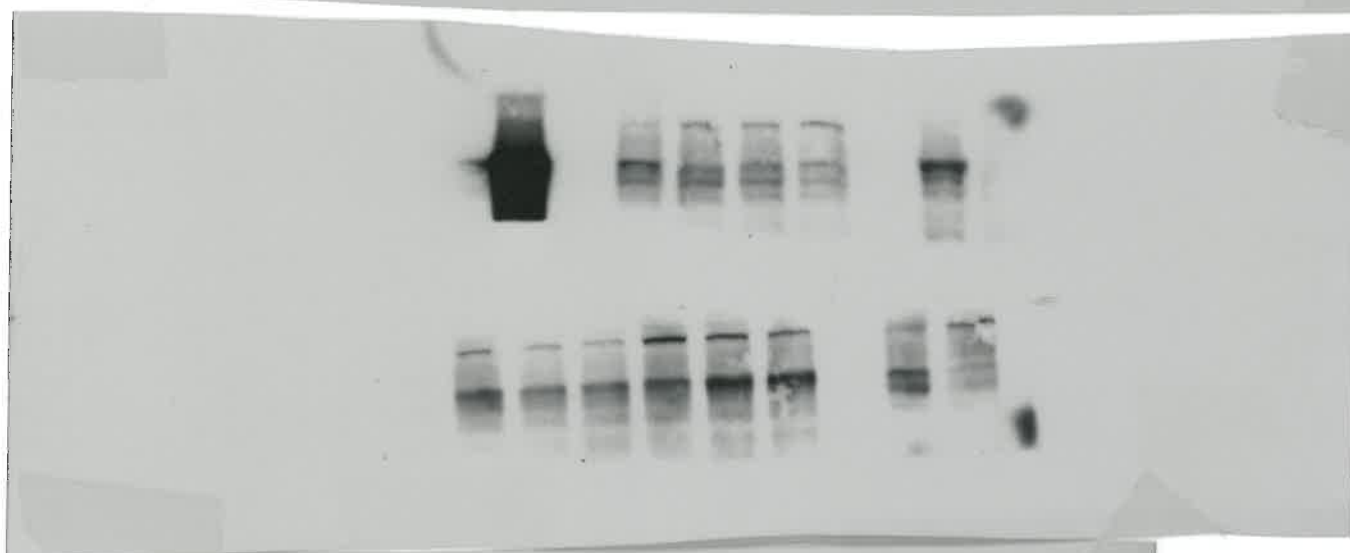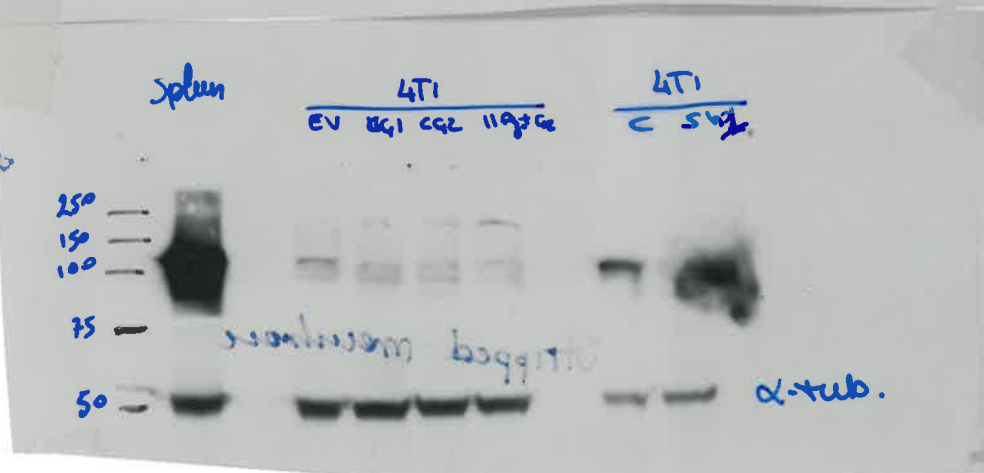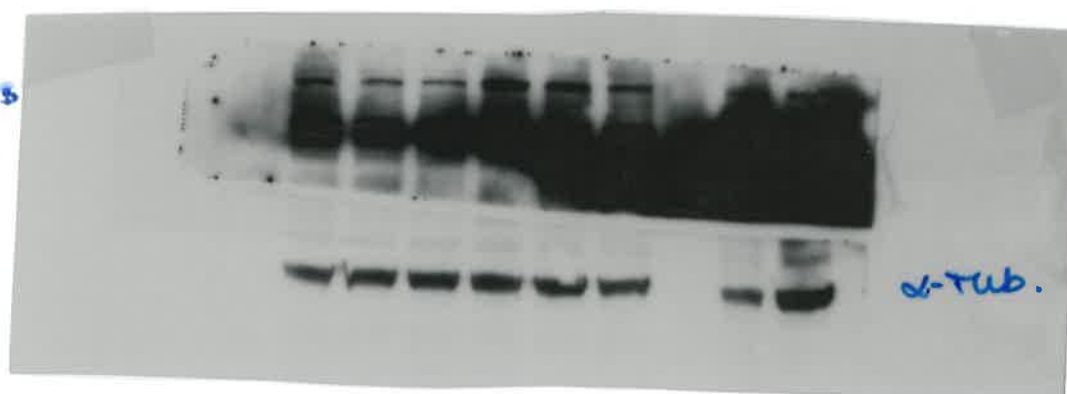

Supplement: Supplementary file 30 — Source data Fig. 1 [file 44318_2025_447_MOESM30_ESM.zip › Figure 1/Fig 1b/CHD8 gRNA blots.pdf]

## Slide 1
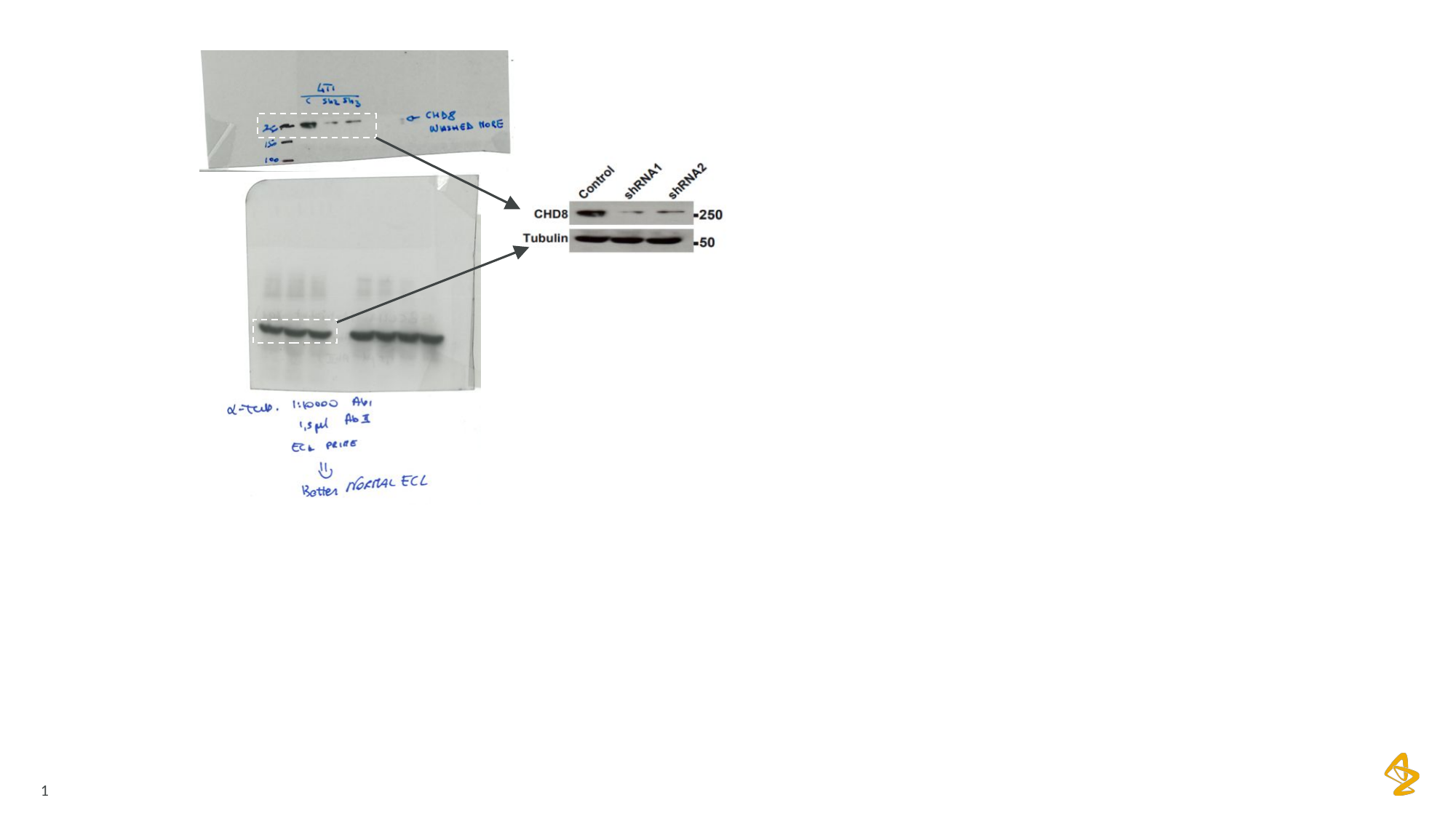

1

Supplement: Supplementary file 30 — Source data Fig. 1 [file 44318_2025_447_MOESM30_ESM.zip › Figure 1/Fig 1b/Fig 1b Comparison.pptx]

## Slide 1
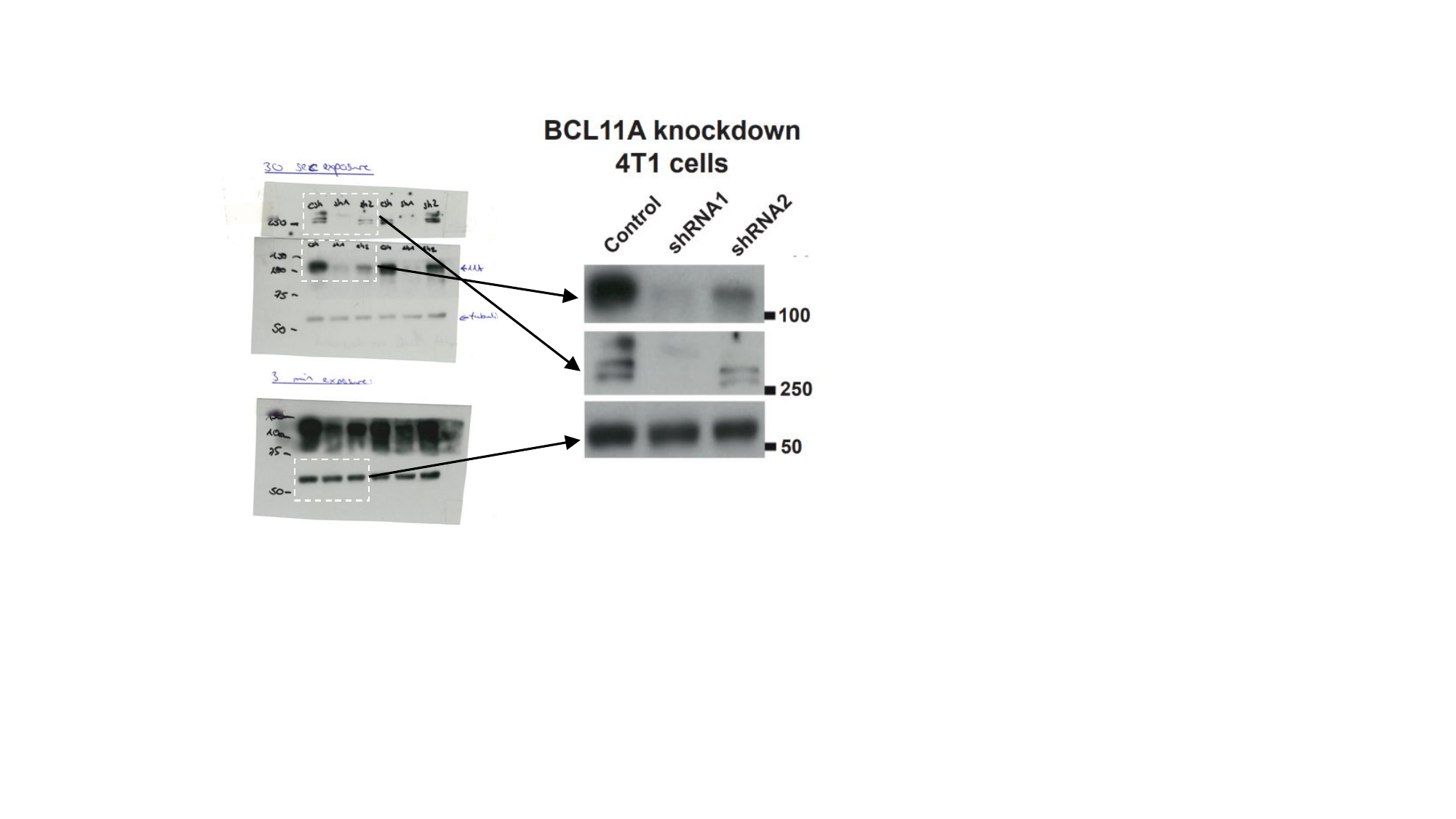

Supplement: Supplementary file 30 — Source data Fig. 1 [file 44318_2025_447_MOESM30_ESM.zip › Figure 1/Fig 1e/Fig 1e comparison.pptx]

30 sec exposure

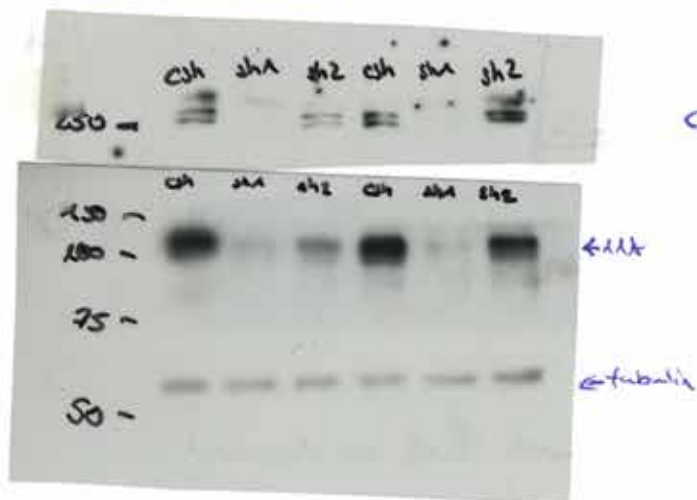

1 min exposure

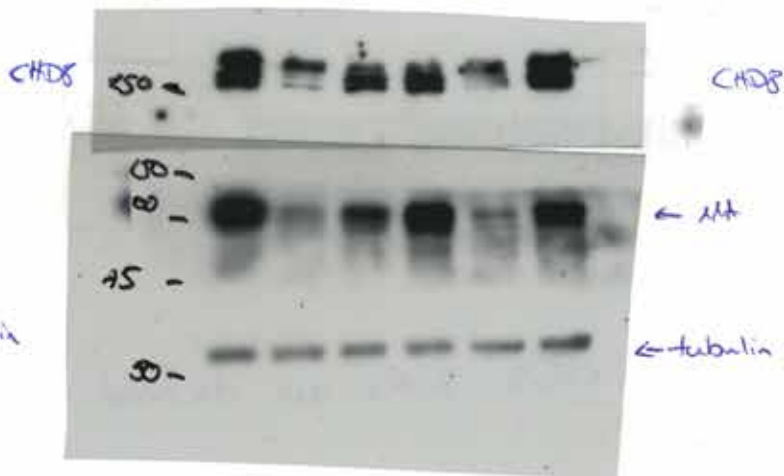

3 min exposure

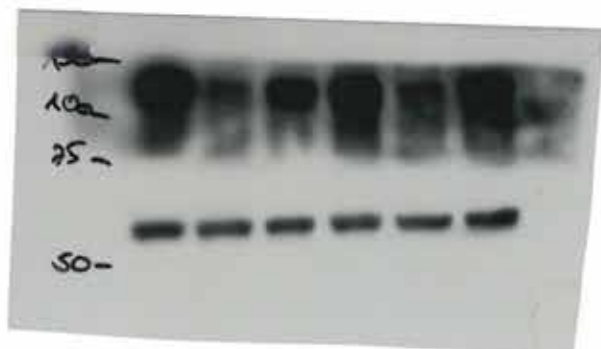

Supplement: Supplementary file 30 — Source data Fig. 1 [file 44318_2025_447_MOESM30_ESM.zip › Figure 1/Fig 1e/Fig 1e.pdf]

## Slide 1
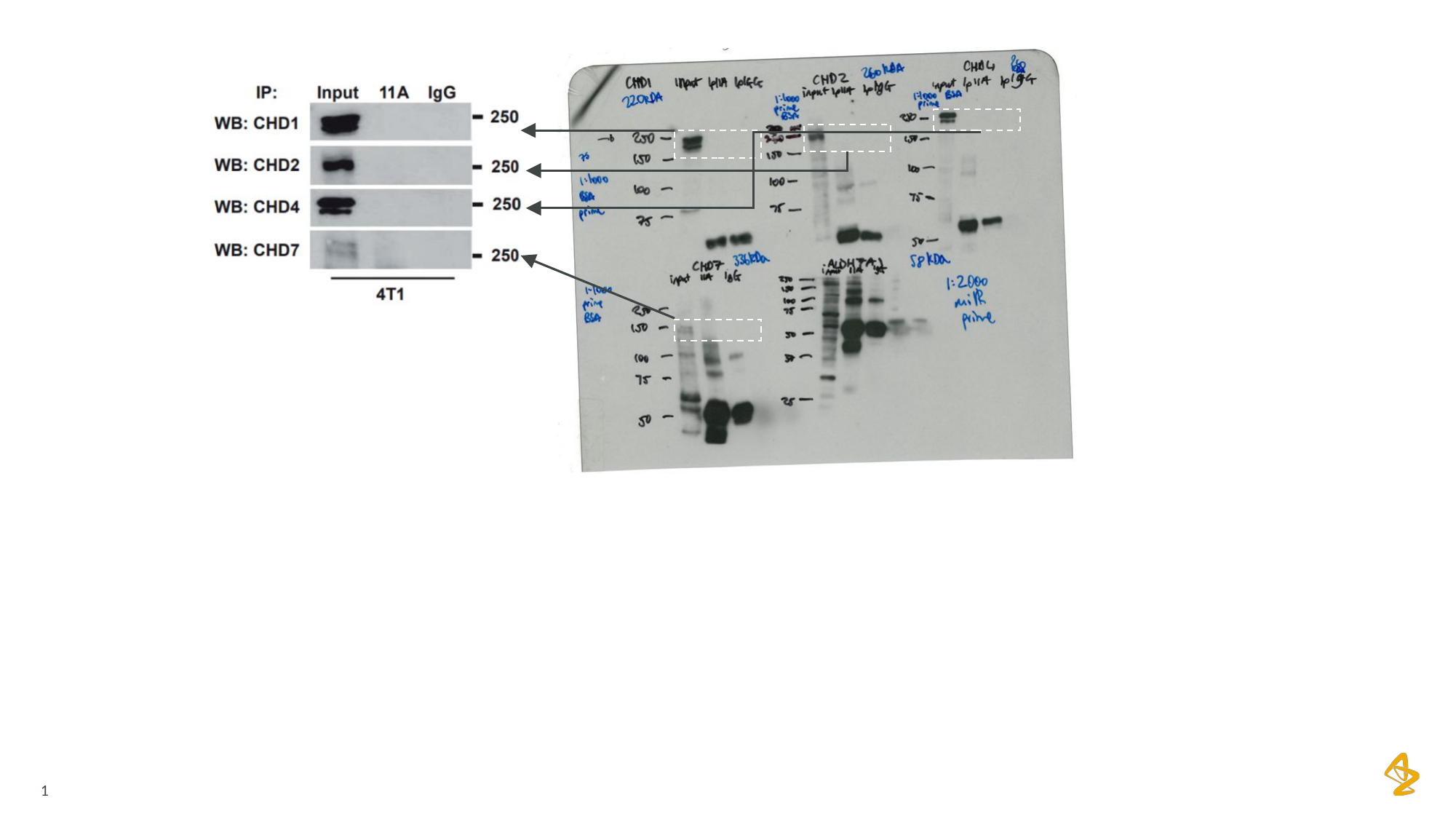

1

Supplement: Supplementary file 31 — Source data Fig. 3 [file 44318_2025_447_MOESM31_ESM.zip › Figure 3/Fig 3b/4T1 comparison Fig 3b.pptx]

## Slide 1
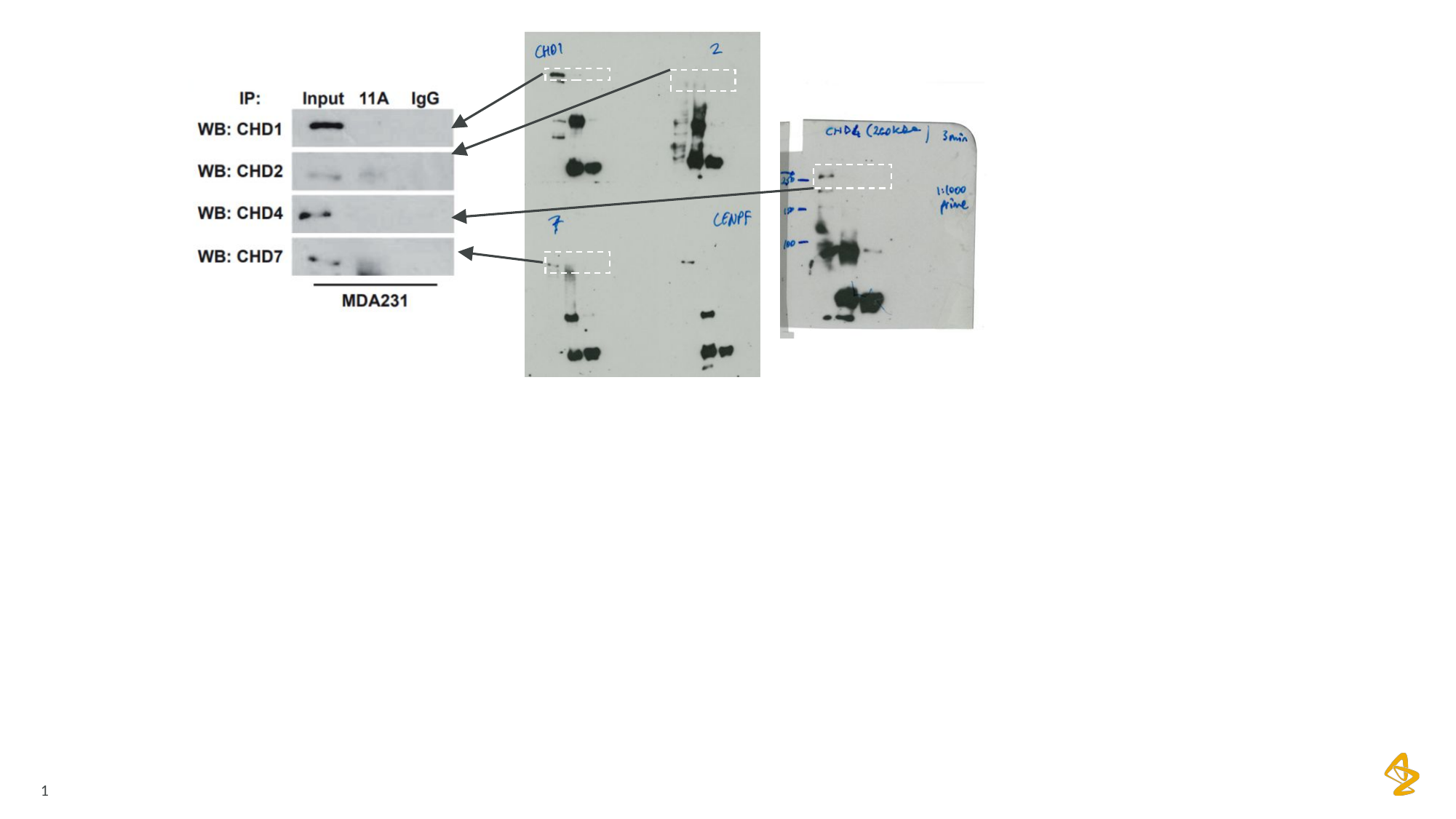

1

Supplement: Supplementary file 31 — Source data Fig. 3 [file 44318_2025_447_MOESM31_ESM.zip › Figure 3/Fig 3b/231 comparison Fig 3b.pptx]

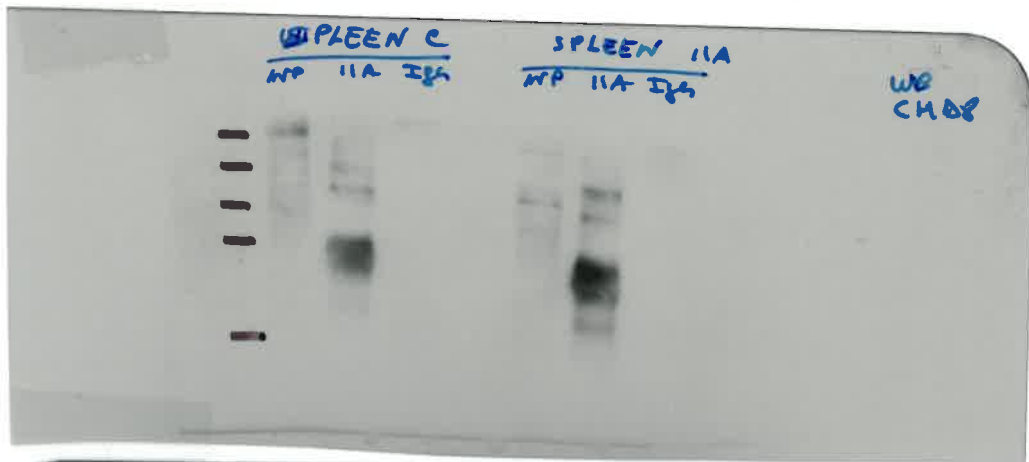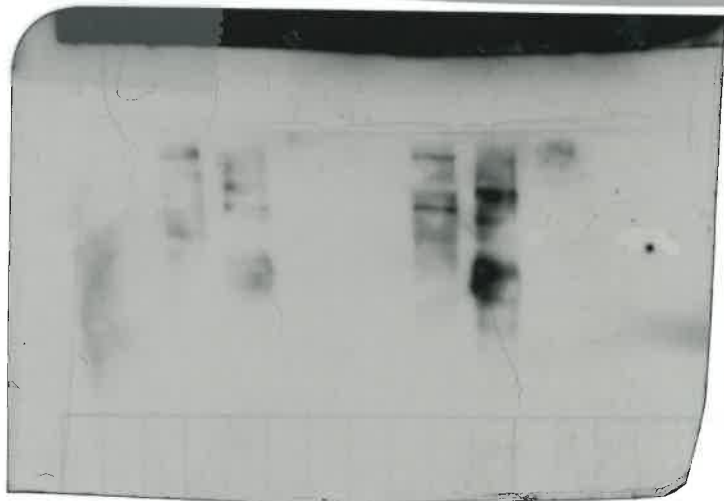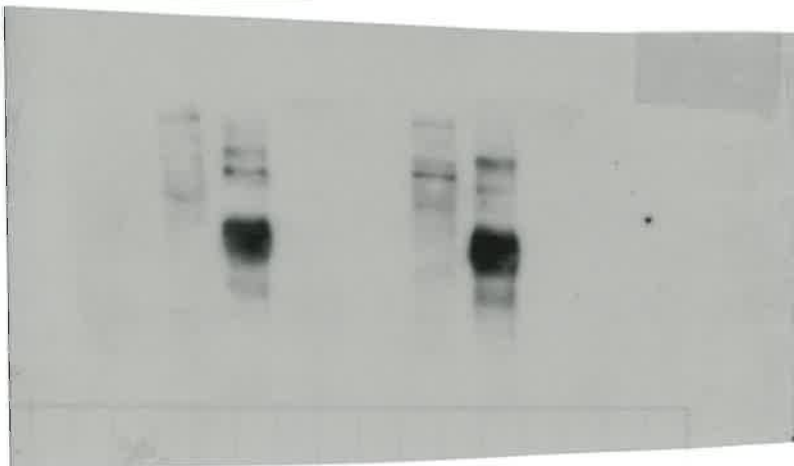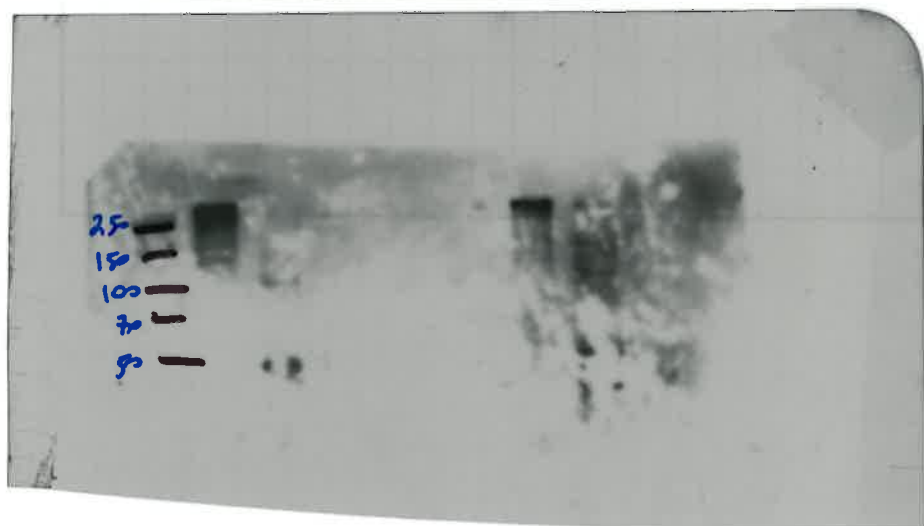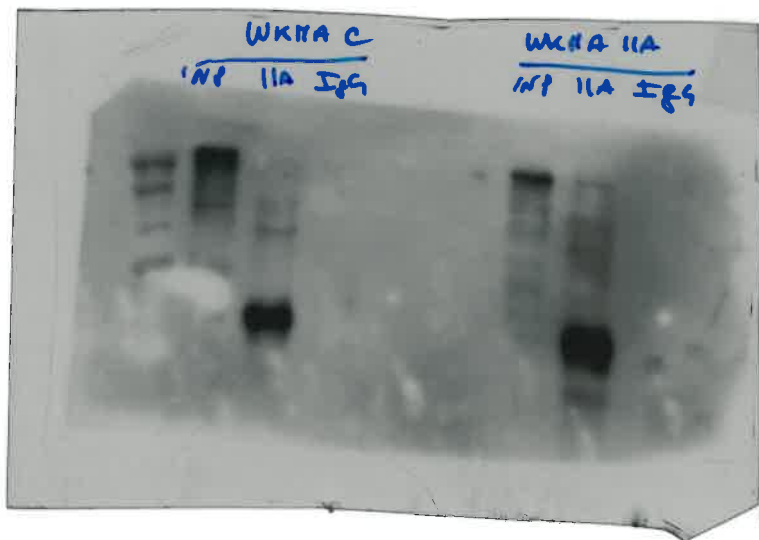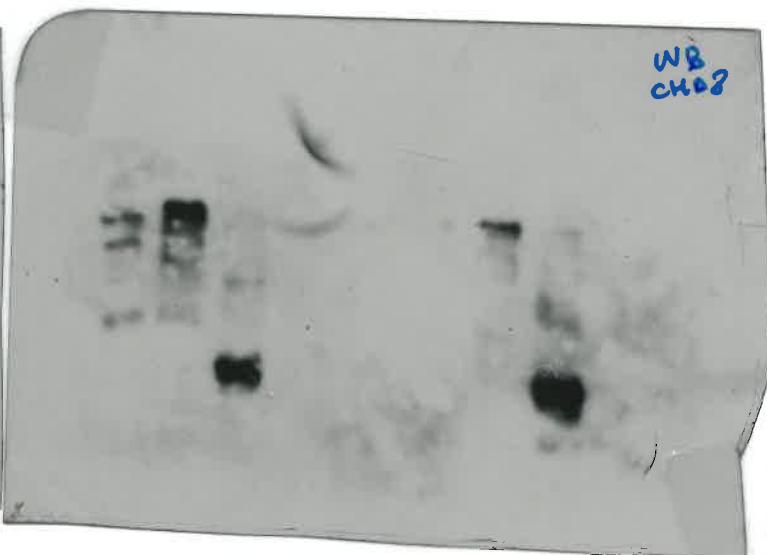

Supplement: Supplementary file 31 — Source data Fig. 3 [file 44318_2025_447_MOESM31_ESM.zip › Figure 3/Fig 3a/Spleen CHD8 blot.pdf]

## Slide 1
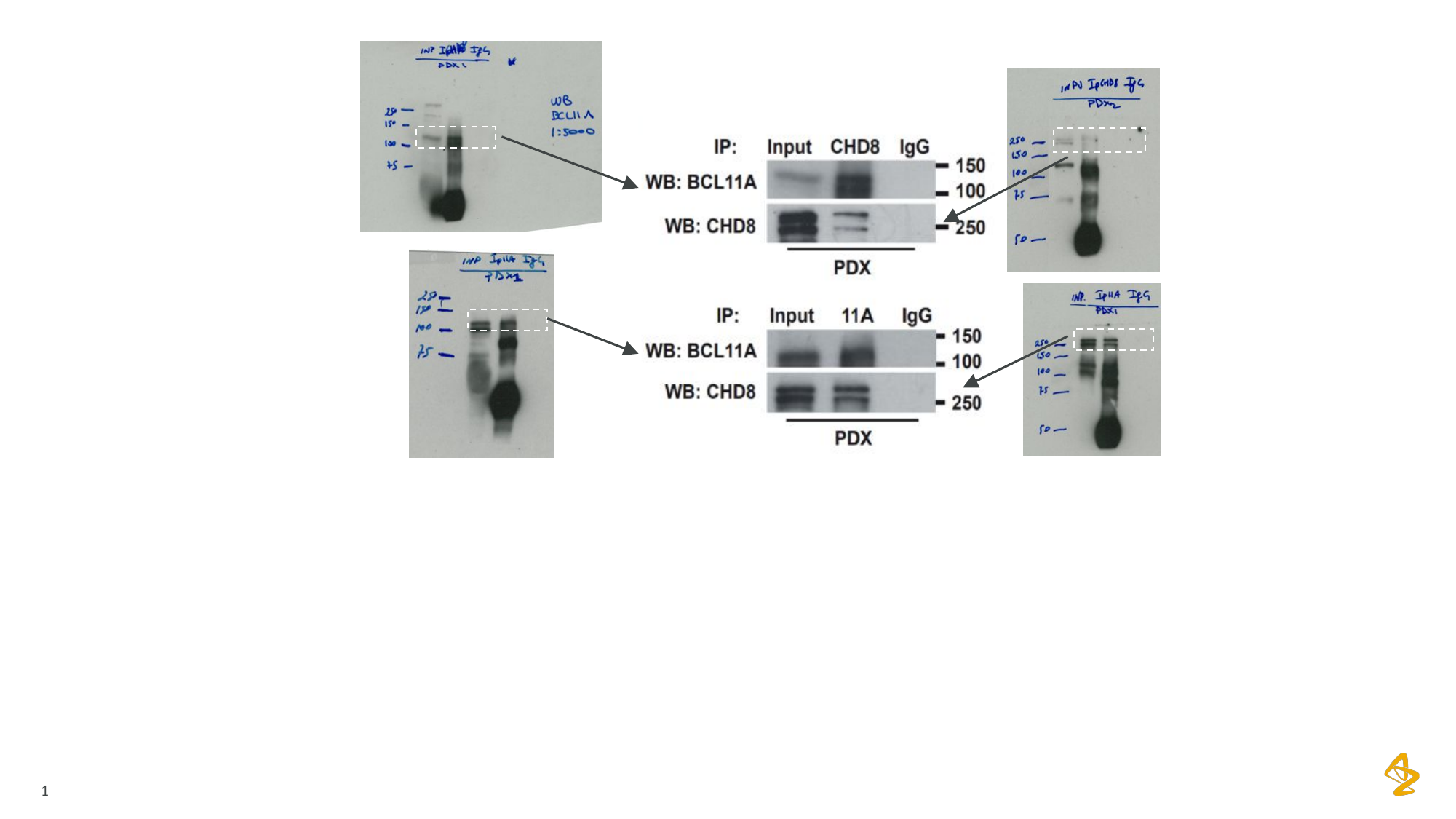

1

Supplement: Supplementary file 31 — Source data Fig. 3 [file 44318_2025_447_MOESM31_ESM.zip › Figure 3/Fig 3a/PDX comparison.pptx]

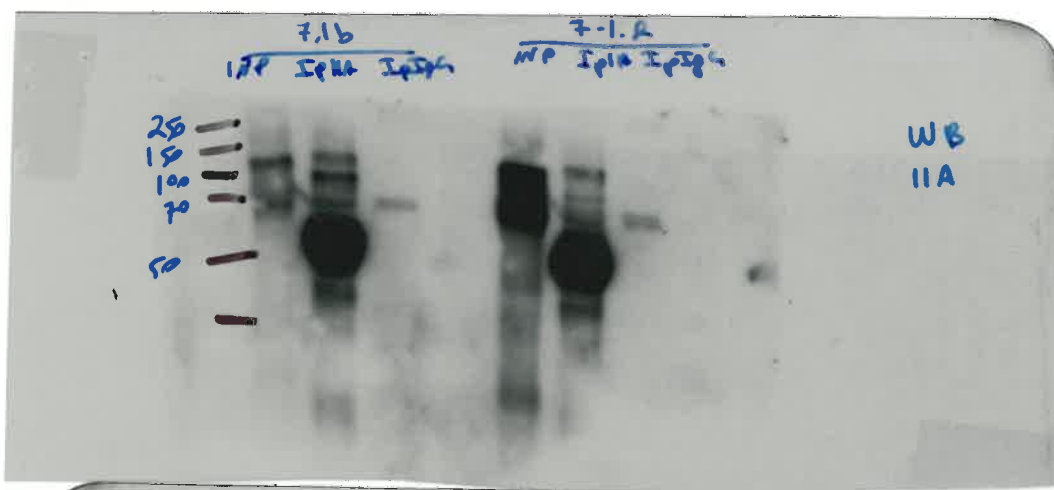

♂ spleen

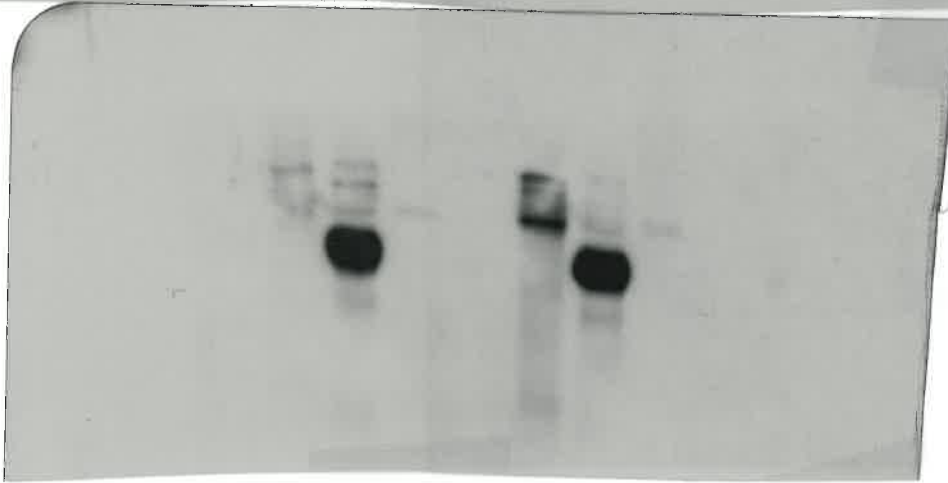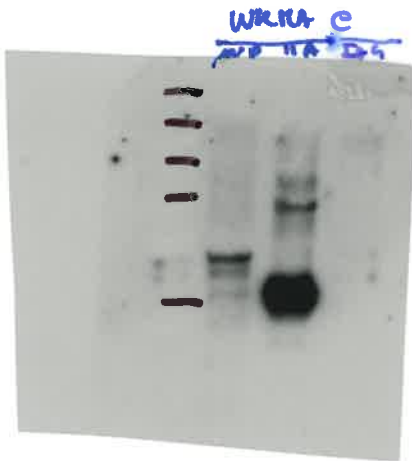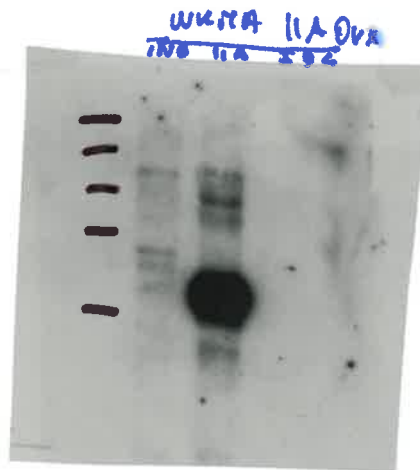

usage 6

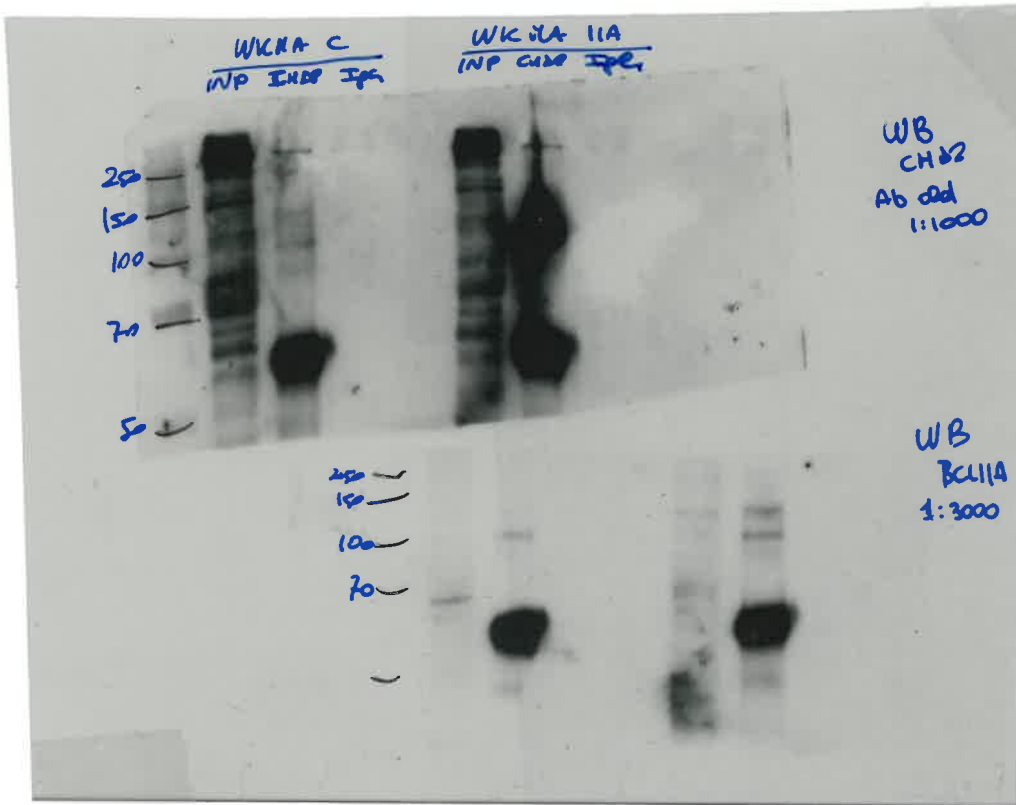

WKNA C  
WKNA 11A

WKNA C  
WKNA 11A

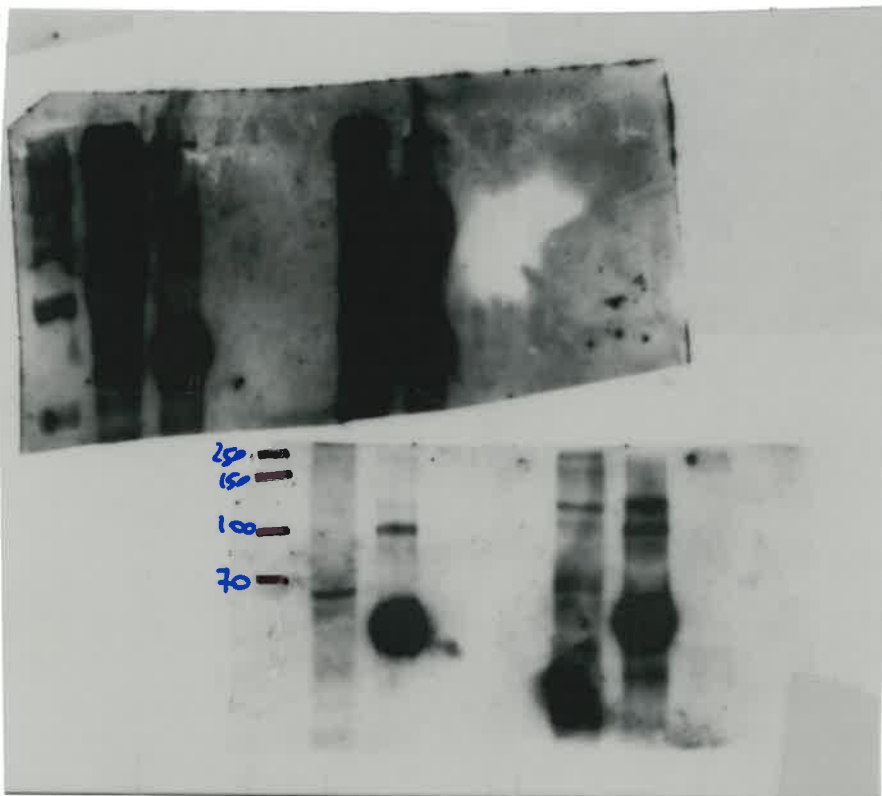

Supplement: Supplementary file 31 — Source data Fig. 3 [file 44318_2025_447_MOESM31_ESM.zip › Figure 3/Fig 3a/Spleen 11a blot.pdf]

## Slide 1
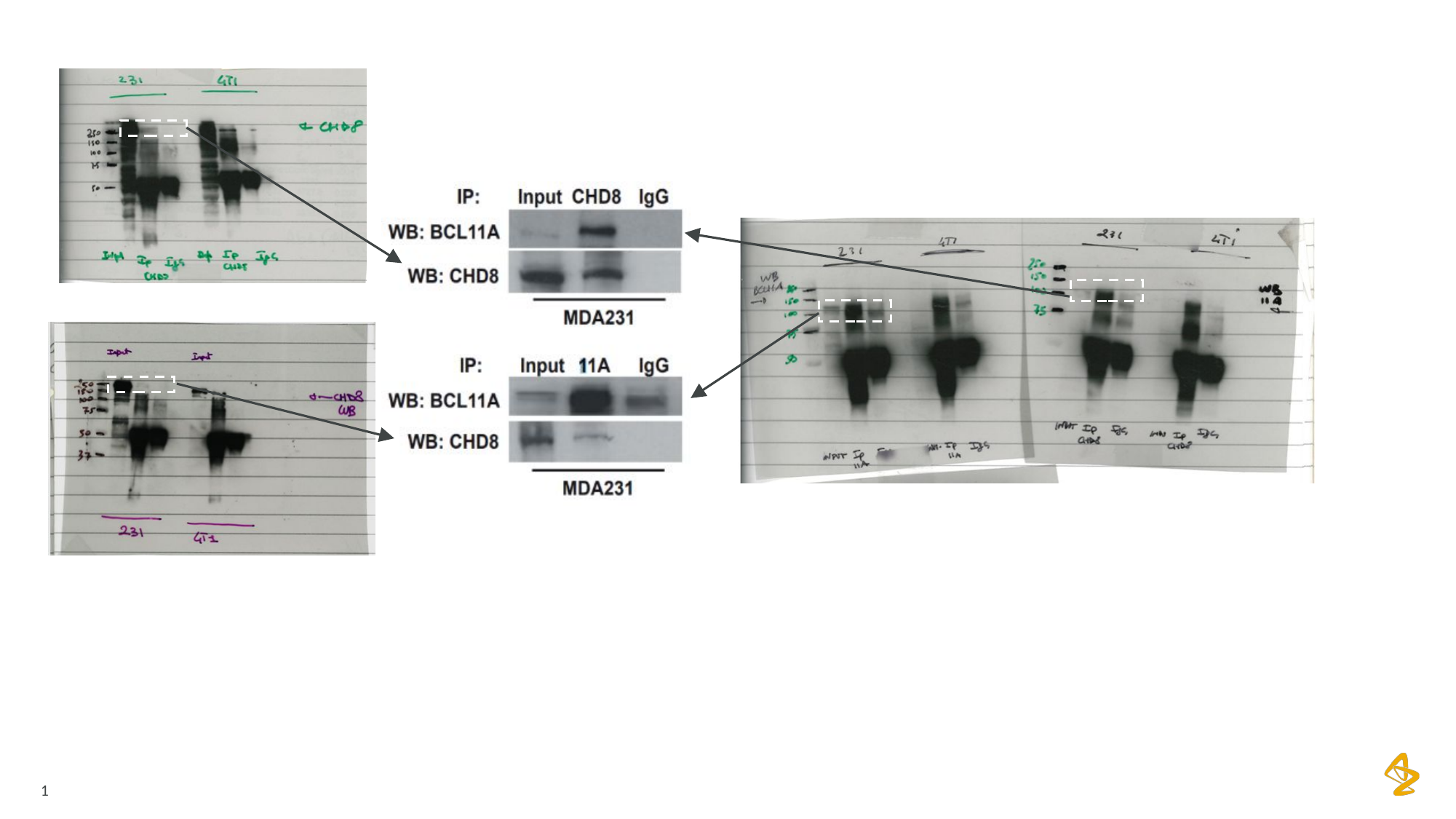

1

Supplement: Supplementary file 31 — Source data Fig. 3 [file 44318_2025_447_MOESM31_ESM.zip › Figure 3/Fig 3a/Fig 3a 231 comparison.pptx]

## Slide 1
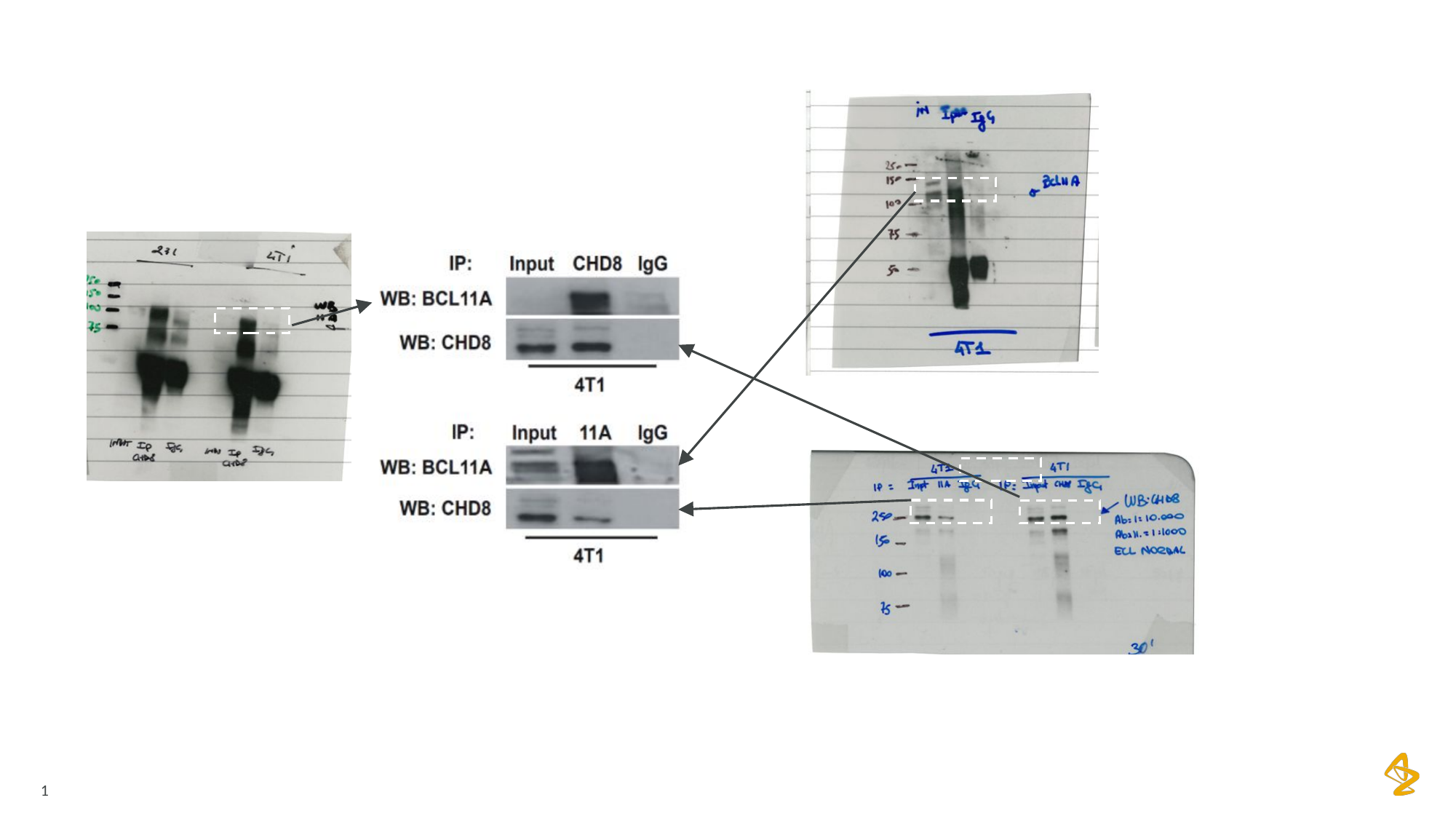

1

Supplement: Supplementary file 31 — Source data Fig. 3 [file 44318_2025_447_MOESM31_ESM.zip › Figure 3/Fig 3a/Fig 3a 4T1 comparison.pptx]

4T2  
IP: Input HA IgG

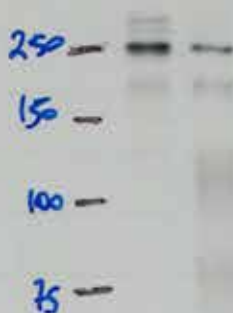

4T1  
IP: Input CHM IgG

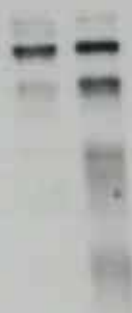

WB: CHM8  
Ab: 1:10,000  
Ab: 11: 1:1000  
ELL NORMAL

30'

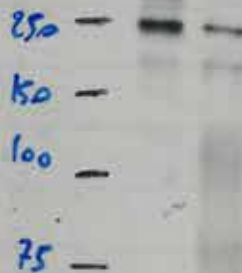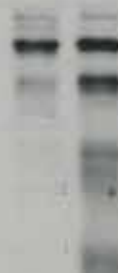

12 min

Supplement: Supplementary file 31 — Source data Fig. 3 [file 44318_2025_447_MOESM31_ESM.zip › Figure 3/Fig 3a/Fig 3a 4t1 11a IP chd8 blot and chd8 chd8.pdf]

100 IgG IgG  
+ DMSO

100 IgG IgG  
+ DMSO

250 —  
150 —  
100 —  
75 —

WB  
HA

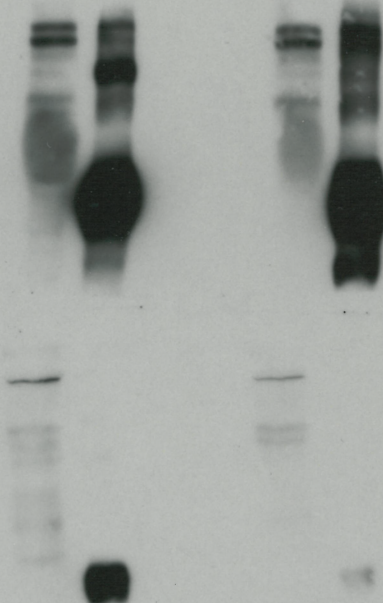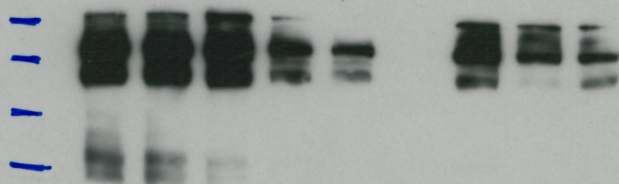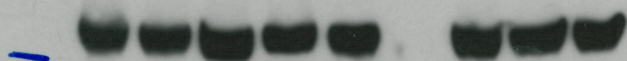

Supplement: Supplementary file 31 — Source data Fig. 3 [file 44318_2025_447_MOESM31_ESM.zip › Figure 3/Fig 3a/Fig 3a 11a 11a PDX.pdf]

in IgG

250 —  
150 —  
100 —  
75 —  
50 —

α-BCL11A

4T1

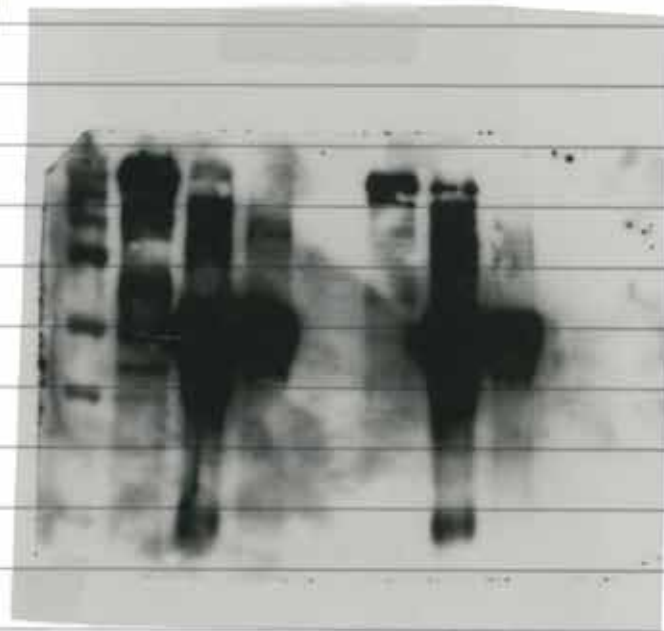

Input

Input

α-CHD2  
WB

250 —  
150 —  
100 —  
75 —  
50 —  
37 —

231

4T1

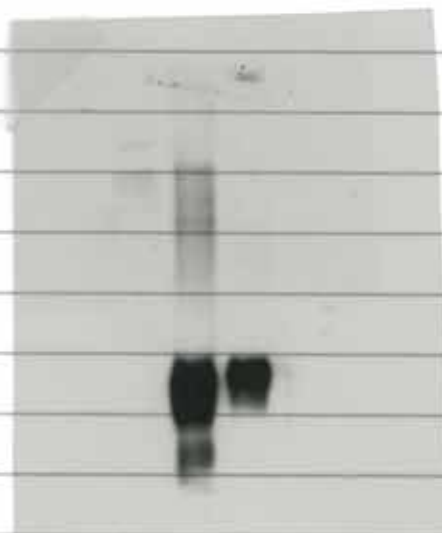

Supplement: Supplementary file 31 — Source data Fig. 3 [file 44318_2025_447_MOESM31_ESM.zip › Figure 3/Fig 3a/Fig 3a 4t1 11a 11a.pdf]

231

4T1

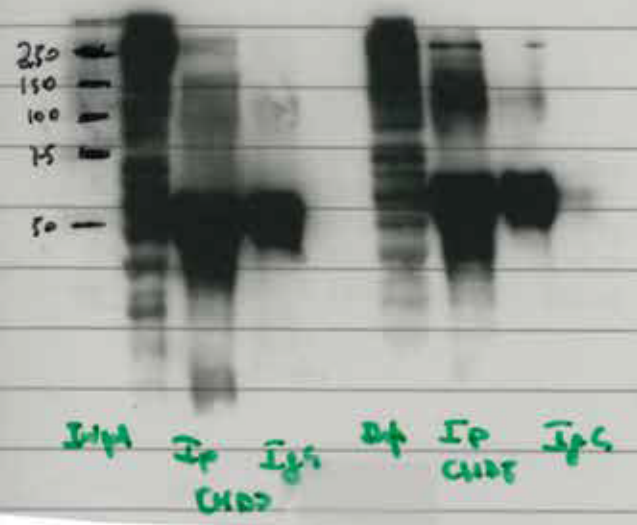

Chd8

231

4T1

WB  
beta-actin  
→ 250  
150  
100  
75  
50

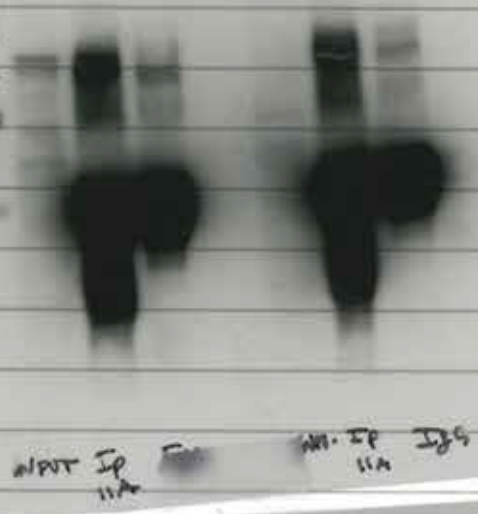

231

4T1

250  
150  
100  
75

WB  
beta-actin  
→

Input IP IgG Input IP IgG  
Chd8 Chd8 Chd8

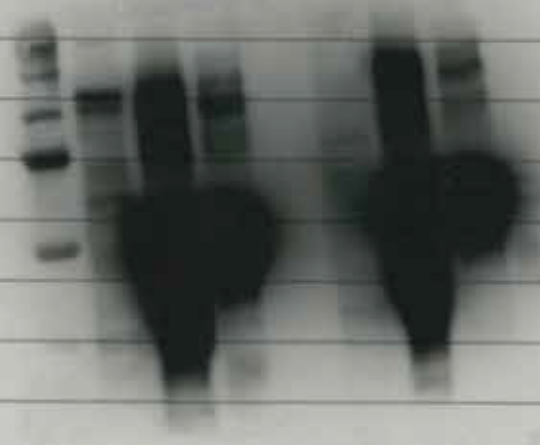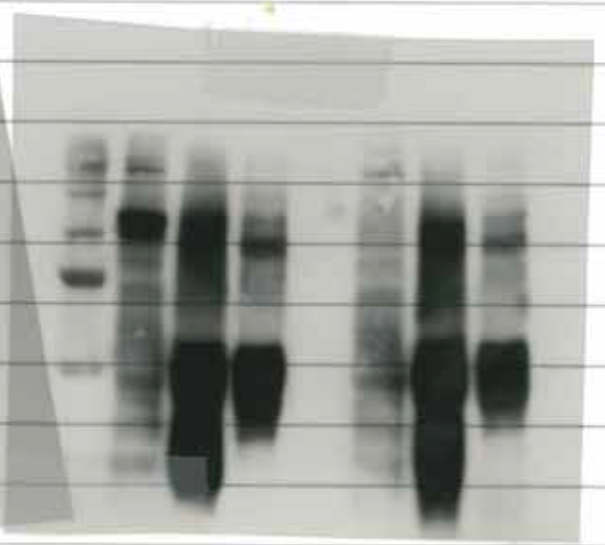

Supplement: Supplementary file 31 — Source data Fig. 3 [file 44318_2025_447_MOESM31_ESM.zip › Figure 3/Fig 3a/Fig 3a 4t1 chd8 11a and 231 IPs.pdf]

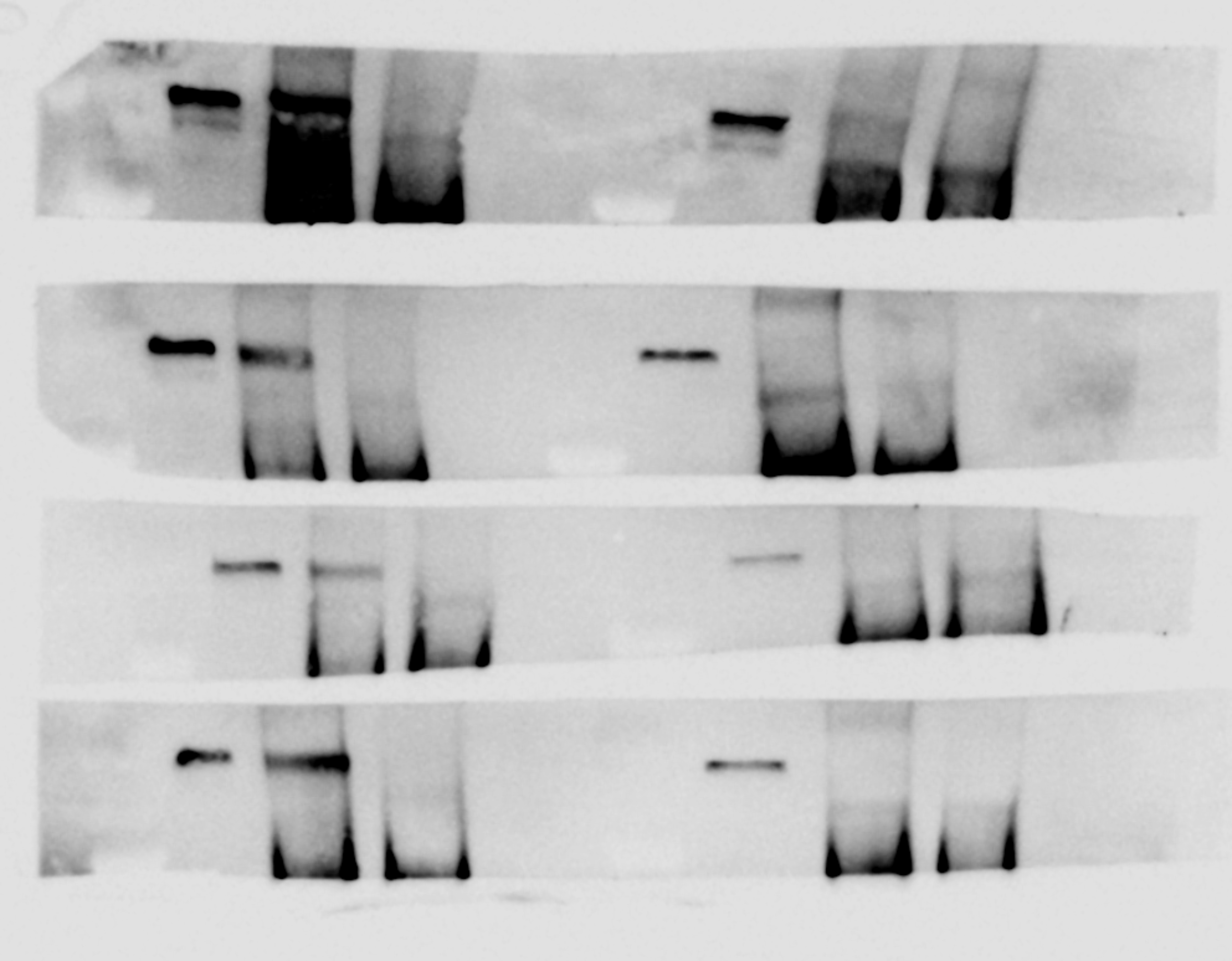

Supplement: Supplementary file 33 — Source data Fig. 5 [file 44318_2025_447_MOESM33_ESM.zip › Figure 5/Figure 5C/Original Western Blot.tif]

## Slide 1
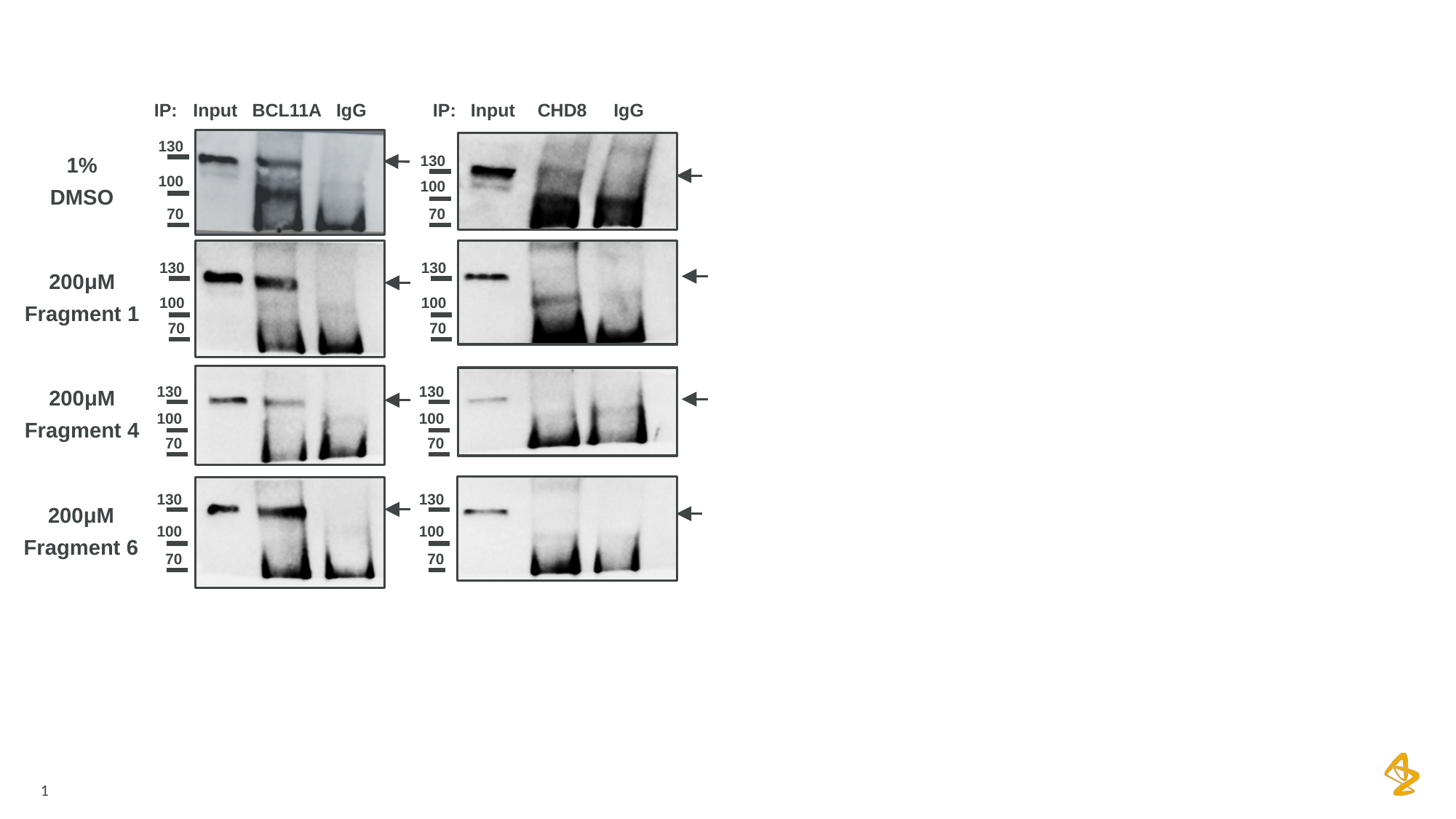

IP:
Input
BCL11A
IgG
IP:
Input
CHD8
IgG
130
130
1%
DMSO
100
100
70
70
130
130
200μM
Fragment 1
100
100
70
70
130
130
200μM
Fragment 4
100
100
70
70
130
130
200μM
Fragment 6
100
100
70
70
1

Supplement: Supplementary file 33 — Source data Fig. 5 [file 44318_2025_447_MOESM33_ESM.zip › Figure 5/Figure 5C/4T1 CoIP + fragments.pptx]

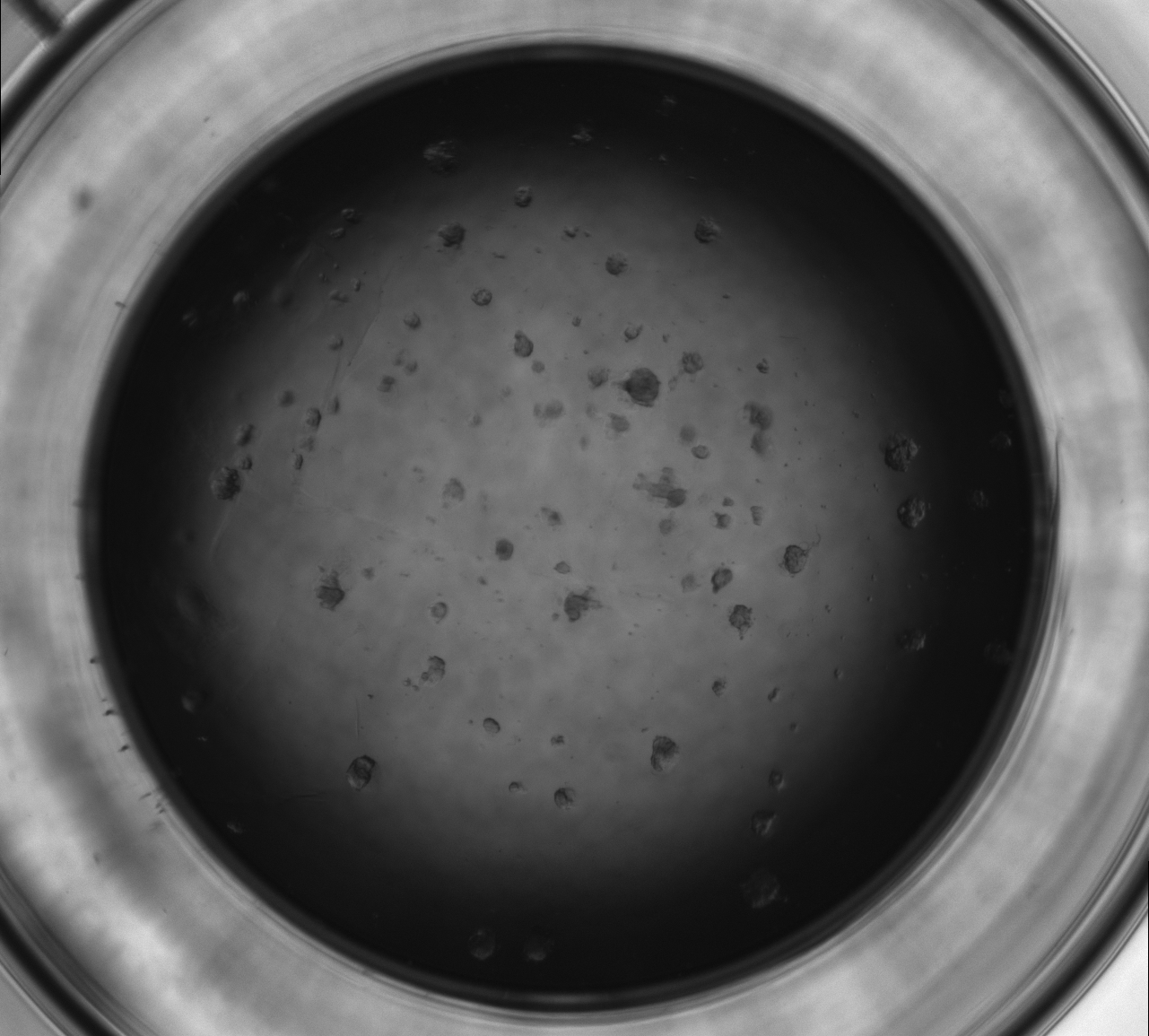

Supplement: Supplementary file 33 — Source data Fig. 5 [file 44318_2025_447_MOESM33_ESM.zip › Figure 5/Figure 5E/MCF7 Fragment 5.tif]

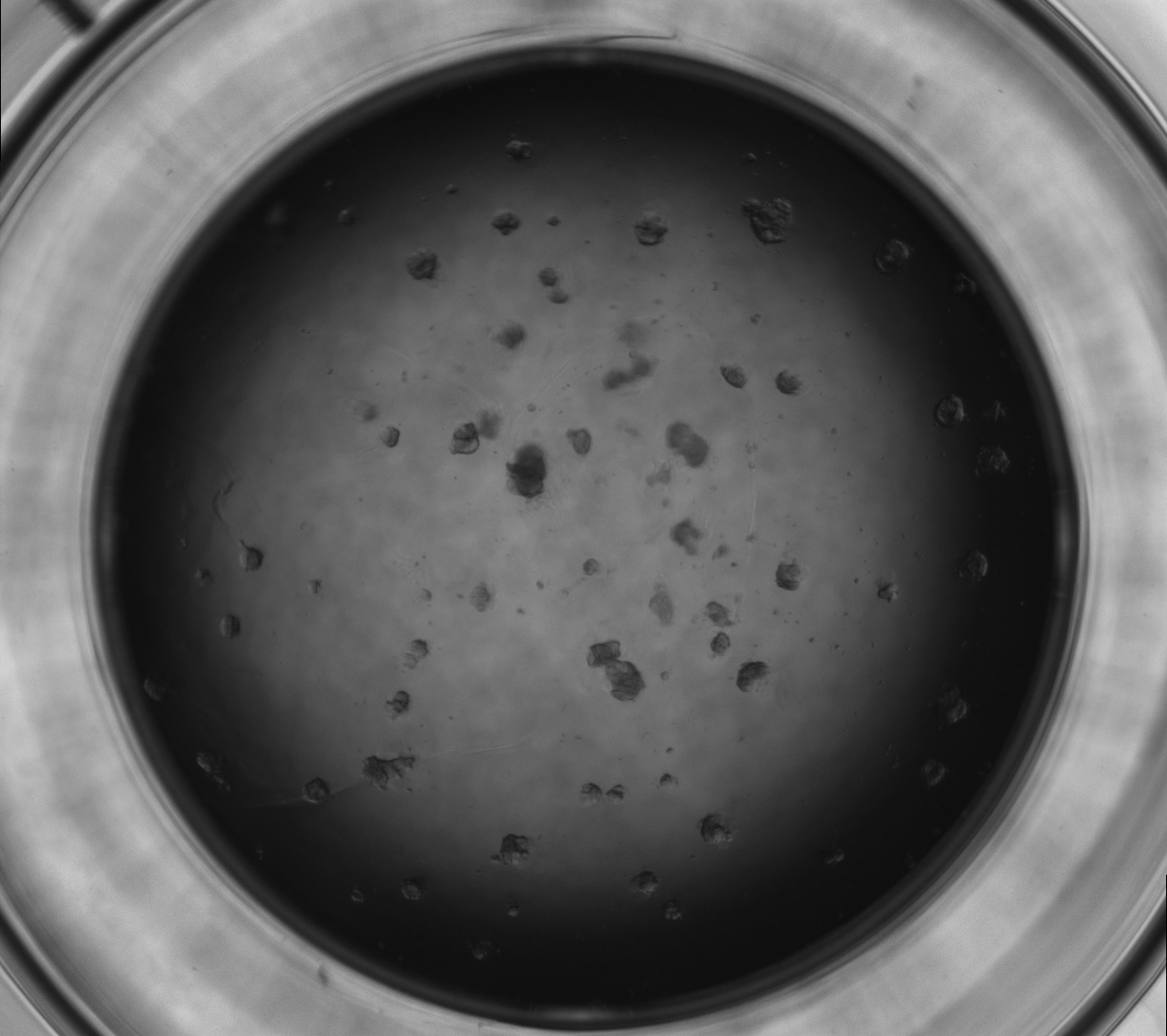

Supplement: Supplementary file 33 — Source data Fig. 5 [file 44318_2025_447_MOESM33_ESM.zip › Figure 5/Figure 5E/MCF7 Fragment 1.tif]

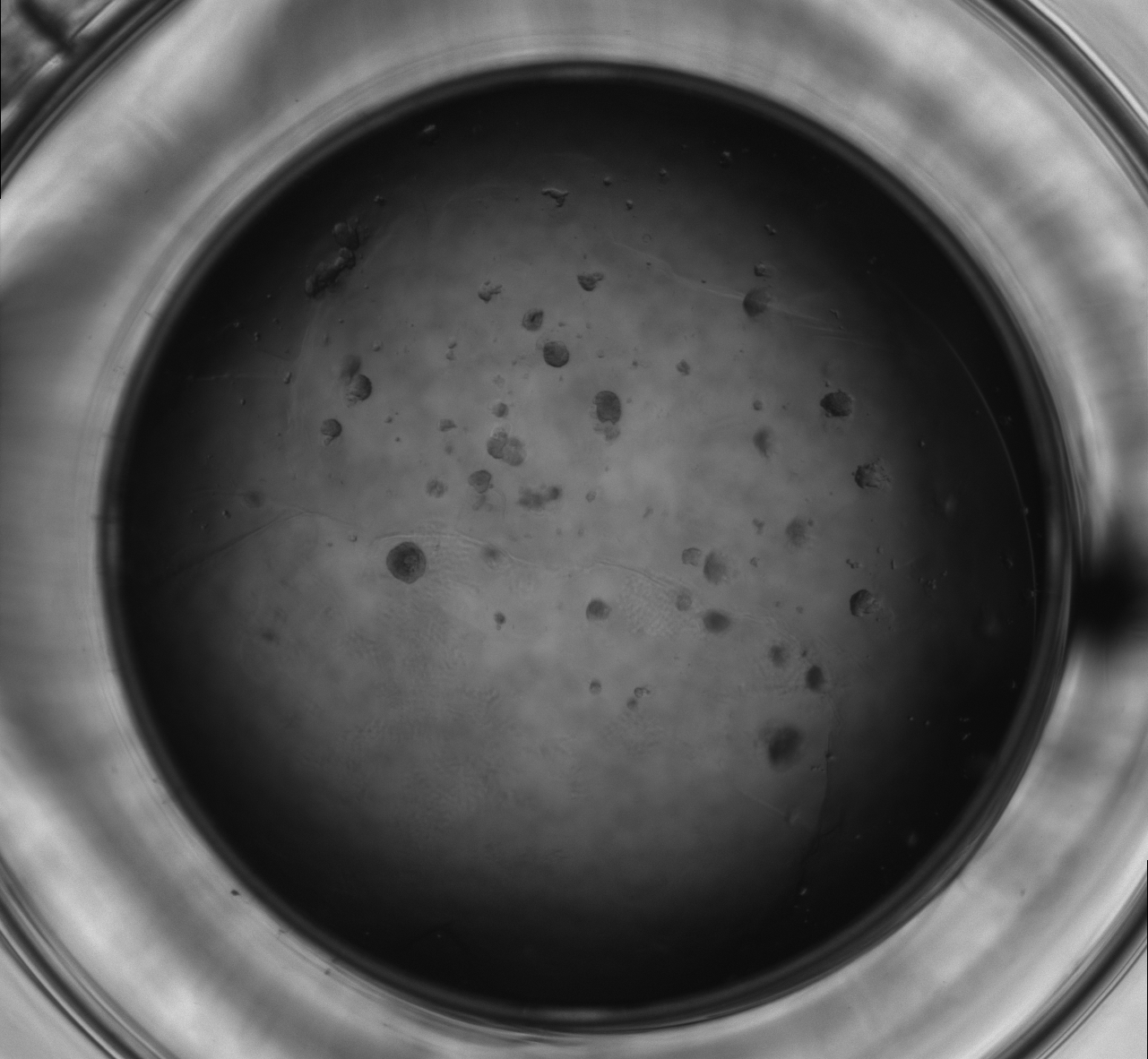

Supplement: Supplementary file 33 — Source data Fig. 5 [file 44318_2025_447_MOESM33_ESM.zip › Figure 5/Figure 5E/MCF7 DMSO.tif]

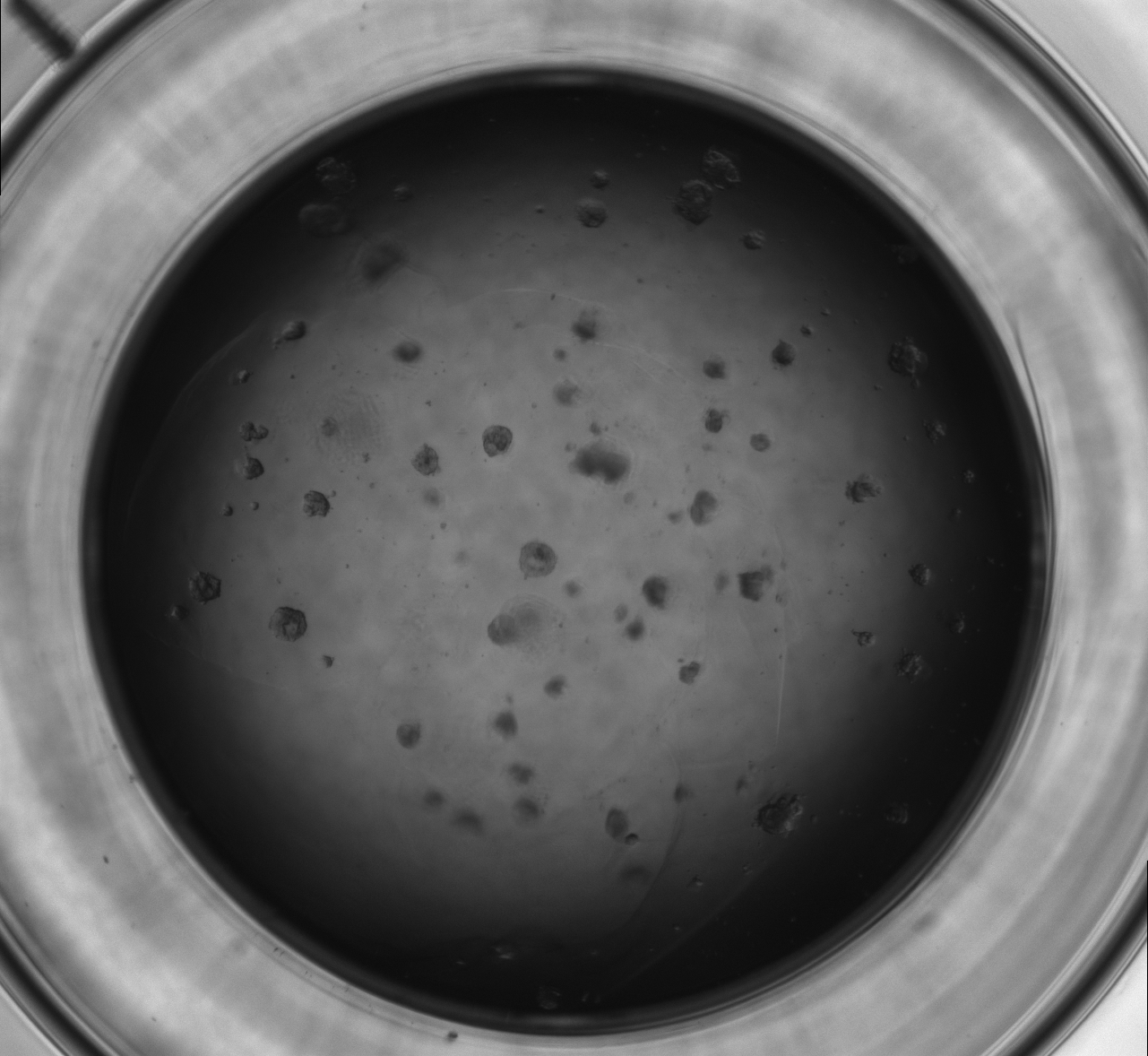

Supplement: Supplementary file 33 — Source data Fig. 5 [file 44318_2025_447_MOESM33_ESM.zip › Figure 5/Figure 5E/MCF7 Fragment 3.tif]

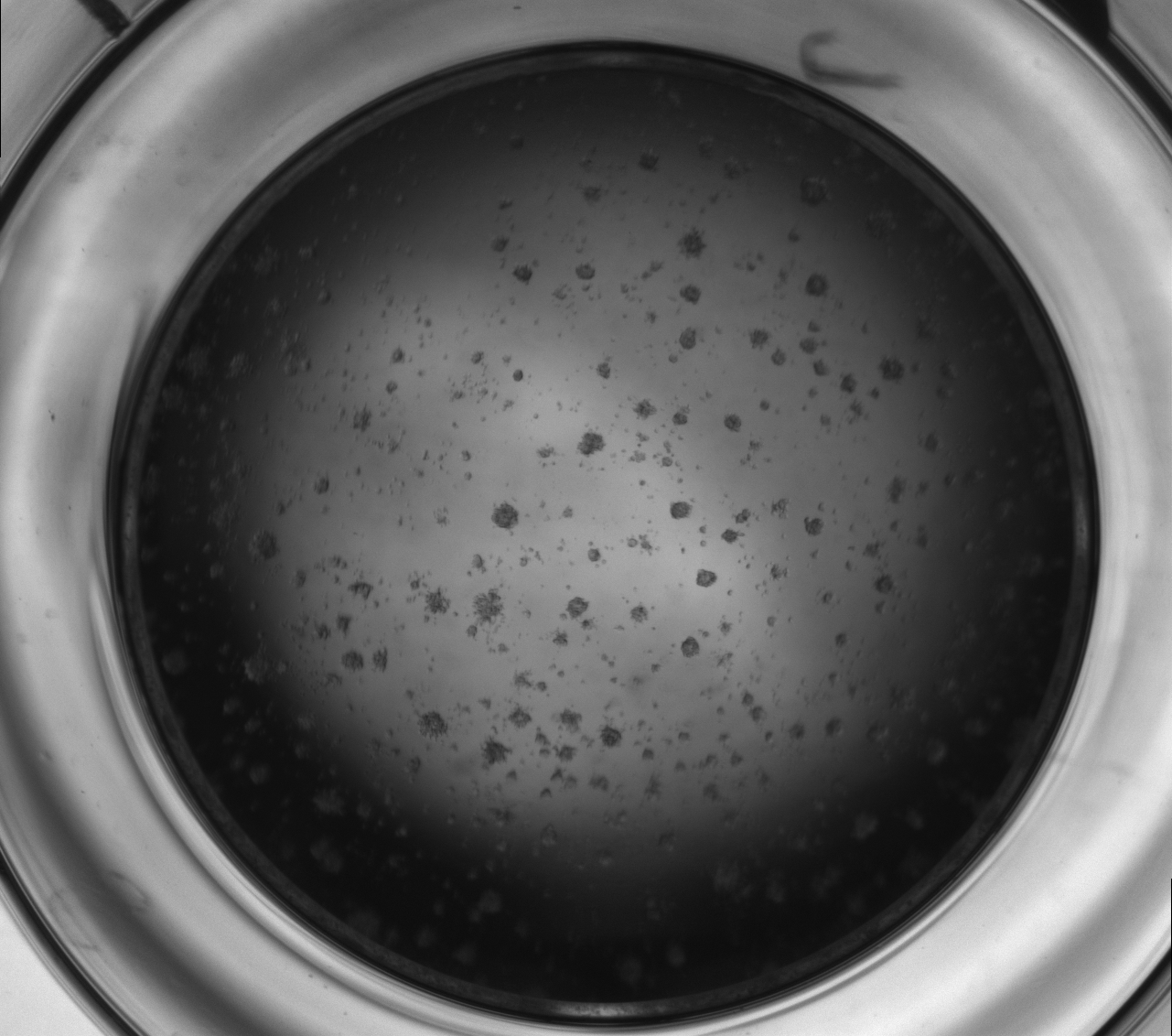

Supplement: Supplementary file 33 — Source data Fig. 5 [file 44318_2025_447_MOESM33_ESM.zip › Figure 5/Figure 5E/231 Fragment 3.tif]

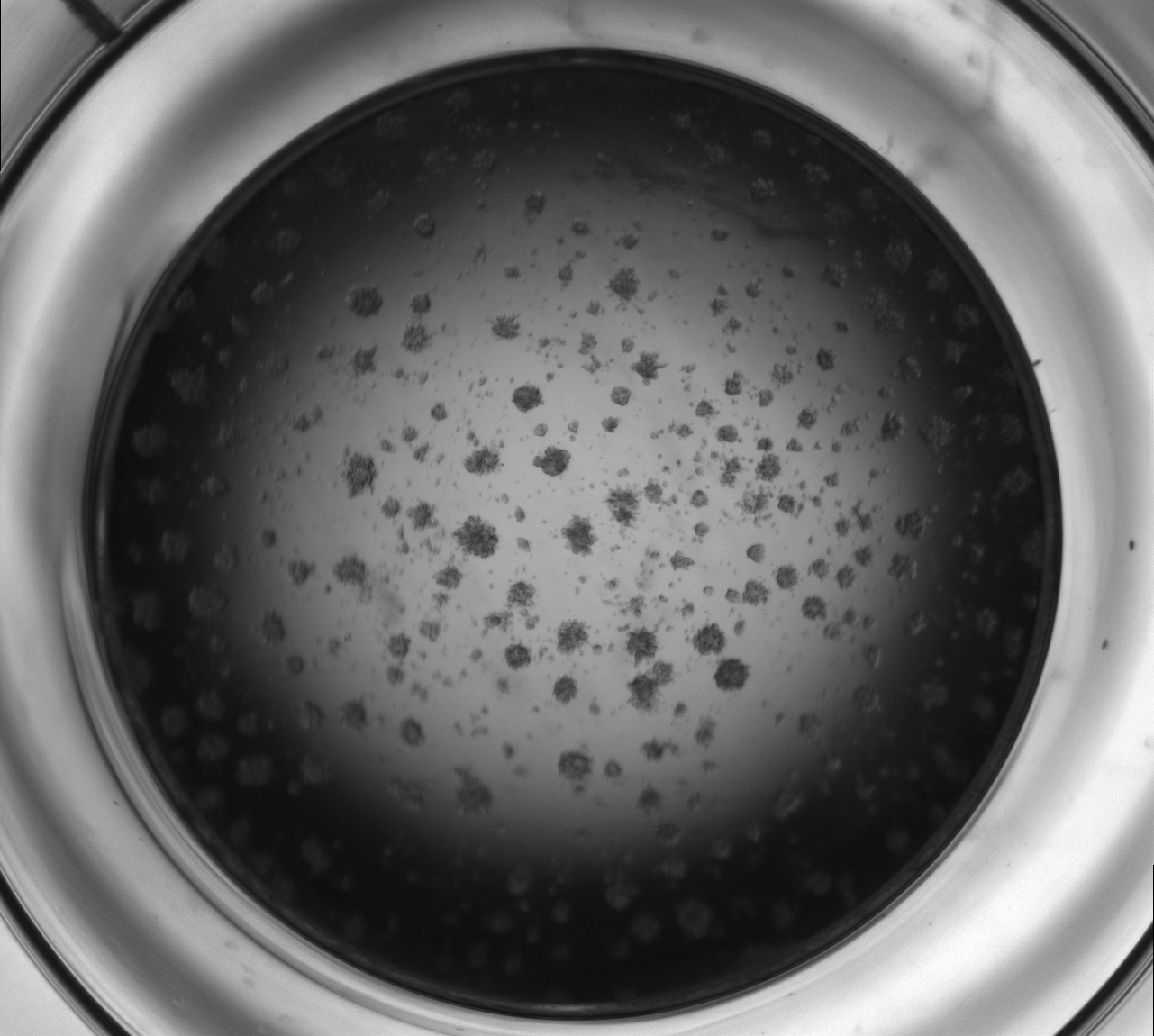

Supplement: Supplementary file 33 — Source data Fig. 5 [file 44318_2025_447_MOESM33_ESM.zip › Figure 5/Figure 5E/231 Fragment 1.tif]

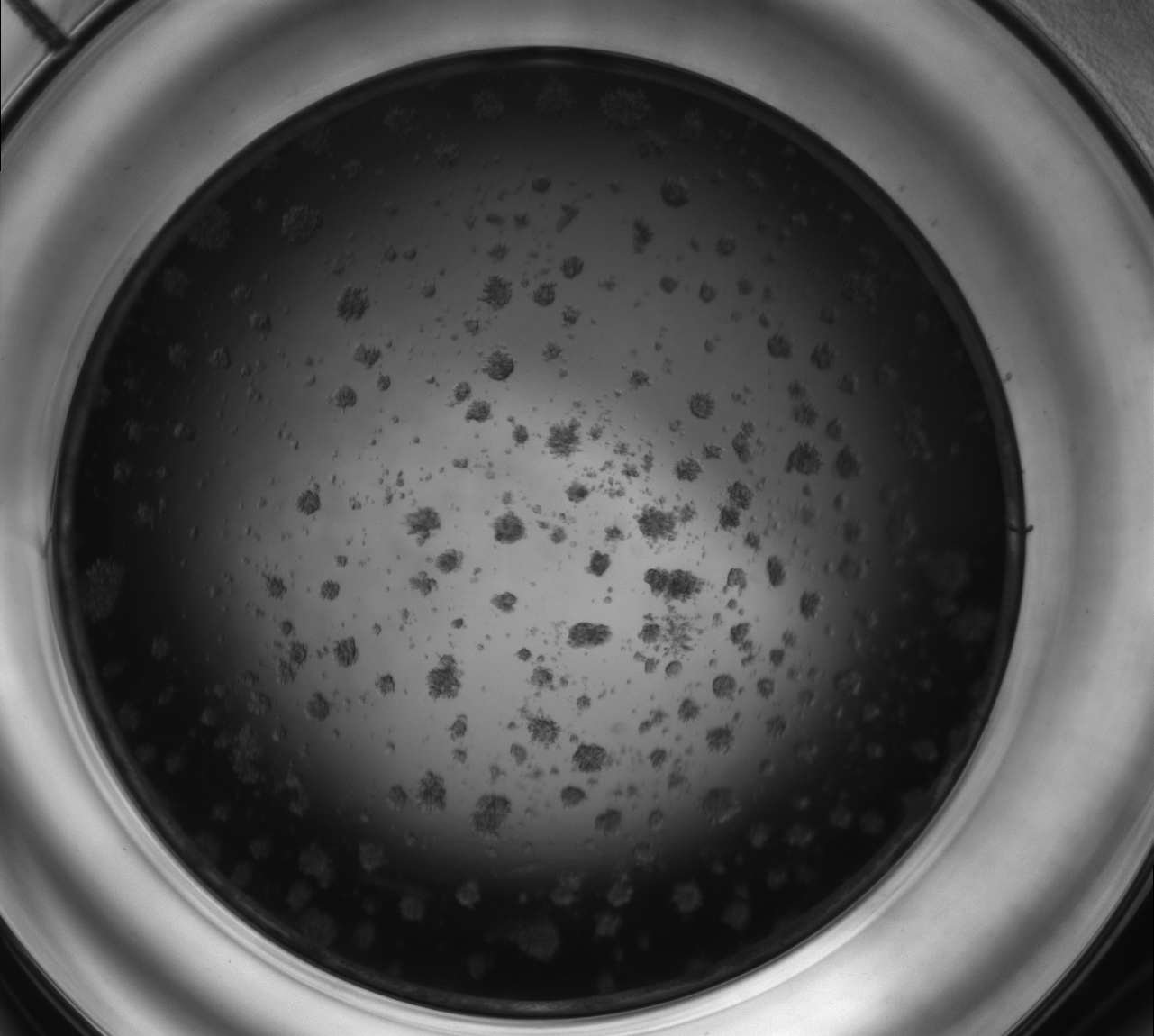

Supplement: Supplementary file 33 — Source data Fig. 5 [file 44318_2025_447_MOESM33_ESM.zip › Figure 5/Figure 5E/231 DMSO.tif]

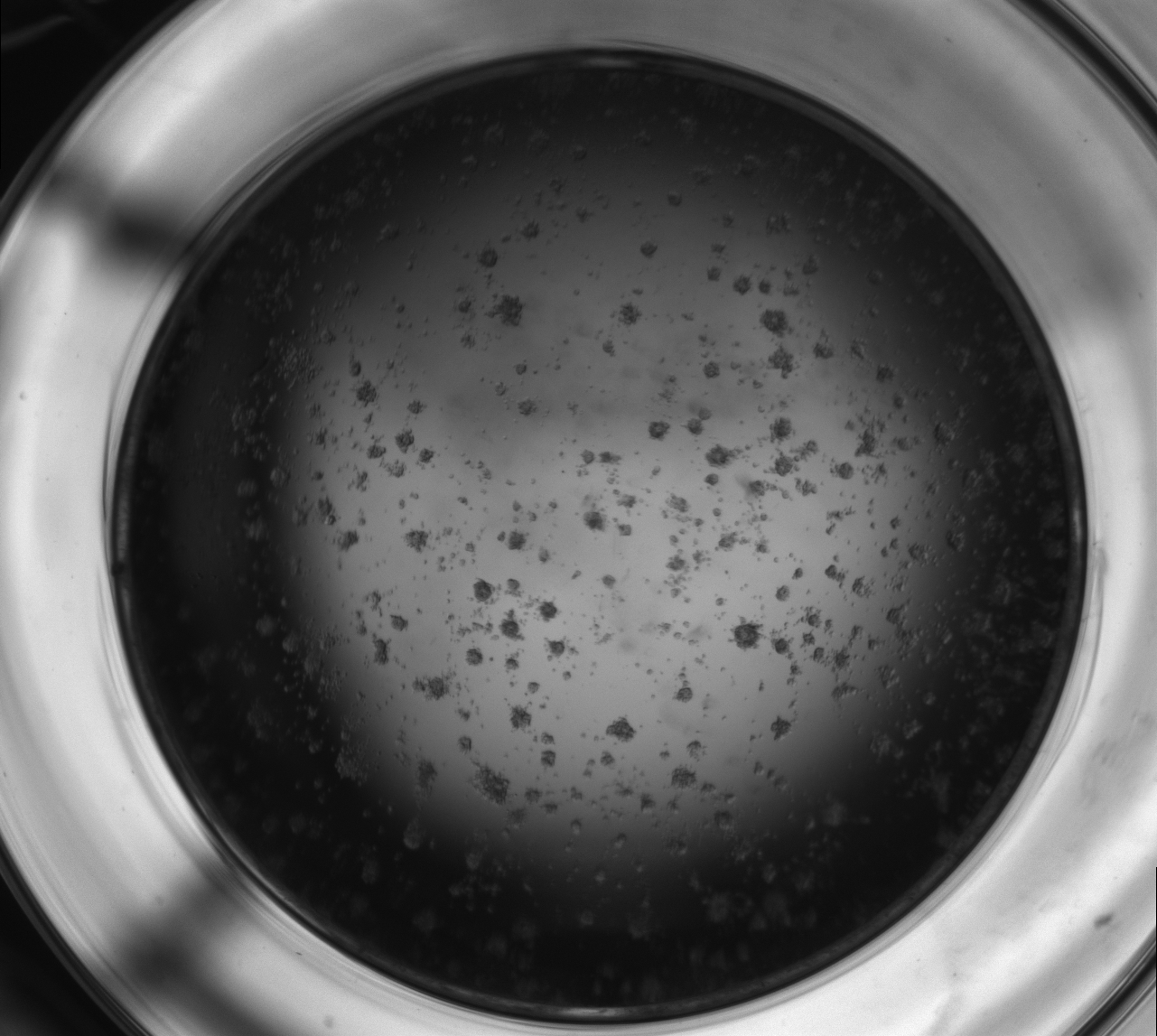

Supplement: Supplementary file 33 — Source data Fig. 5 [file 44318_2025_447_MOESM33_ESM.zip › Figure 5/Figure 5E/231 Fragment 5.tif]

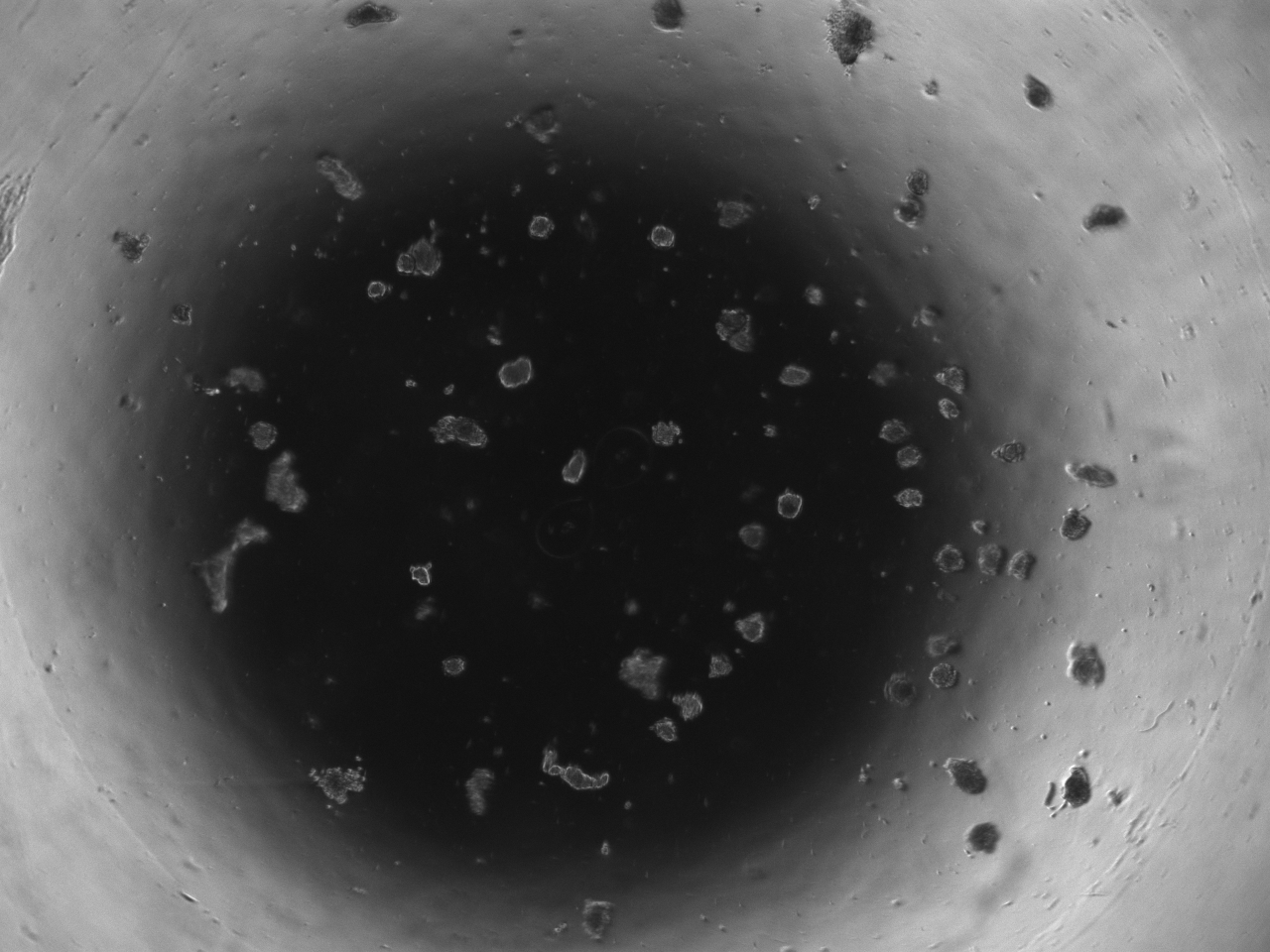

Supplement: Supplementary file 33 — Source data Fig. 5 [file 44318_2025_447_MOESM33_ESM.zip › Figure 5/Figure 5B/4T1 Fragment 5.tif]

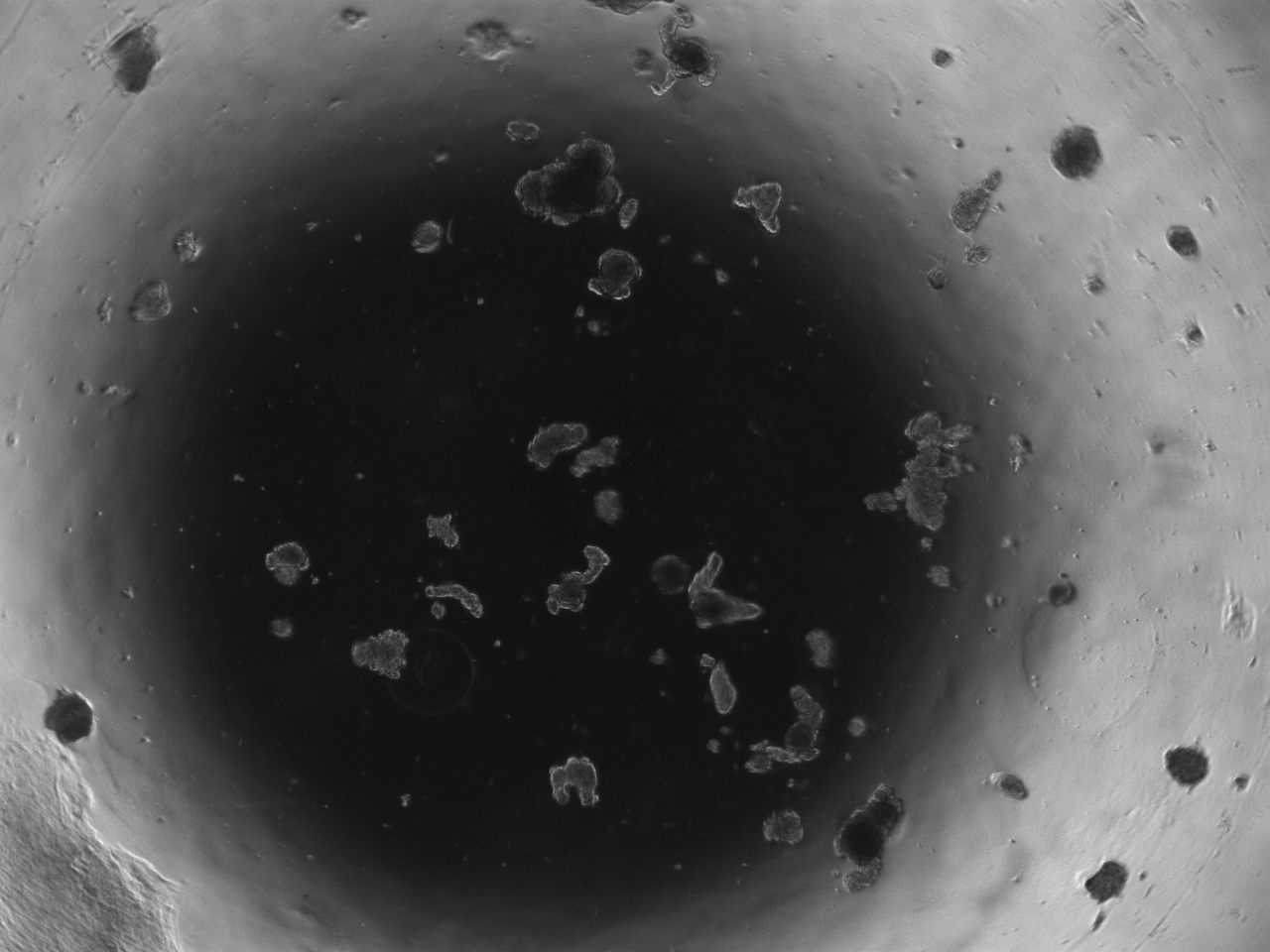

Supplement: Supplementary file 33 — Source data Fig. 5 [file 44318_2025_447_MOESM33_ESM.zip › Figure 5/Figure 5B/4T1 Fragment 4.tif]

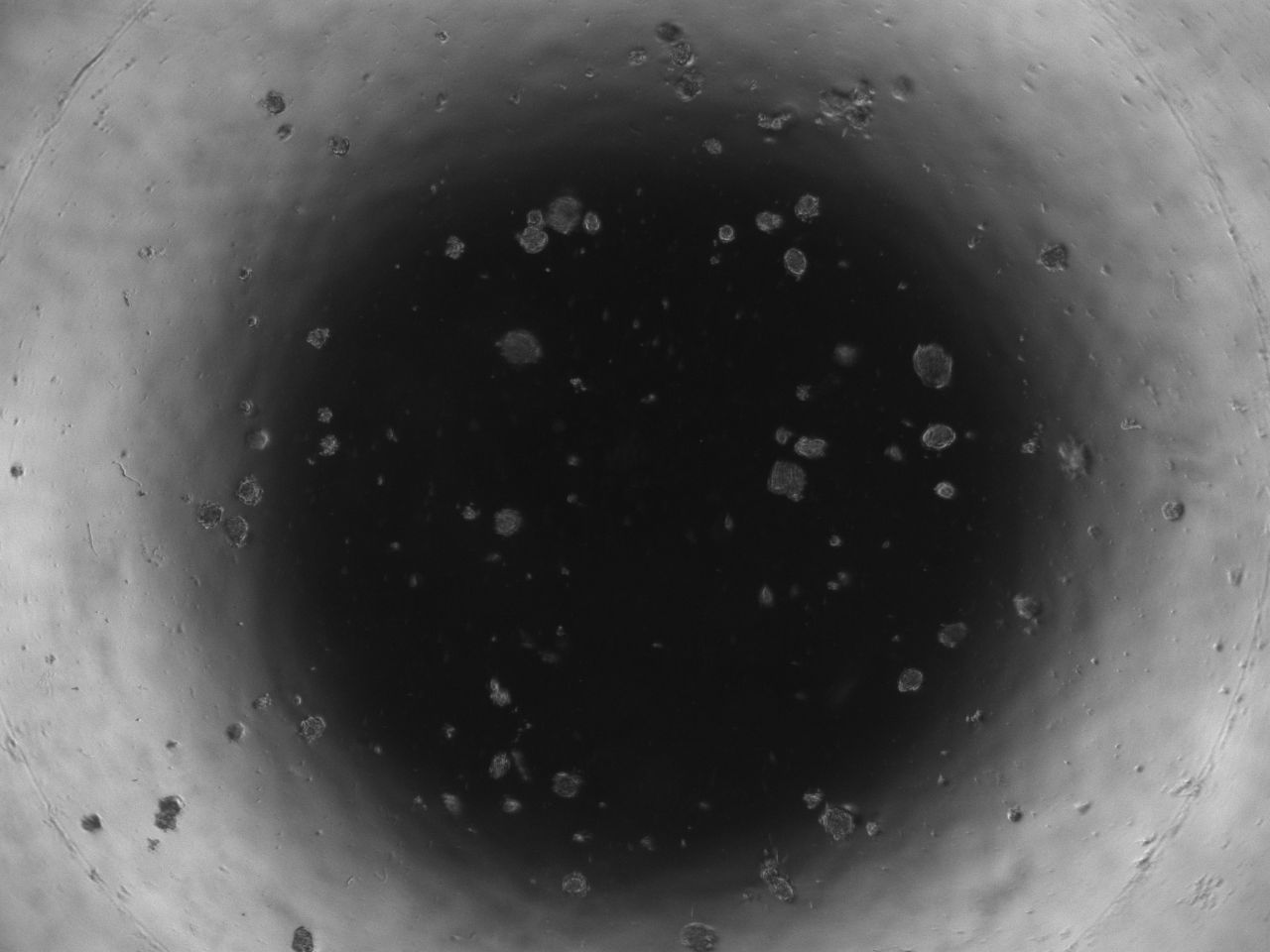

Supplement: Supplementary file 33 — Source data Fig. 5 [file 44318_2025_447_MOESM33_ESM.zip › Figure 5/Figure 5B/4T1 Fragment 3.tif]

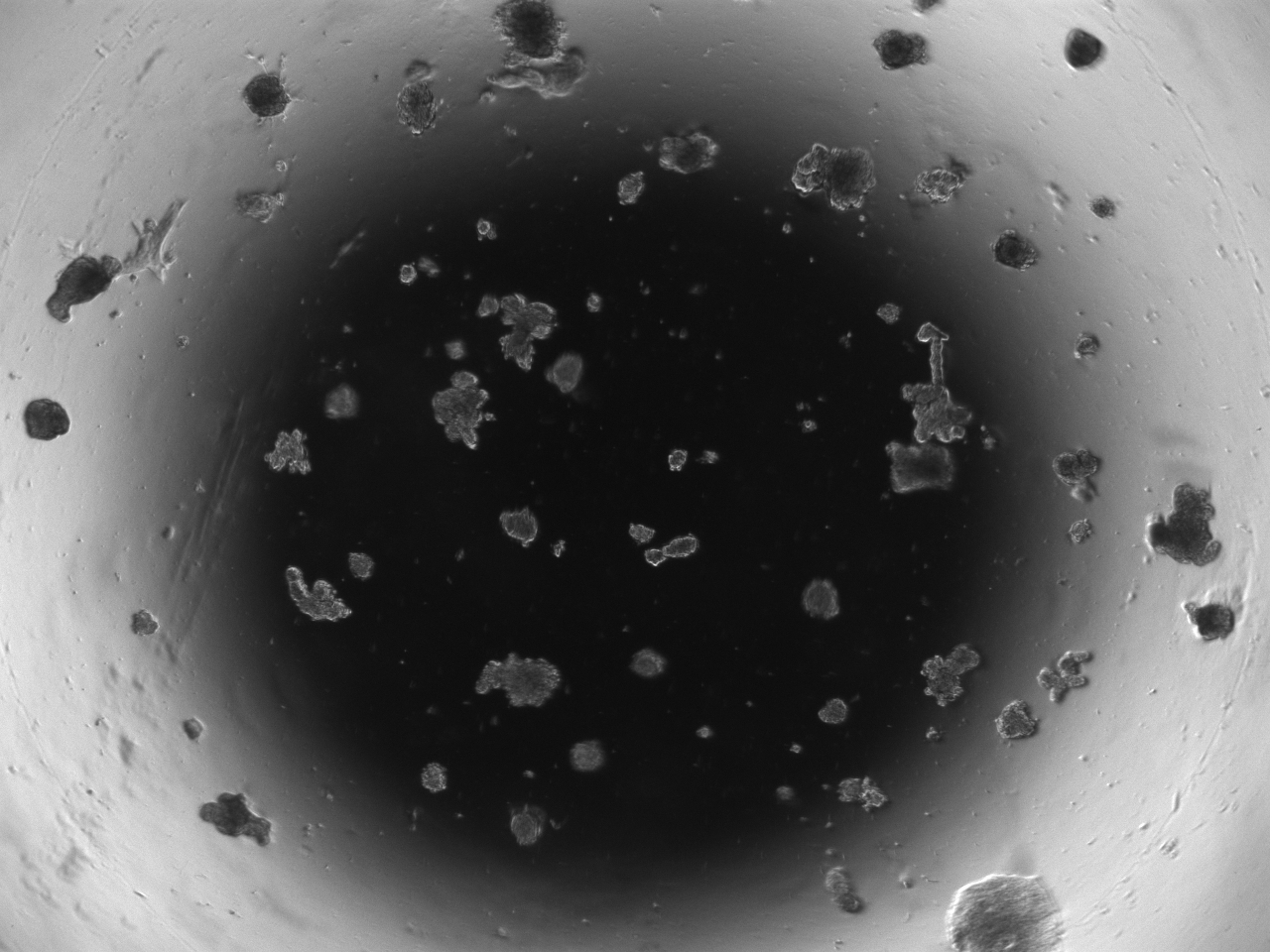

Supplement: Supplementary file 33 — Source data Fig. 5 [file 44318_2025_447_MOESM33_ESM.zip › Figure 5/Figure 5B/4T1 Fragment 2.tif]

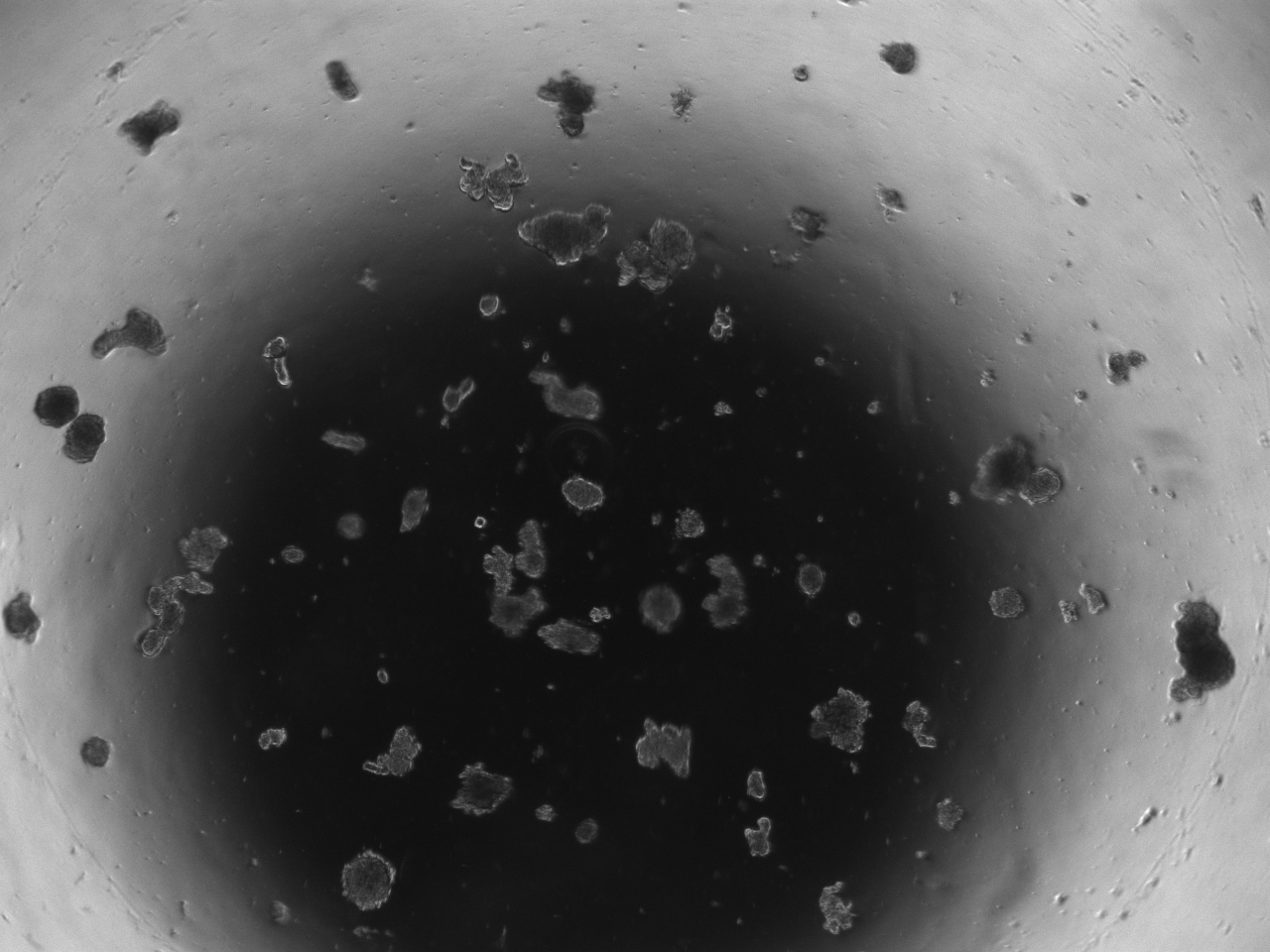

Supplement: Supplementary file 33 — Source data Fig. 5 [file 44318_2025_447_MOESM33_ESM.zip › Figure 5/Figure 5B/4T1 Fragment 1.tif]

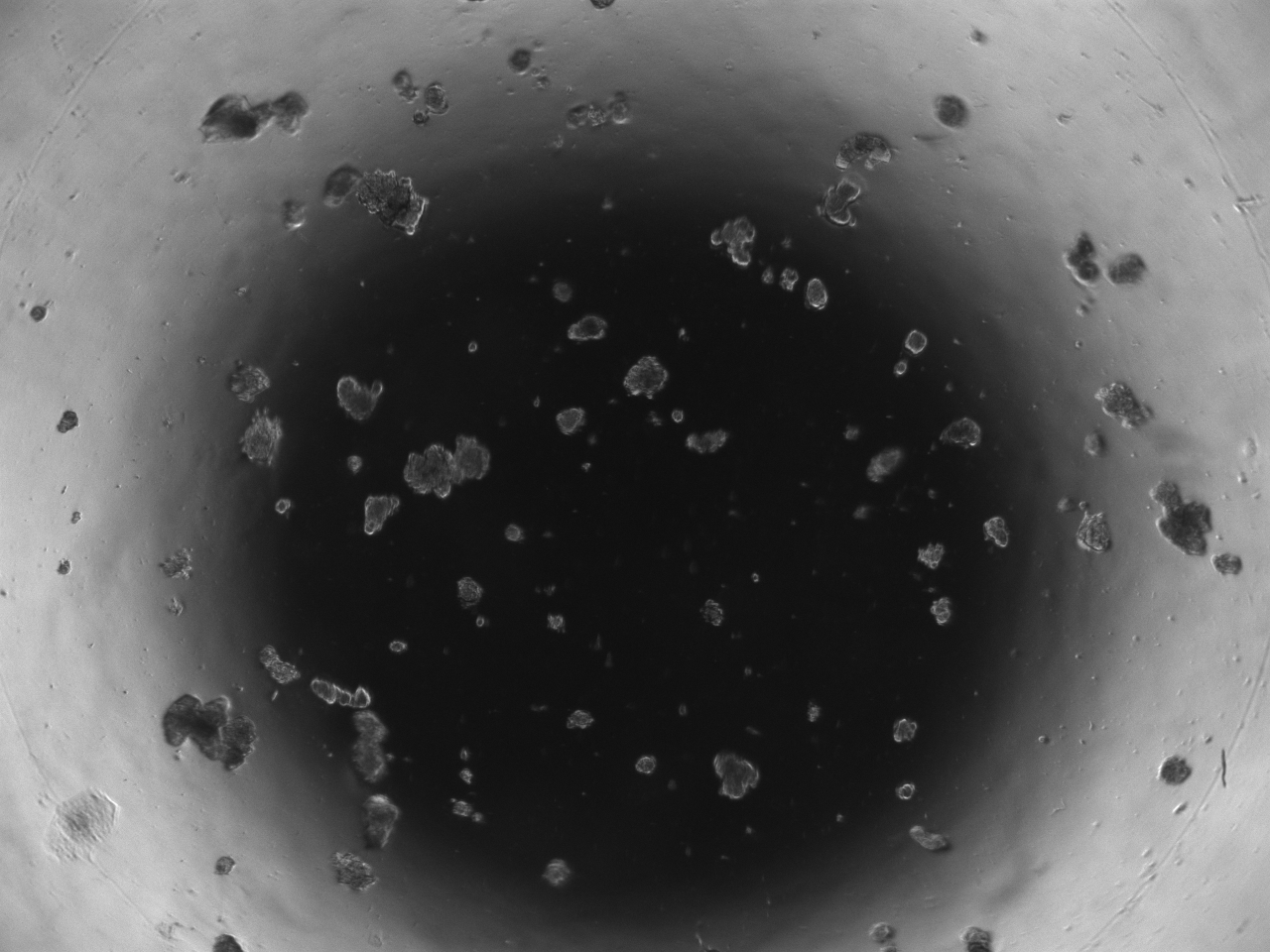

Supplement: Supplementary file 33 — Source data Fig. 5 [file 44318_2025_447_MOESM33_ESM.zip › Figure 5/Figure 5B/4T1 DMSO.tif]

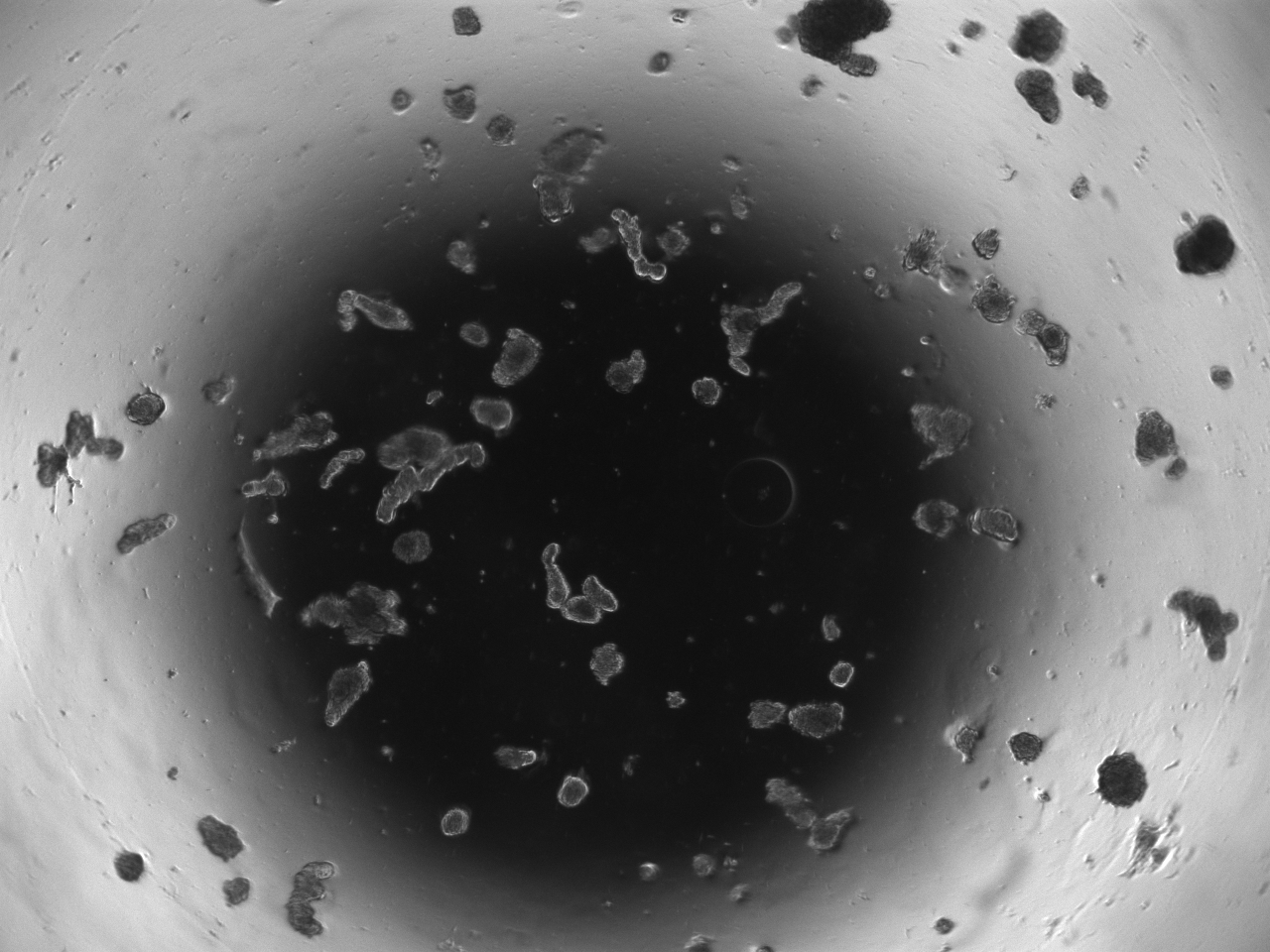

Supplement: Supplementary file 33 — Source data Fig. 5 [file 44318_2025_447_MOESM33_ESM.zip › Figure 5/Figure 5B/4T1 Fragment 8.tif]
