# Supplementary material for: CHD8 interacts with BCL11A to induce oncogenic transcription in triple negative breast cancer
Source: EMBO J. 2025 May 6;44(12):3448–67. doi: 10.1038/s44318-025-00447-8 (PMC12170886; doi:10.1038/s44318-025-00447-8)
Supplement: Supplementary file 33 — Source data Fig. 5 [file 44318_2025_447_MOESM33_ESM.zip › Figure 5/Figure 5D/Replicate FC plots/20250127_EdU005.pdf]

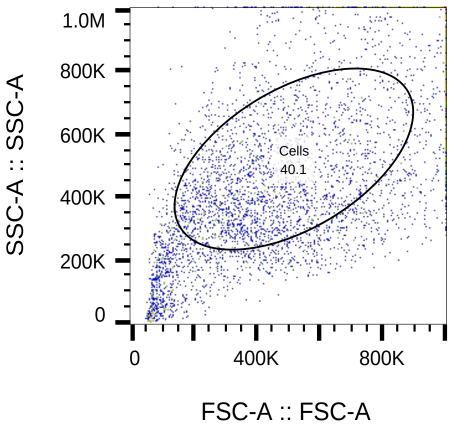


488 single colour control (+EdU -DAPI)_Data Sc
Ungated
5064

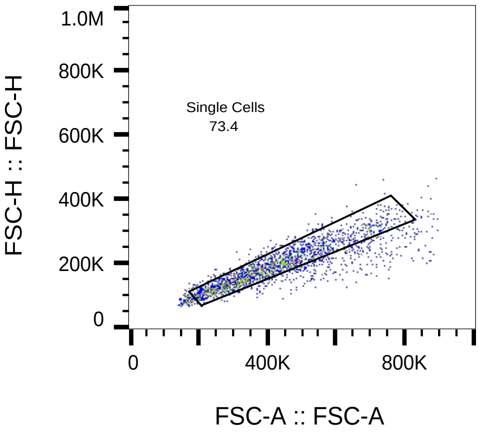


488 single colour control (+EdU -DAPI)_Data Sou
Cells
2030

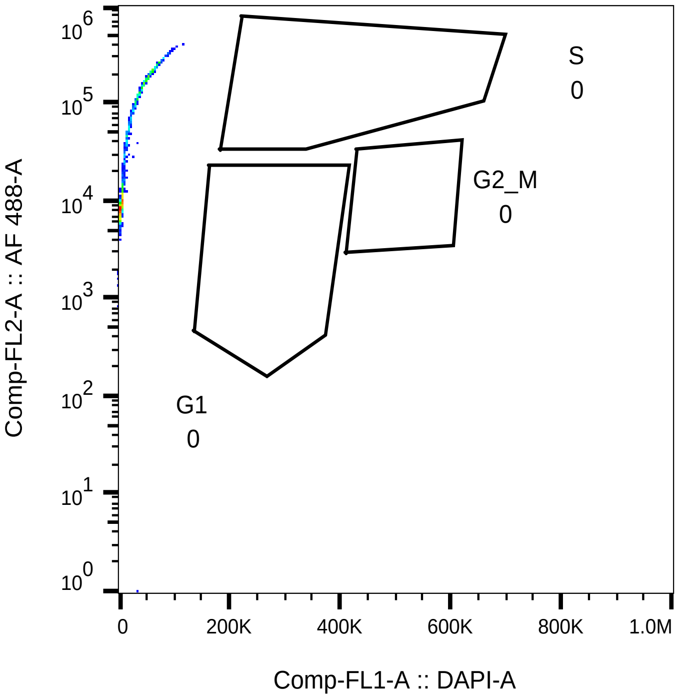


488 single colour control (+EdU -DAPI)_Data Source - 1.fcs
Single Cells
1491

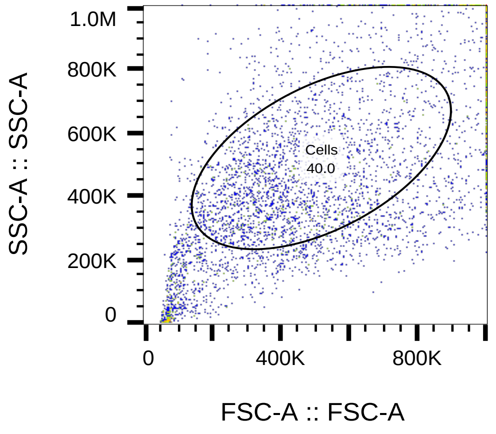


DAPI single colour control (+EdU -488) - 1_Data
Ungated
5225

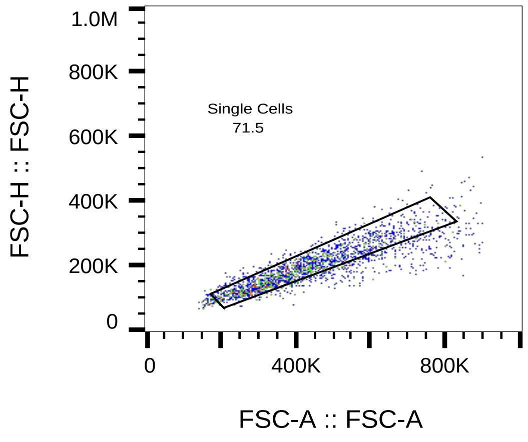


DAPI single colour control (+EdU -488
Cells
2089

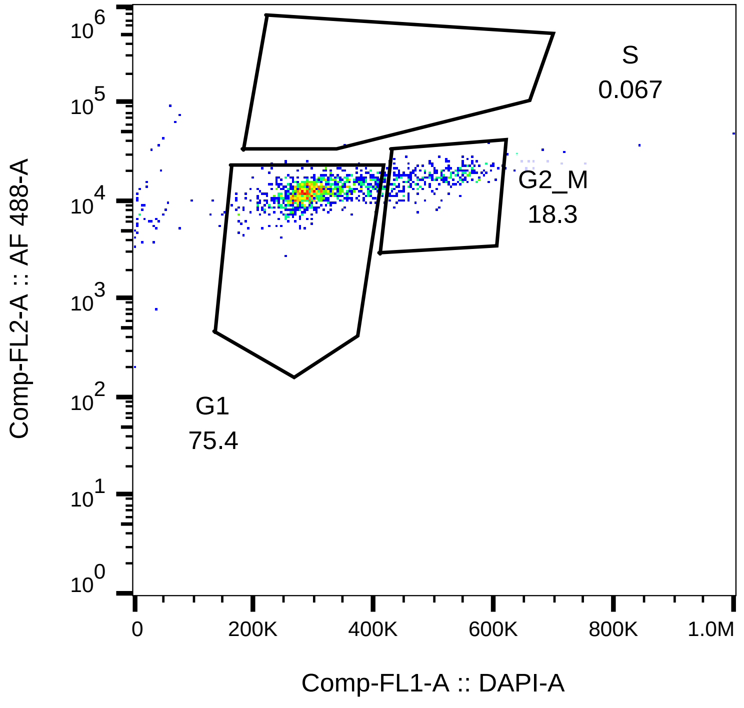


DAPI single colour control (+EdU -488) - 1_Data Source - 1.fcs
Single Cells
1494

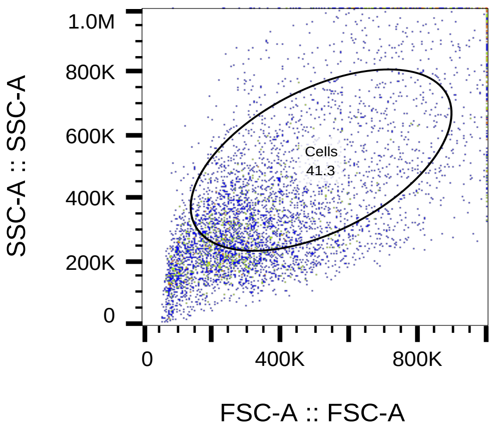


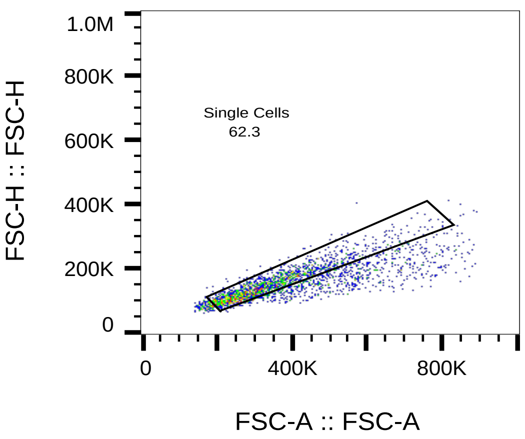


DAPI single colour control (+EdU -488)_Data Sc
Ungated
5654

DAPI single colour control (+EdU -48
Cells
2333

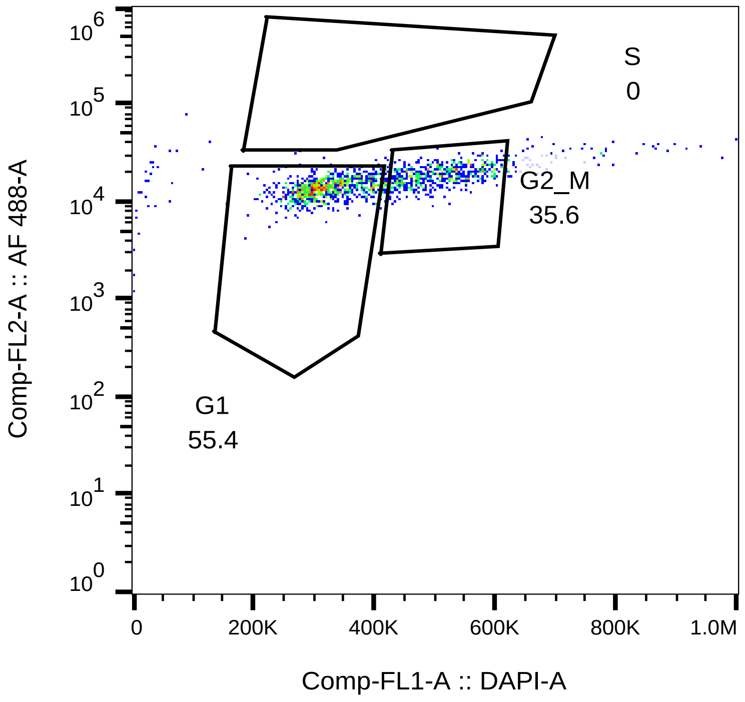


DAPI single colour control (+EdU -488)_Data Source - 1.fcs
Single Cells
1453

Paper labelling- treatment DMSO

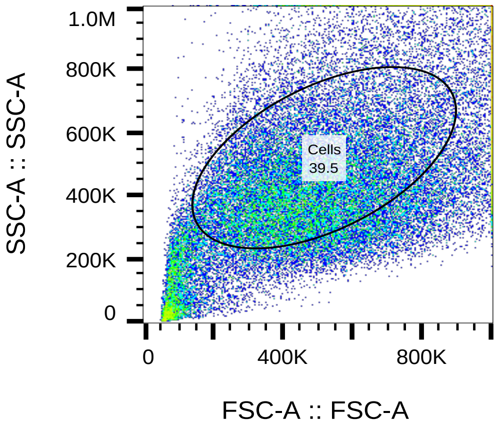


DMSO +EdU_Data Source - 1.fcs
Ungated
53814

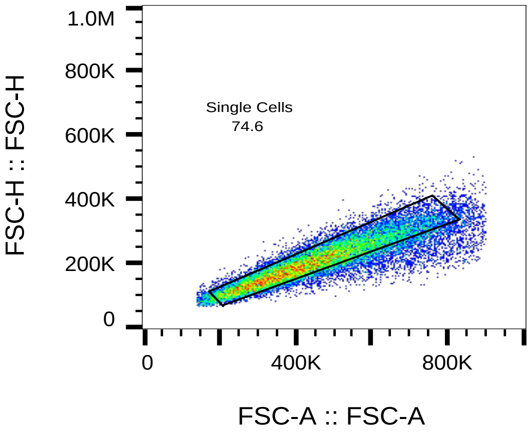


DMSO +EdU_Data Source - 1.fcs
Cells
21232

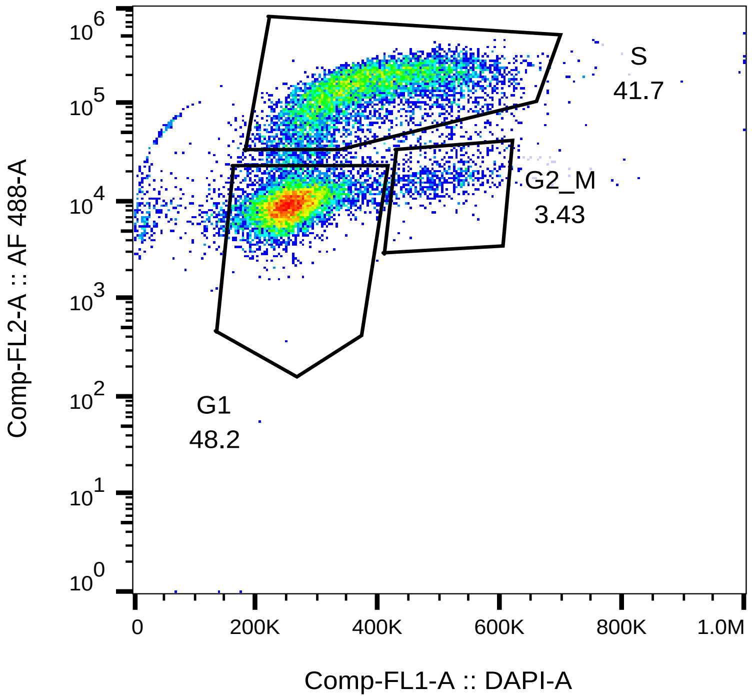


DMSO +EdU_Data Source - 1.fcs
Single Cells
15838

Paper labelling- treatment DMSO EdU negative contro

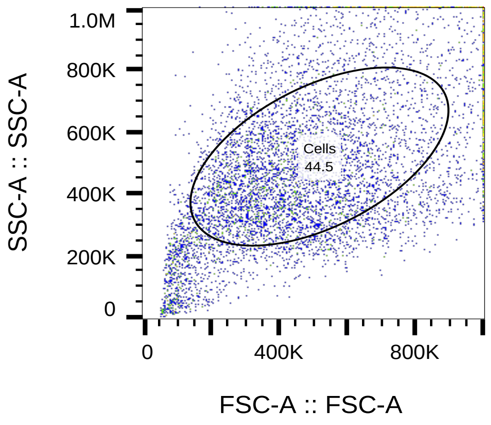


DMSO -EdU_Data Source - 1.fcs
Ungated
8271

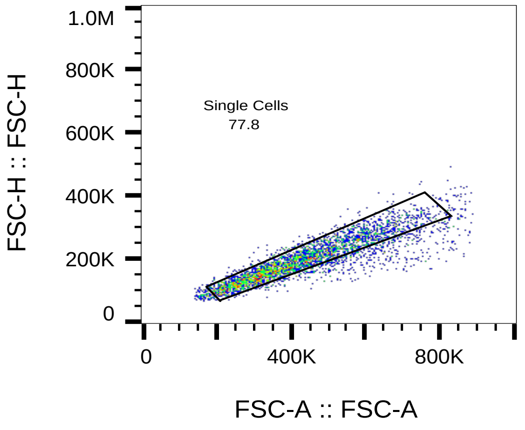


DMSO -EdU_Data Source - 1.fcs
Cells
3683

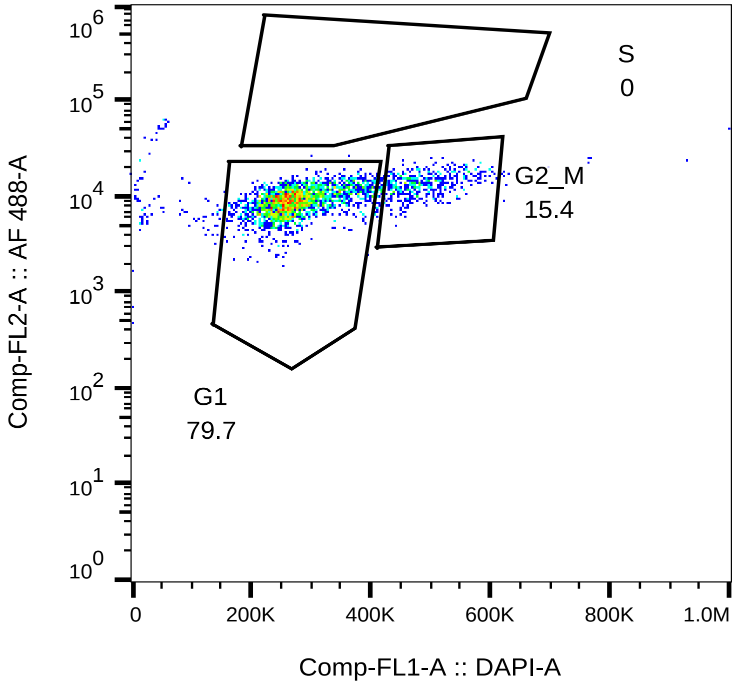


DMSO -EdU_Data Source - 1.fcs
Single Cells
2866

Paper labelling- treatment 1

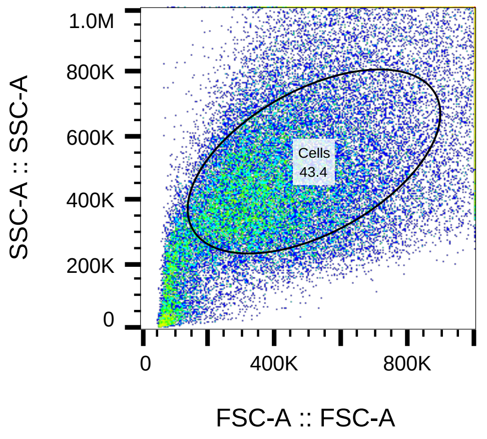


Fragment 1 +EdU_Data Source - 1.fcs
Ungated
50713

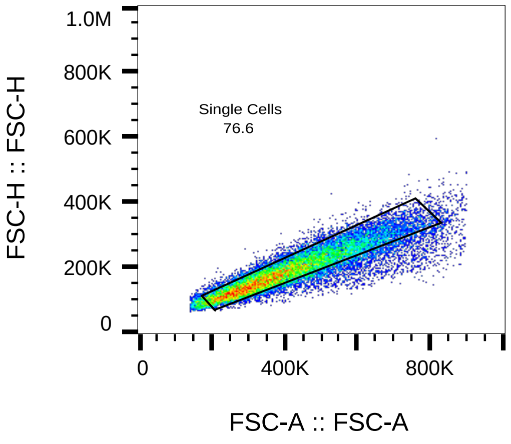


Fragment 1 +EdU_Data Source - 1.fcs
Cells
22024

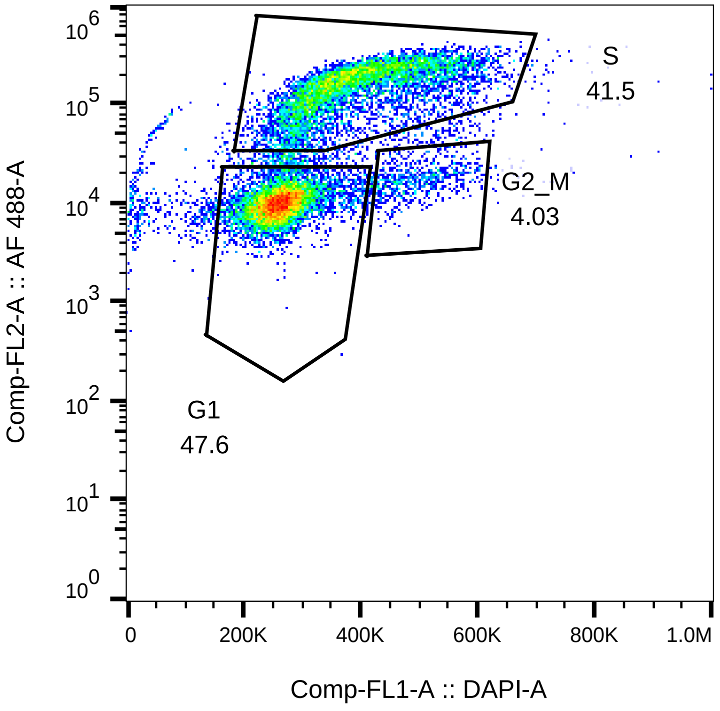


Fragment 1 +EdU_Data Source - 1.fcs
Single Cells
16874

Paper labelling- treatment 1 EdU negative control

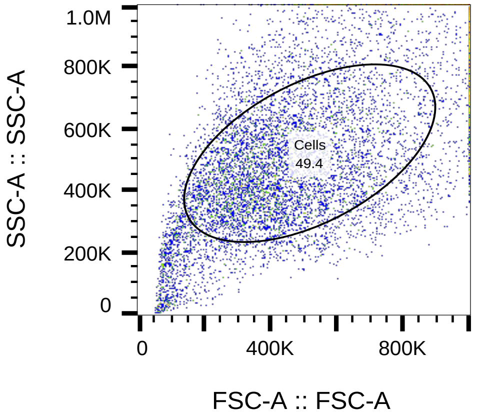


Fragment 1 -EdU_Data Source - 1.fcs
Ungated
9860

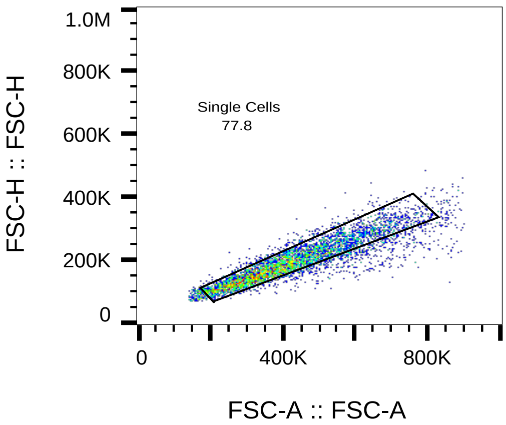


Fragment 1 -EdU_Data Source - 1.fcs
Cells
4875

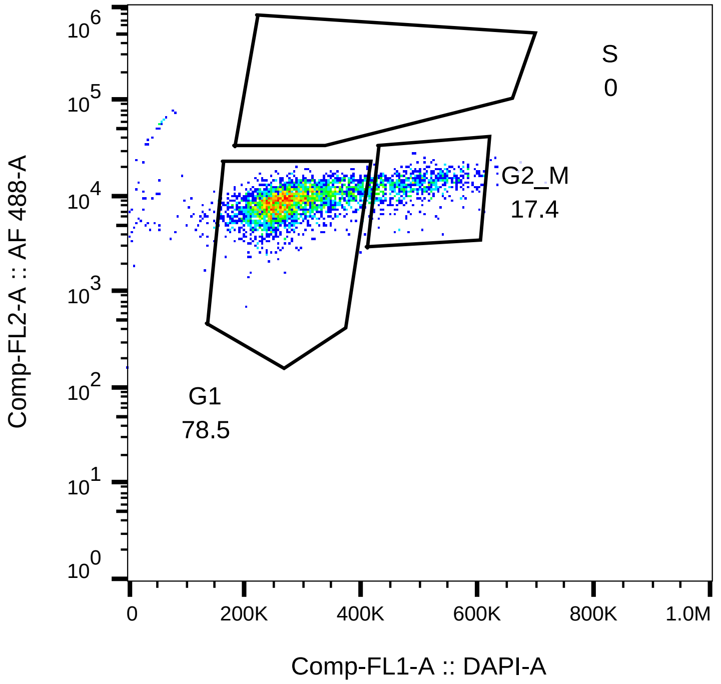


Fragment 1 -EdU_Data Source - 1.fcs
Single Cells
3793

Paper labelling- treatment 3

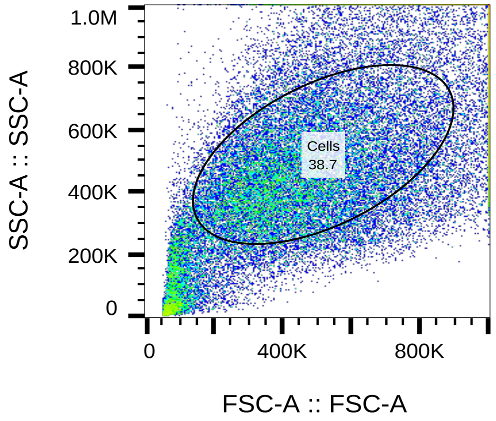


Fragment 4 +EdU _Data Source - 1.fcs
Ungated
51738

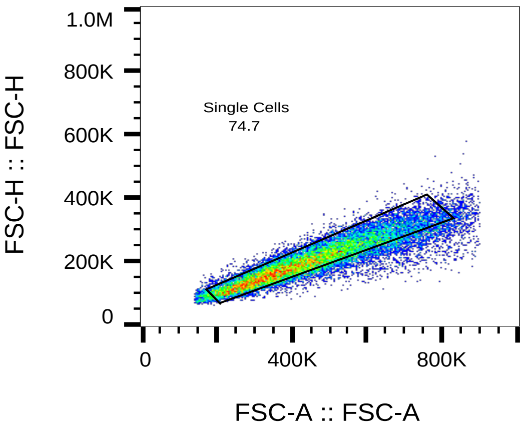


Fragment 4 +EdU _Data Source - 1.f
Cells
20034

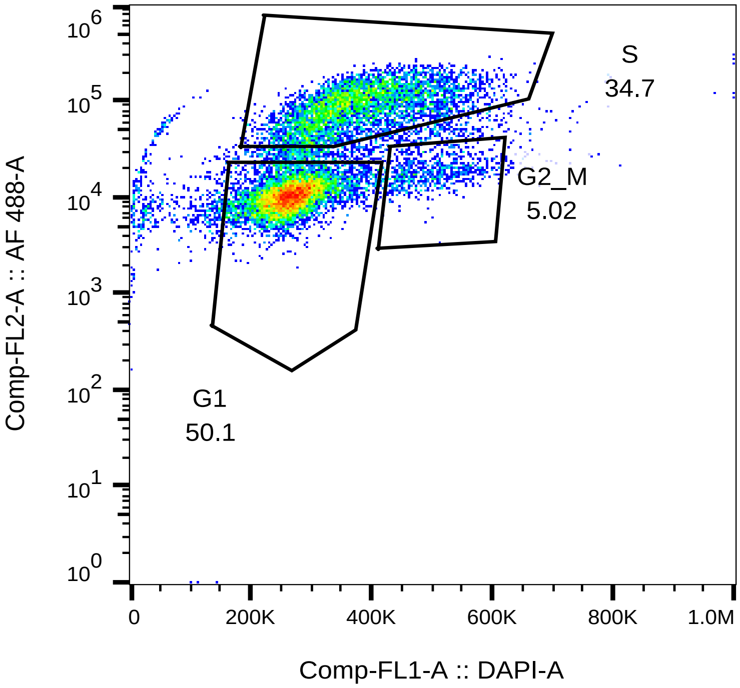


Fragment 4 +EdU _Data Source - 1.fcs
Single Cells
14960

Paper labelling- treatment 3 EdU negative control

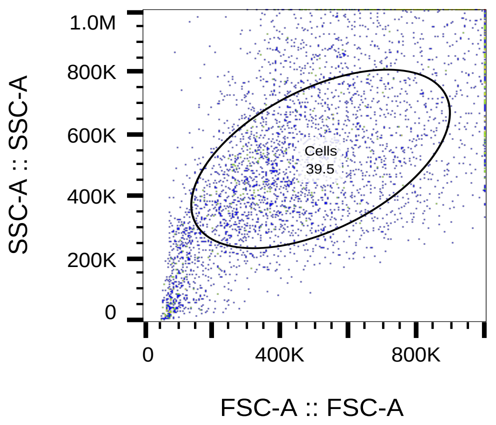


Fragment 4 -EdU _Data Source - 1.fcs
Ungated
5284

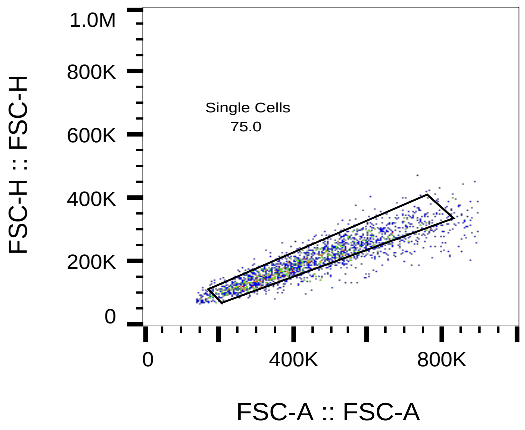


Fragment 4 -EdU _Data Source - 1.fcs
Cells
2087

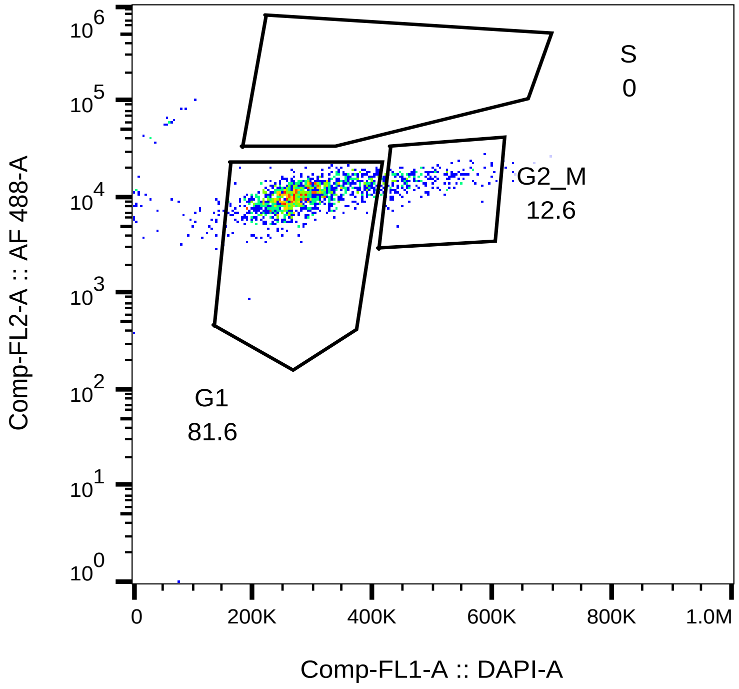


Fragment 4 -EdU _Data Source - 1.fcs
Single Cells
1565

Paper labelling- treatment fragment 5

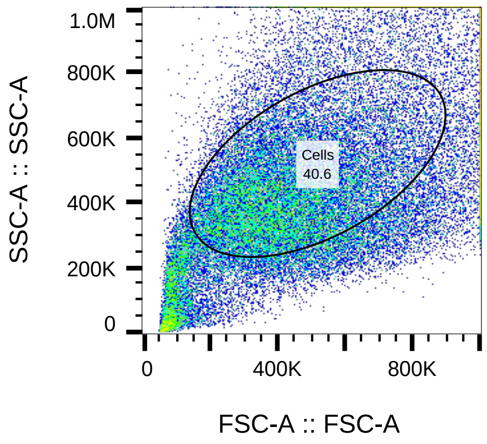


Fragment 6 +EdU_Data Source - 1.fcs
Ungated
52845

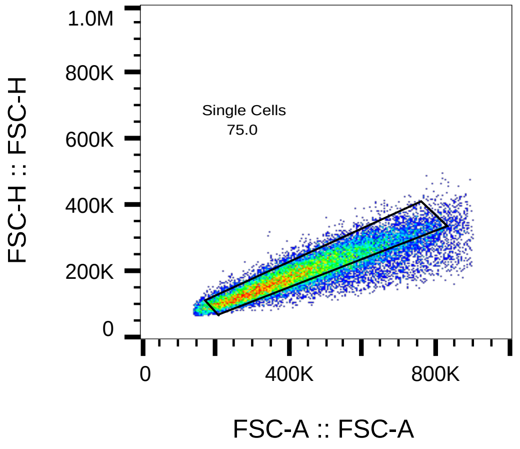


Fragment 6 +EdU_Data Source - 1.fcs
Cells
21458

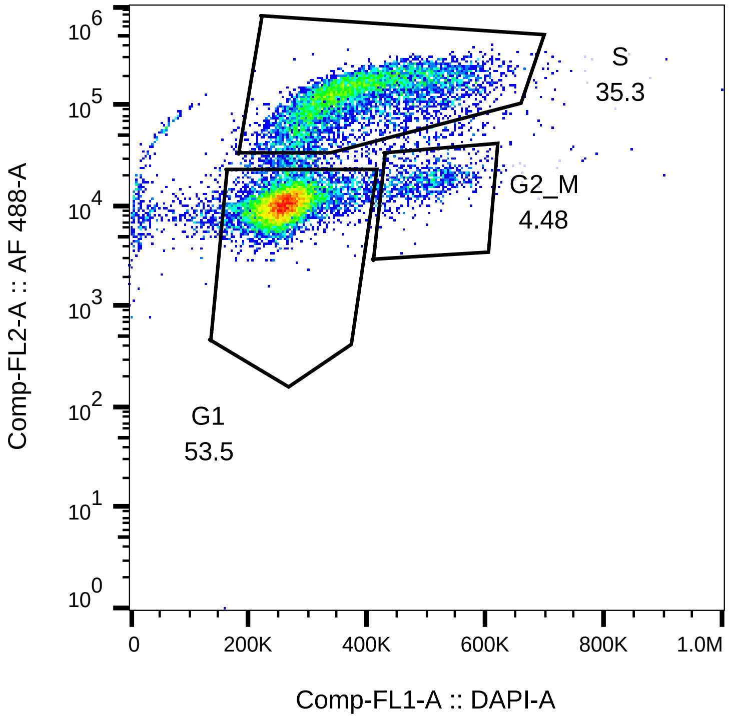


Fragment 6 +EdU_Data Source - 1.fcs
Single Cells
16084

Paper labelling- treatment fragment 5 EdU negative control

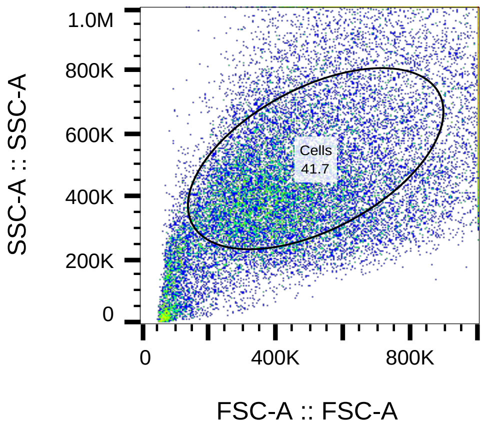


Fragment 6 -EdU_Data Source - 1.fcs
Ungated
29998

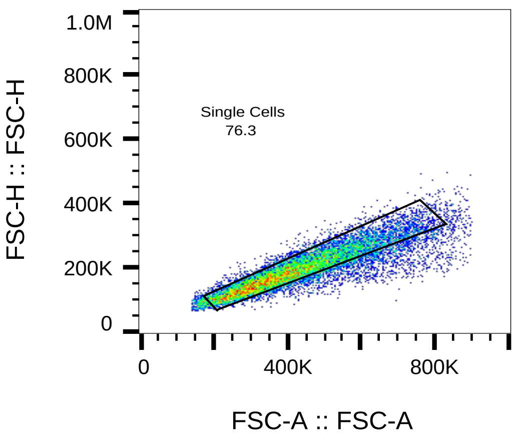


Fragment 6 -EdU_Data Source - 1.fcs
Cells
12497

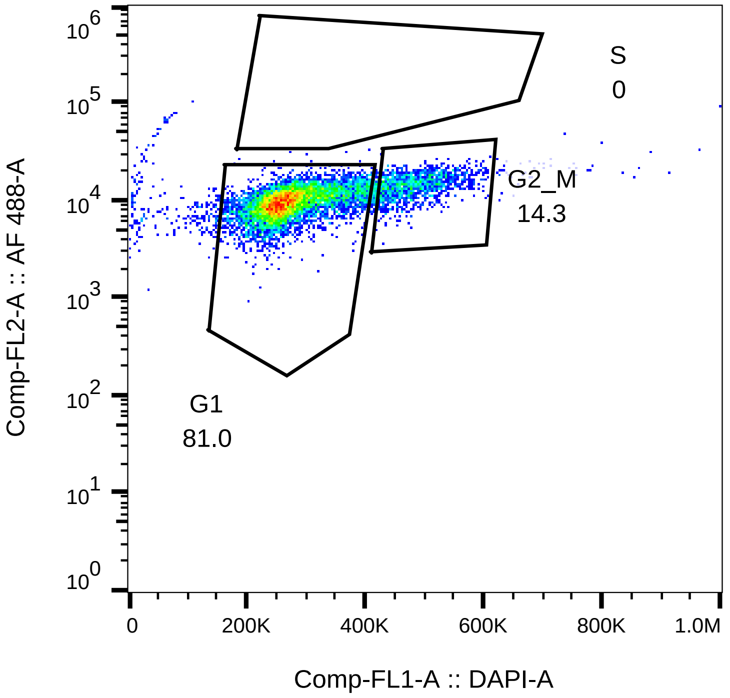


Fragment 6 -EdU_Data Source - 1.fcs
Single Cells
9530

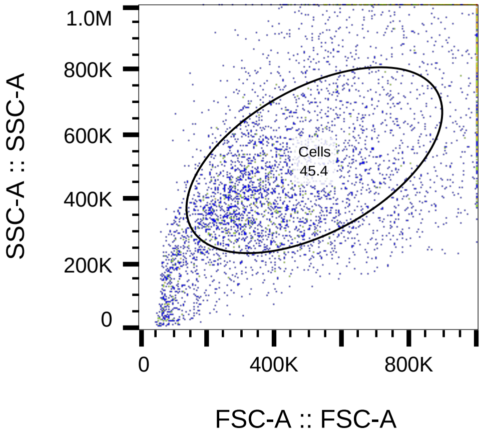


Unstained control -EdU -488 -DAPI_FSC 3 BSC
Ungated
5564

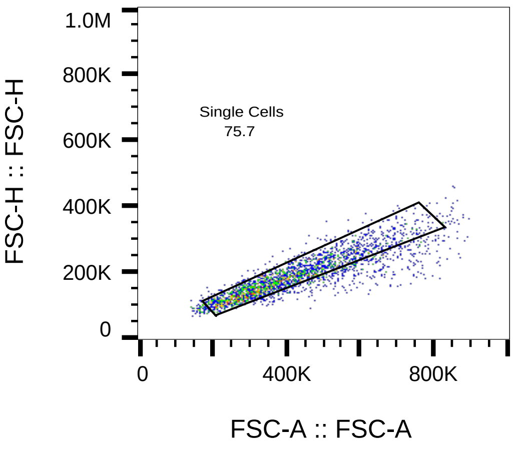


Unstained control -EdU -488 -DAPI_FSC
Cells
2527

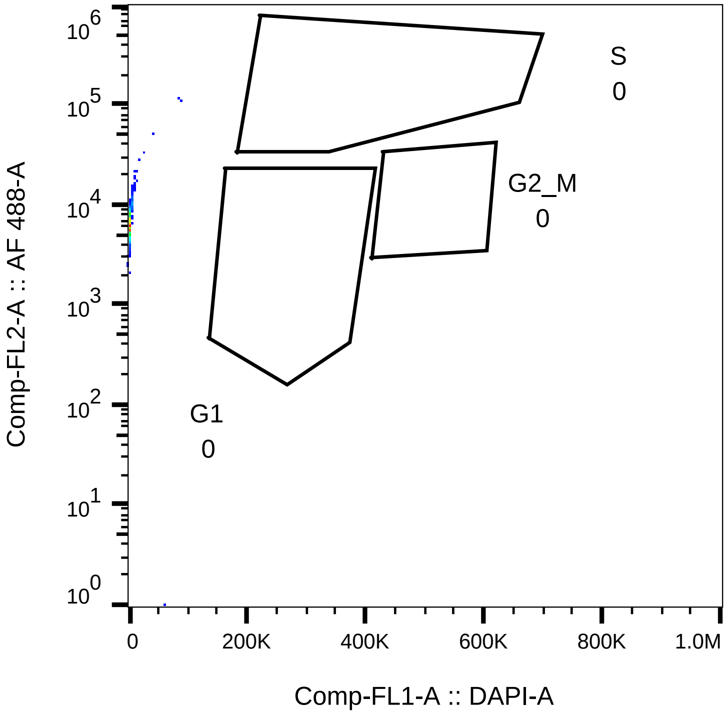


Unstained control -EdU -488 -DAPI_FSC 3 BSC 30%_Data Source - 1.f
Single Cells
1912
