# Supplementary material for: CHD8 interacts with BCL11A to induce oncogenic transcription in triple negative breast cancer
Source: EMBO J. 2025 May 6;44(12):3448–67. doi: 10.1038/s44318-025-00447-8 (PMC12170886; doi:10.1038/s44318-025-00447-8)
Supplement: Supplementary file 33 — Source data Fig. 5 [file 44318_2025_447_MOESM33_ESM.zip › Figure 5/Figure 5D/Replicate FC plots/20241219_ASEdU003.pdf]

Paper labelling- treatment DMSO

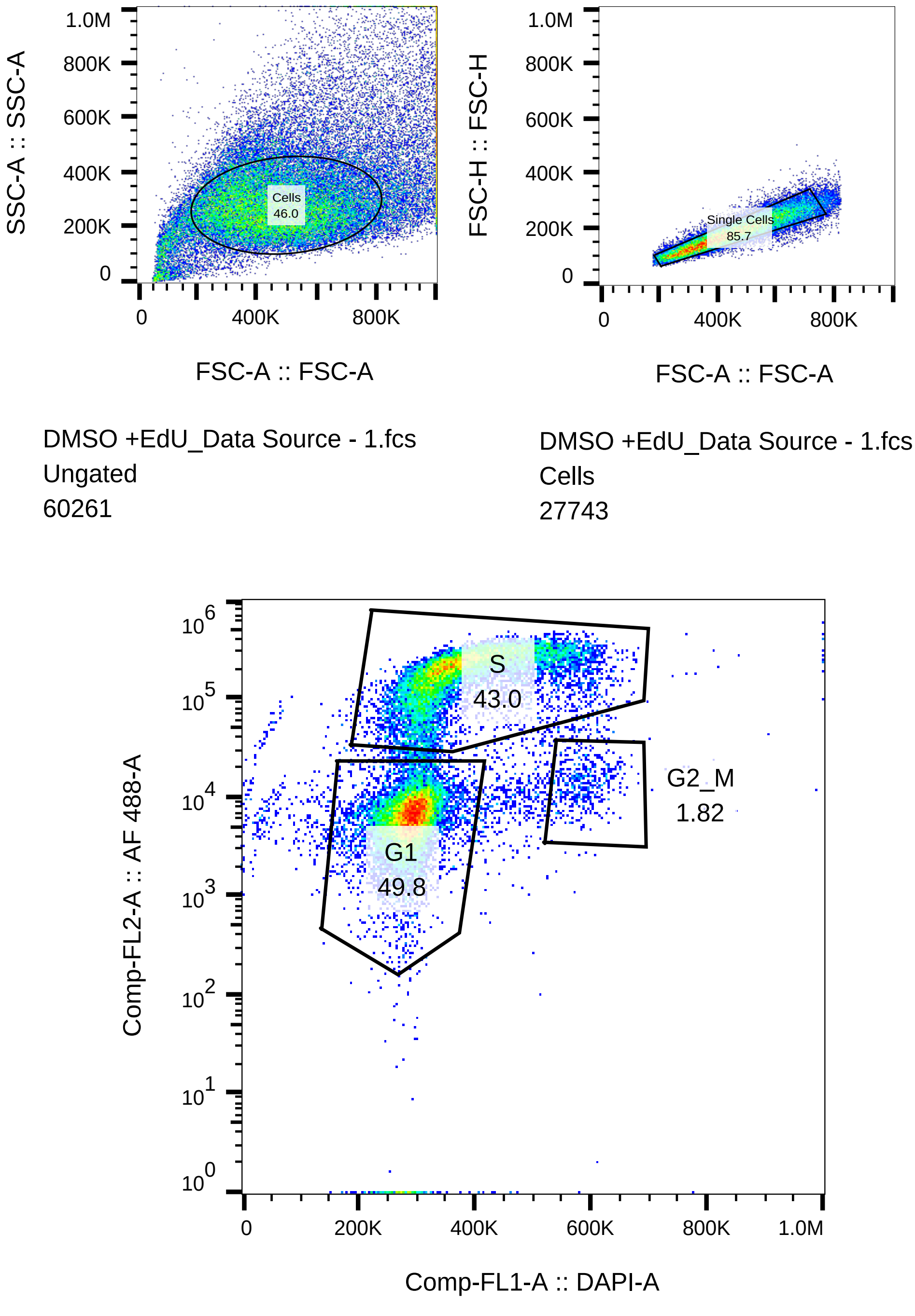

DMSO +EdU_Data Source - 1.fcs
Ungated
60261

DMSO +EdU_Data Source - 1.fcs
Cells
27743

DMSO +EdU_Data Source - 1.fcs
Single Cells
23785

Paper labelling- treatment DMSO- EdU negative control

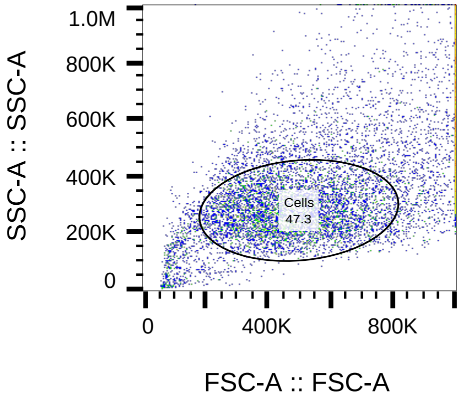

DMSO -EdU_Data Source - 1.fcs
Ungated
10755

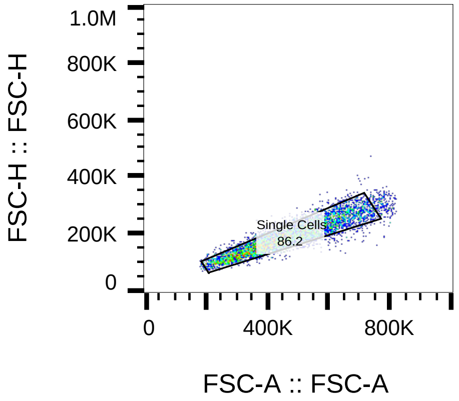

DMSO -EdU_Data Source - 1.fcs
Cells
5083

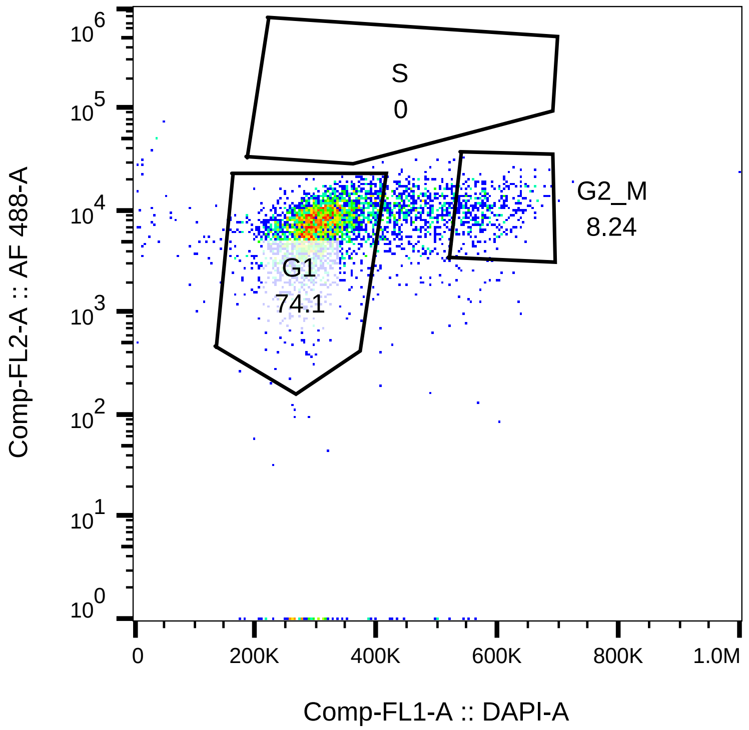

DMSO -EdU_Data Source - 1.fcs
Single Cells
4381

Paper labelling- treatment fragment 1- EdU negative control

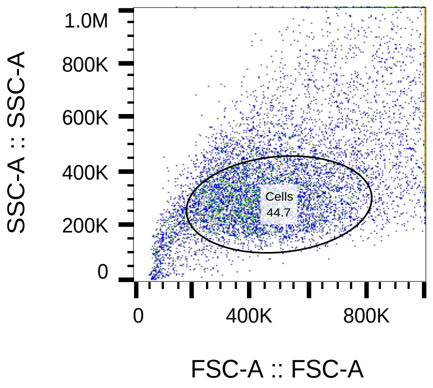

Fragment 1 -EdU_Data Source - 1.fcs
Ungated
10816

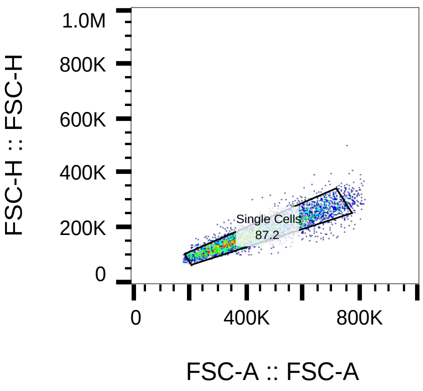

Fragment 1 -EdU_Data Source - 1.fcs
Cells
4836

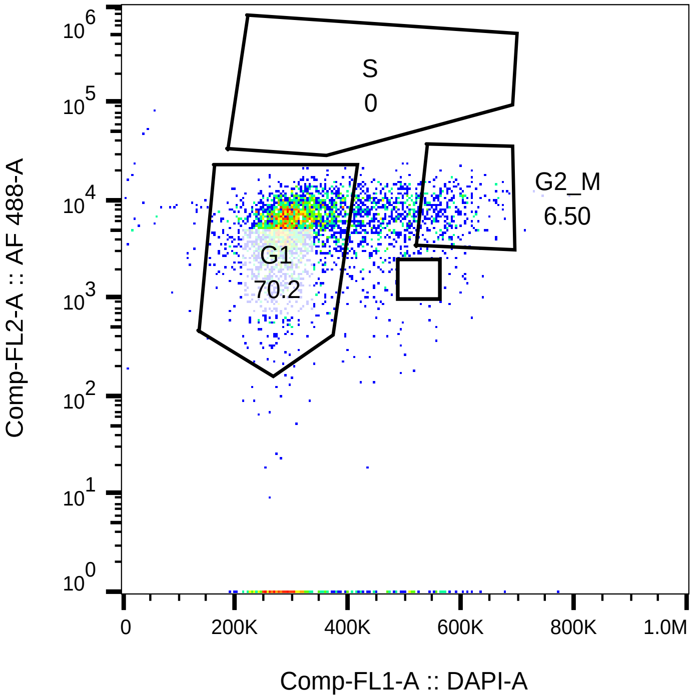

Fragment 1 -EdU_Data Source - 1.fcs
Single Cells
4217

Paper labelling- treatment fragment 1

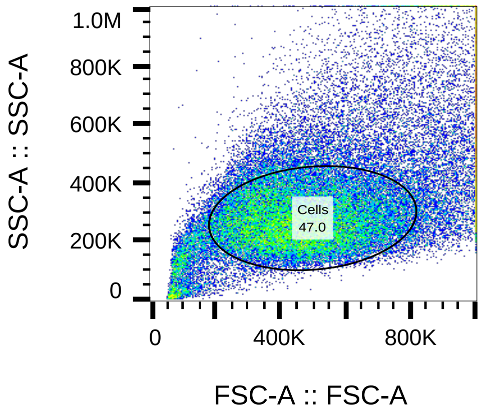

Fragment 1+EdU_Data Source - 1.fcs
Ungated
61996

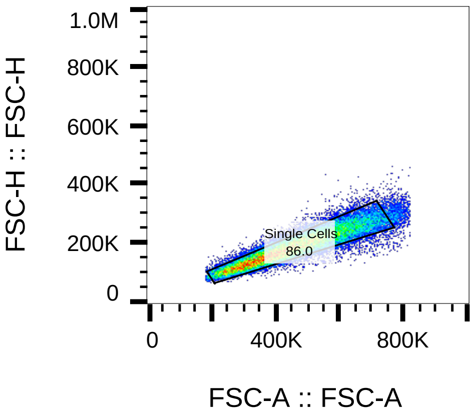

Fragment 1+EdU_Data Source - 1.fcs
Cells
29115

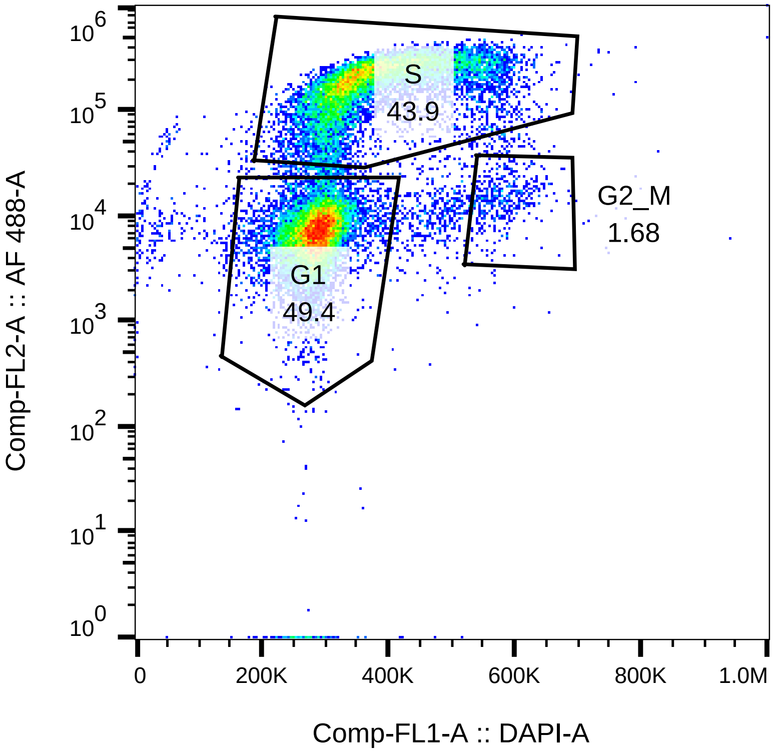

Fragment 1+EdU_Data Source - 1.fcs
Single Cells
25043

Paper labelling- treatment fragment 3

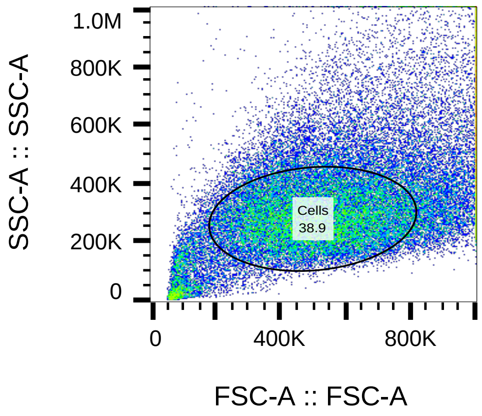

Fragment 4 +EdU_Data Source - 1.fcs
Ungated
61774

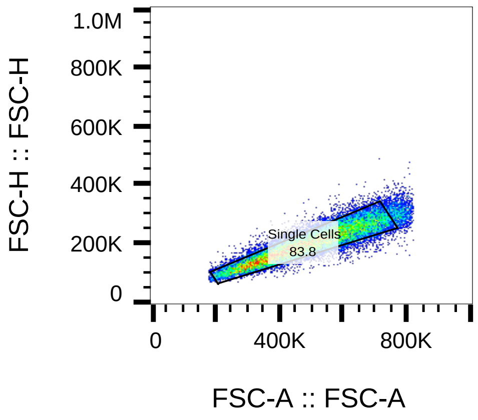

Fragment 4 +EdU_Data Source - 1.fcs
Cells
24057

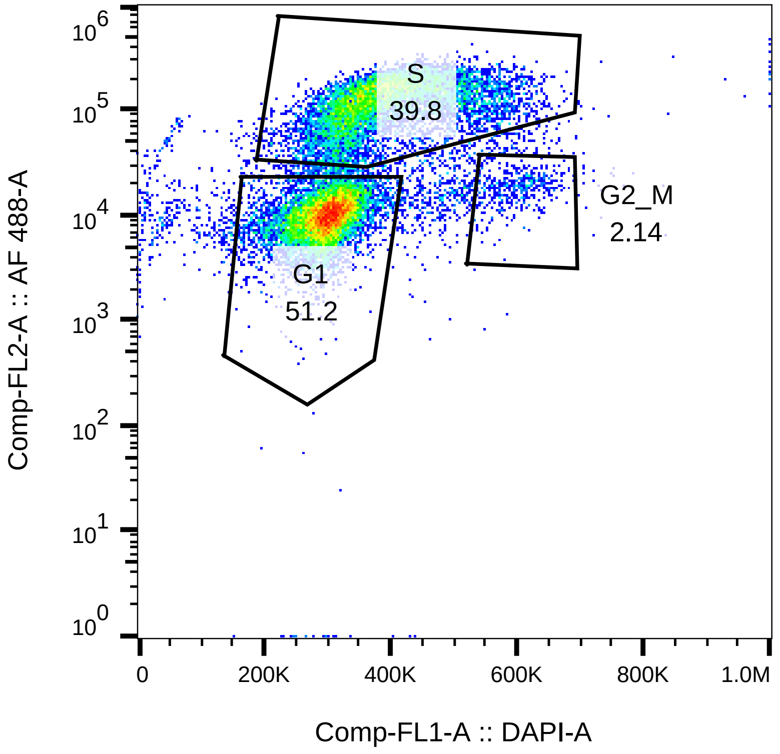

Fragment 4 +EdU_Data Source - 1.fcs
Single Cells
20169

Paper labelling- treatment fragment 3- EdU negative control

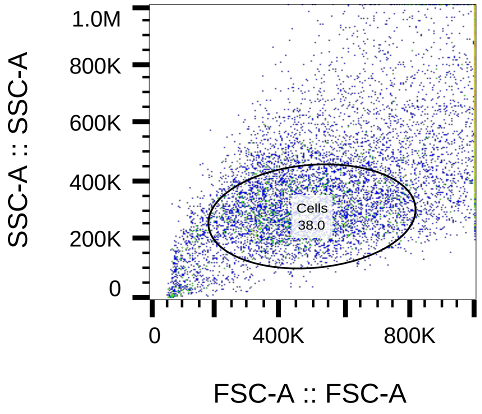

Fragment 4 -EdU_Data Source - 1.fcs
Ungated
10430

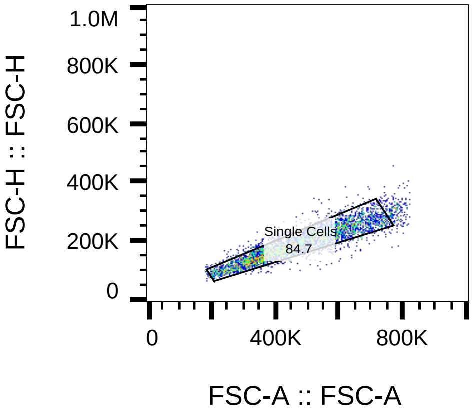

Fragment 4 -EdU_Data Source - 1.fcs
Cells
3967

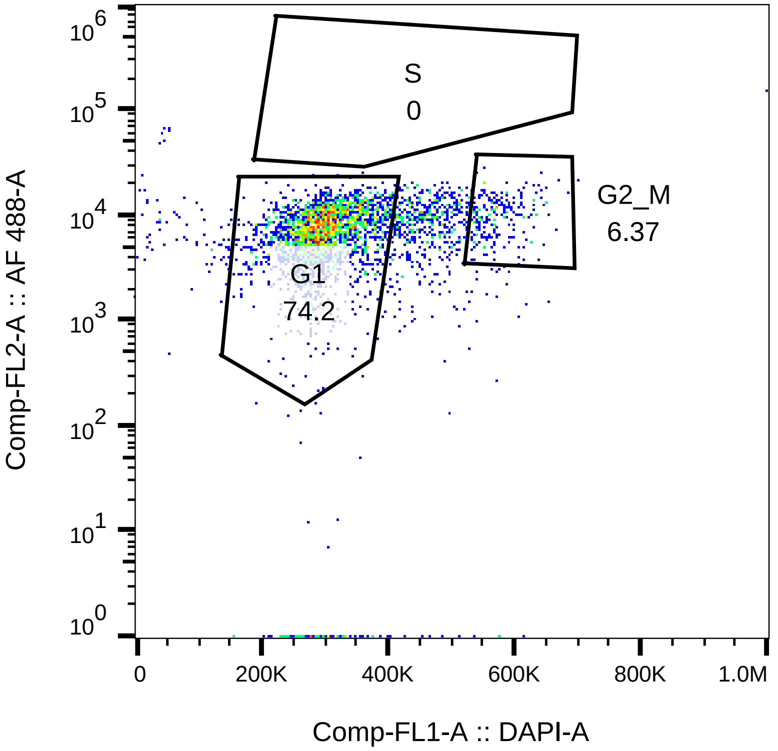

Fragment 4 -EdU_Data Source - 1.fcs
Single Cells
3360

Paper labelling- treatment fragment 5

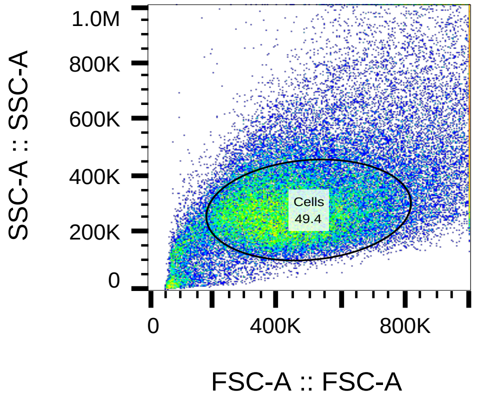

Fragment 6 +EdU_Data Source - 1.fcs
Ungated
61685

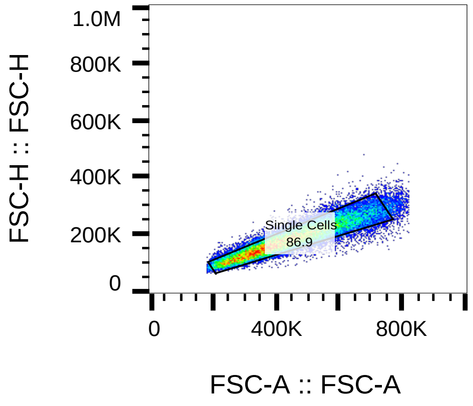

Fragment 6 +EdU_Data Source - 1.fcs
Cells
30500

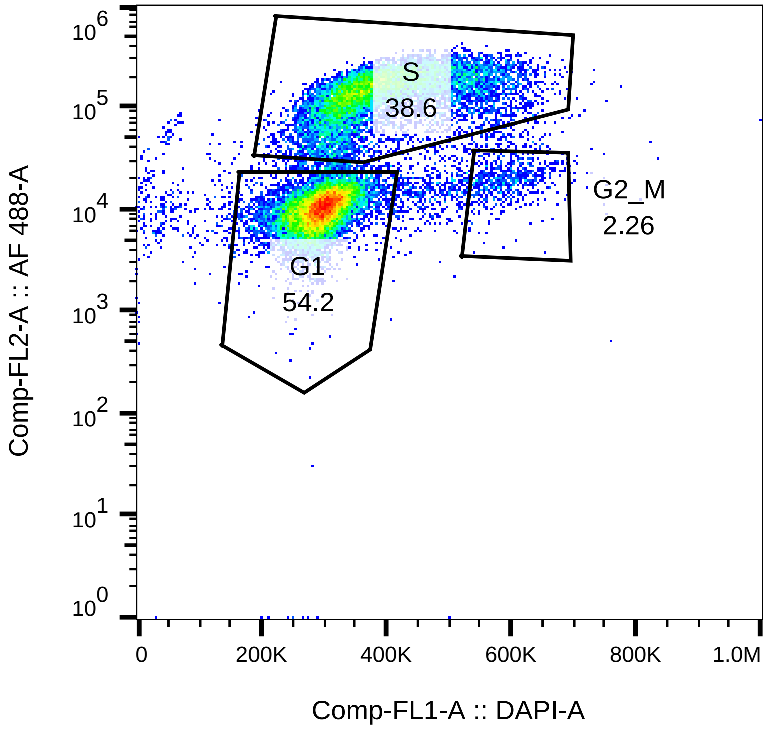

Fragment 6 +EdU_Data Source - 1.fcs
Single Cells
26516

Paper labelling- treatment fragment 5 EdU negative control

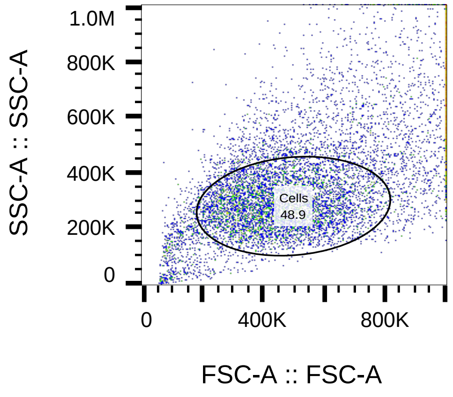

Fragment 6 -EdU_Data Source - 1.fcs
Ungated
10471

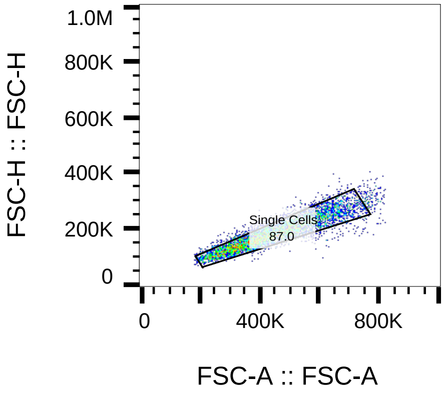

Fragment 6 -EdU_Data Source - 1.fcs
Cells
5123

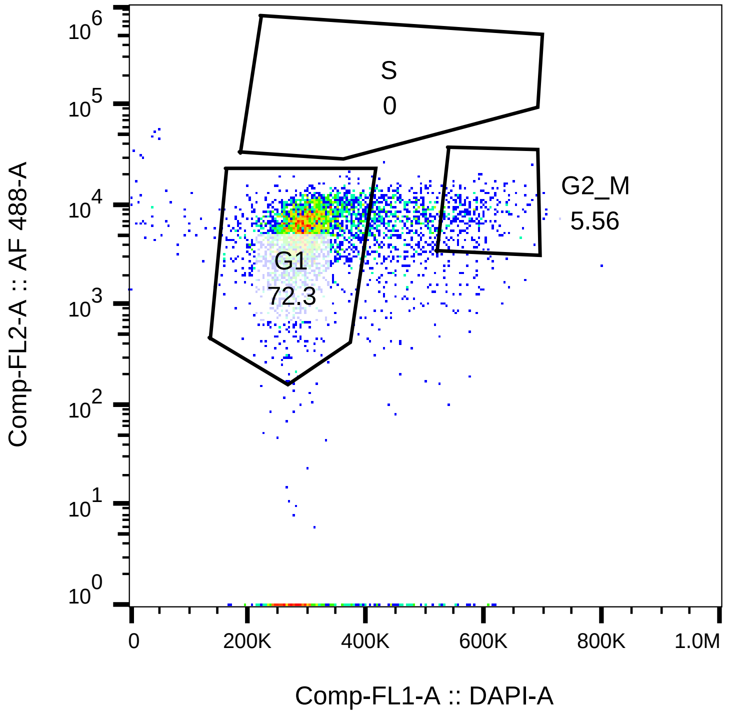

Fragment 6 -EdU_Data Source - 1.fcs
Single Cells
4459

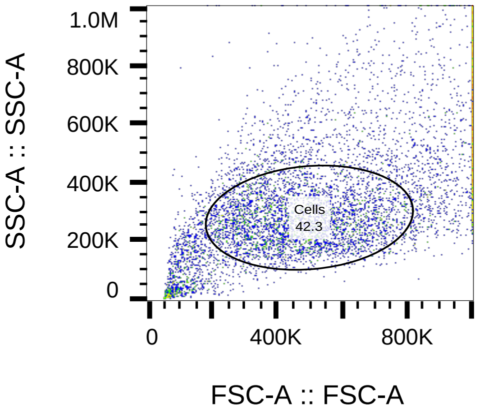

Single colour control +EdU +488 -DAPI - 1_
Ungated
8170

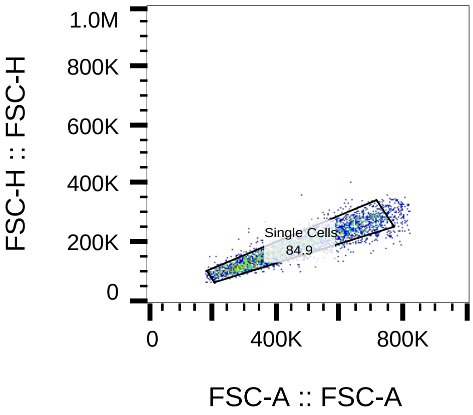

Single colour control +EdU +488 -DAPI -
Cells
3459

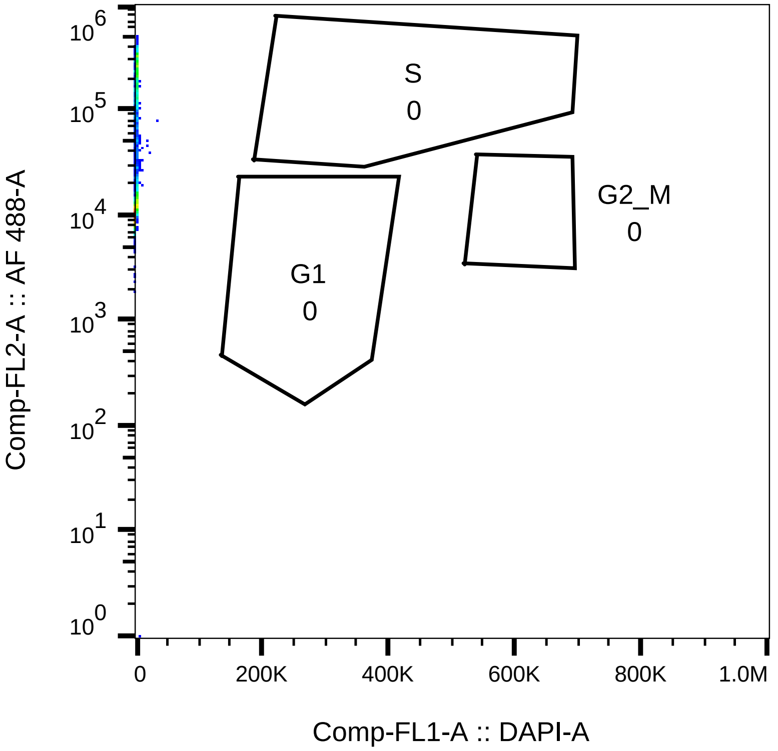

Single colour control +EdU +488 -DAPI - 1_Data Source - 1.fcs
Single Cells
2938

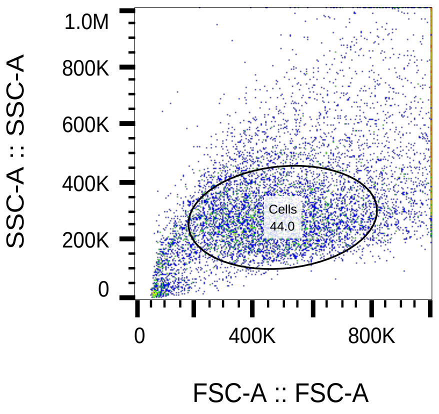

Single colour control +EdU -488 +DAPI _Da
Ungated
10385

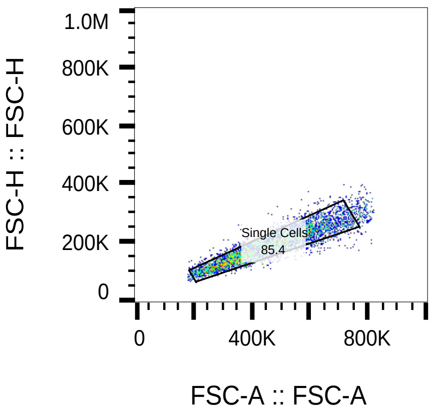

Single colour control +EdU -488 +DAPI _Data Sou
Cells
4565

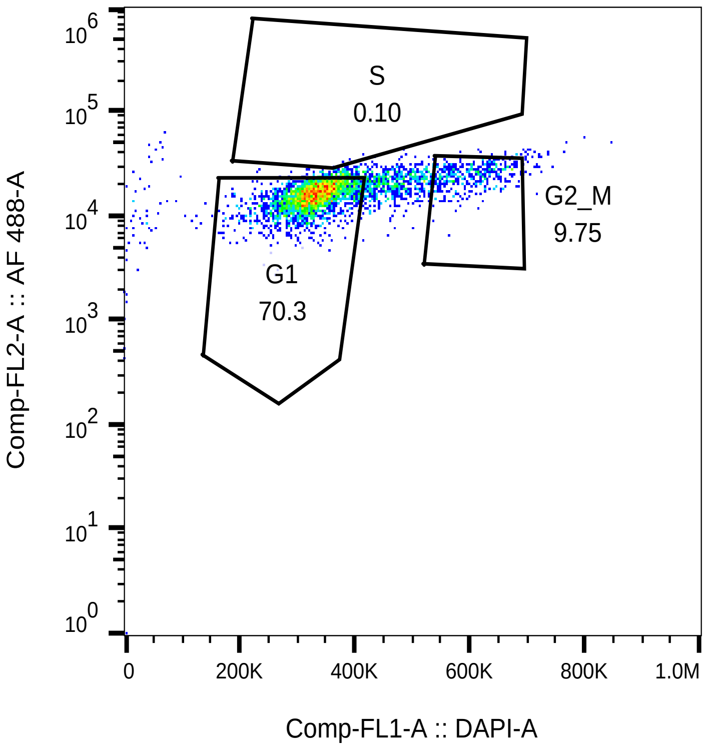

Single colour control +EdU -488 +DAPI _Data Source - 1.fcs
Single Cells
3897

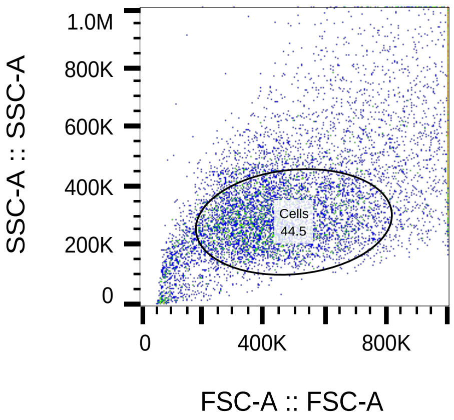

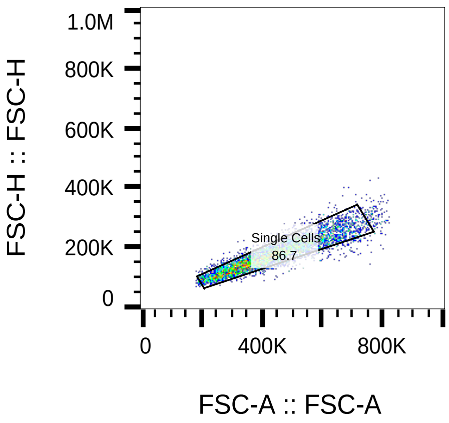

Unstained control -EdU -488 -DAPI_Data S
Ungated
10362

Unstained control -EdU -488 -DAPI_Data S
Cells
4611

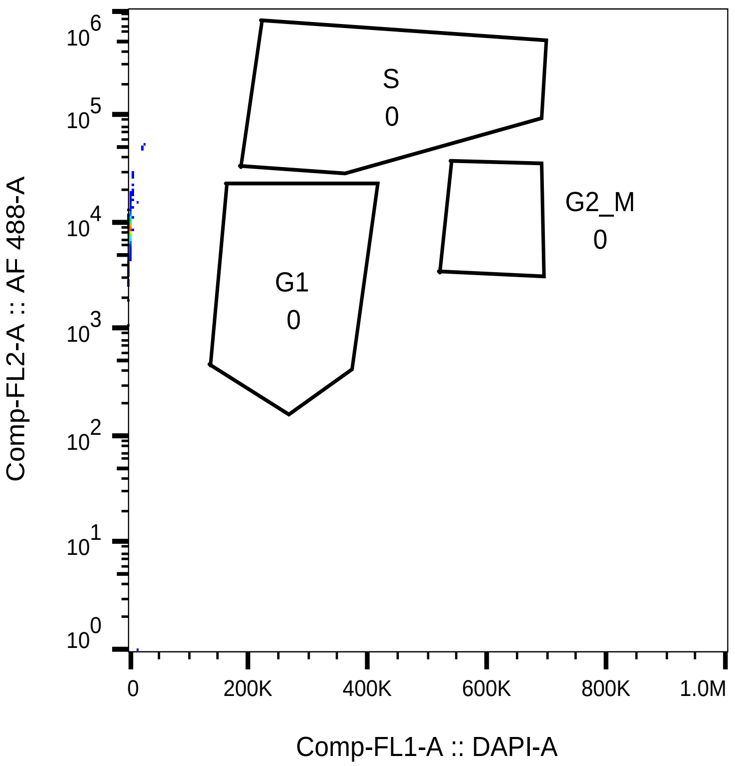

Unstained control -EdU -488 -DAPI_Data Source - 1.fcs
Single Cells
3998
