# Supplementary material for: CHD8 interacts with BCL11A to induce oncogenic transcription in triple negative breast cancer
Source: EMBO J. 2025 May 6;44(12):3448–67. doi: 10.1038/s44318-025-00447-8 (PMC12170886; doi:10.1038/s44318-025-00447-8)
Supplement: Supplementary file 33 — Source data Fig. 5 [file 44318_2025_447_MOESM33_ESM.zip › Figure 5/Figure 5D/Replicate FC plots/20241212_4T1_EdU_Exp002.pdf]

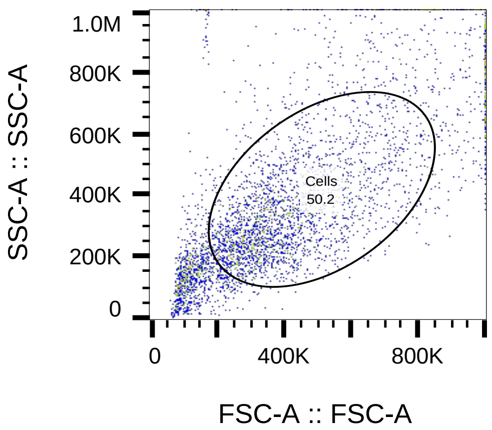


+EdU +488 -DAPI  (FSC gain 2)_Data Sou
Ungated
4694

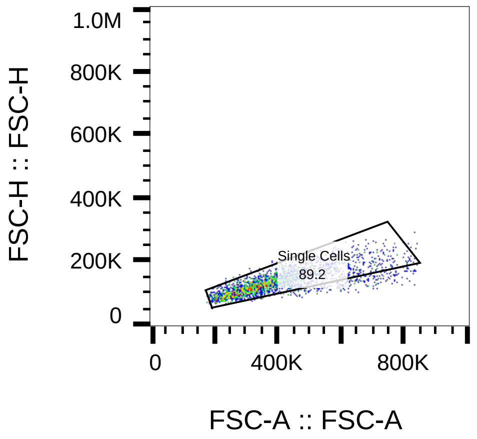


+EdU +488 -DAPI  (FSC gain 2)_Data
Cells
2357

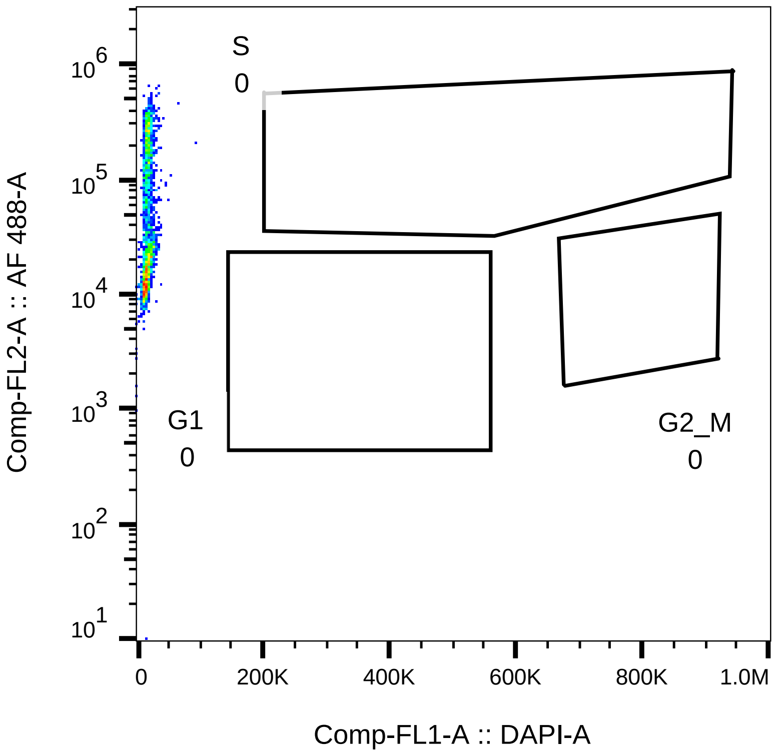


+EdU +488 -DAPI  (FSC gain 2)_Data Source - 1.fcs
Single Cells
2102

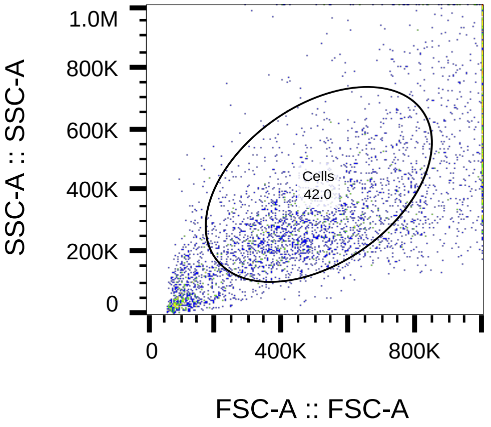


+EdU +488 -DAPI  (FSC gain 3)_Data Sou
Ungated
5060

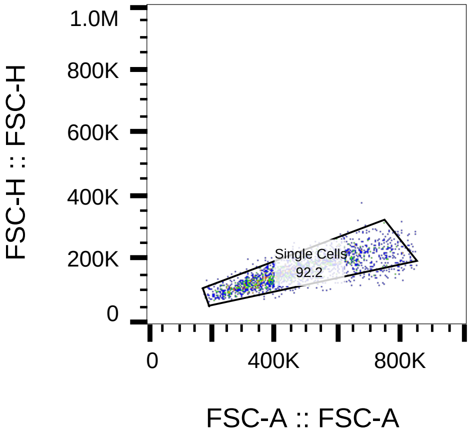


+EdU +488 -DAPI  (FSC gain 3)_Data
Cells
2124

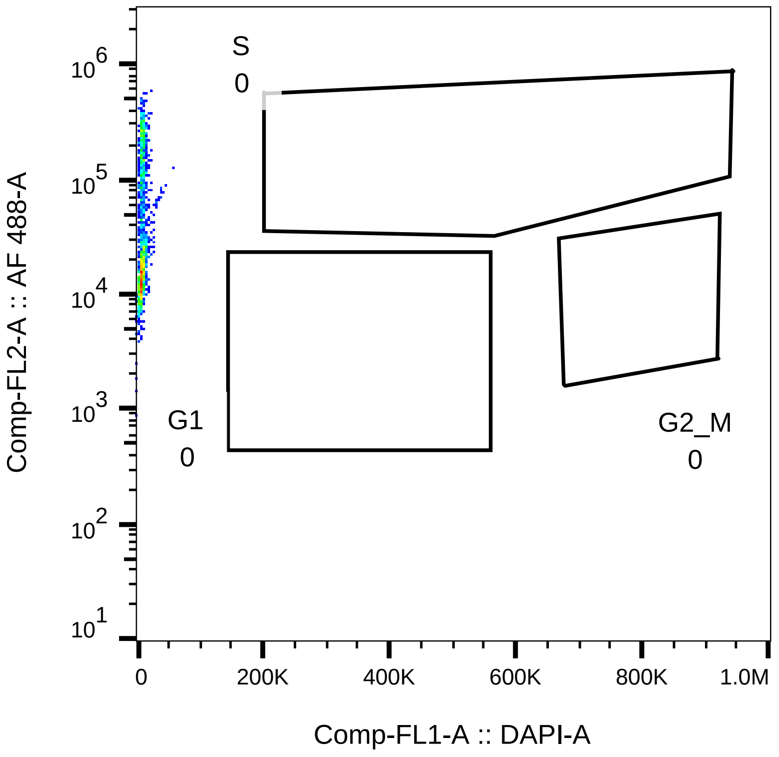


+EdU +488 -DAPI  (FSC gain 3)_Data Source - 1.fcs
Single Cells
1959

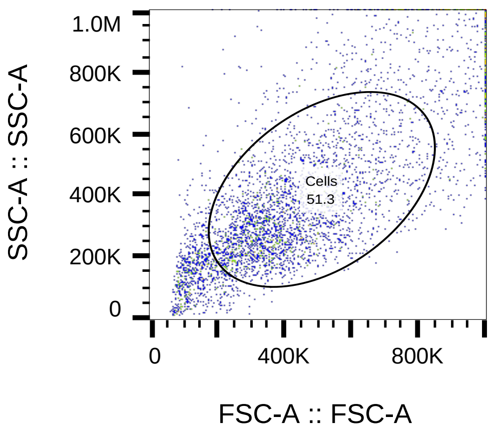


+EdU -488 +DAPI_Data Source - 1.fcs
Ungated
5015

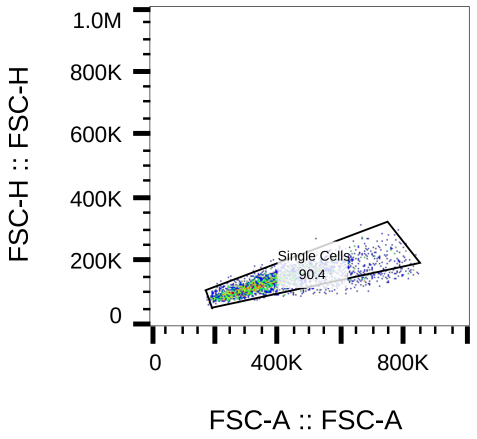


+EdU -488 +DAPI_Data Source - 1.fcs
Cells
2572

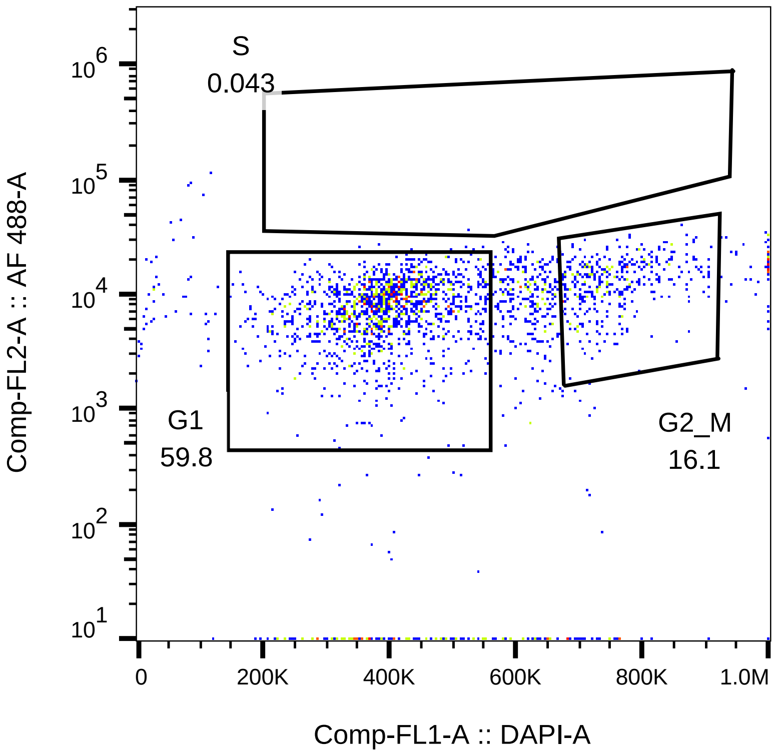


+EdU -488 +DAPI_Data Source - 1.fcs
Single Cells
2325

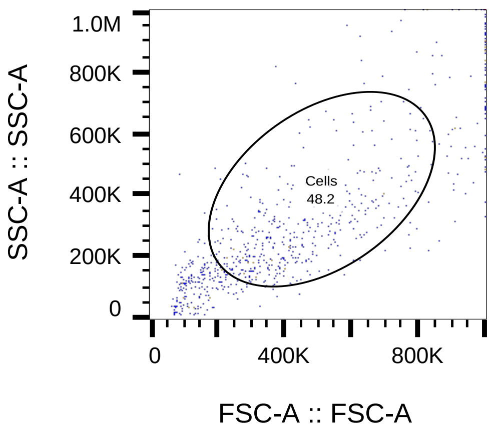


-EdU +488 -DAPI_Data Source - 1.fcs
Ungated
792

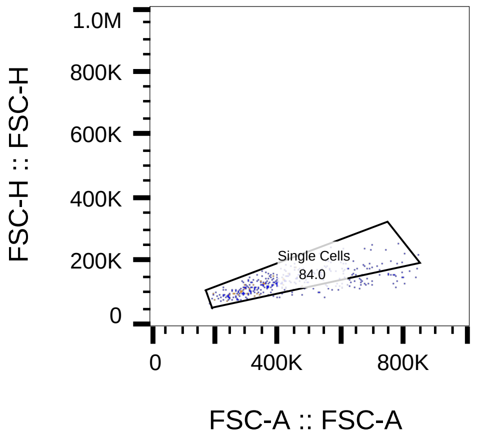


-EdU +488 -DAPI_Data Source - 1.fcs
Cells
382

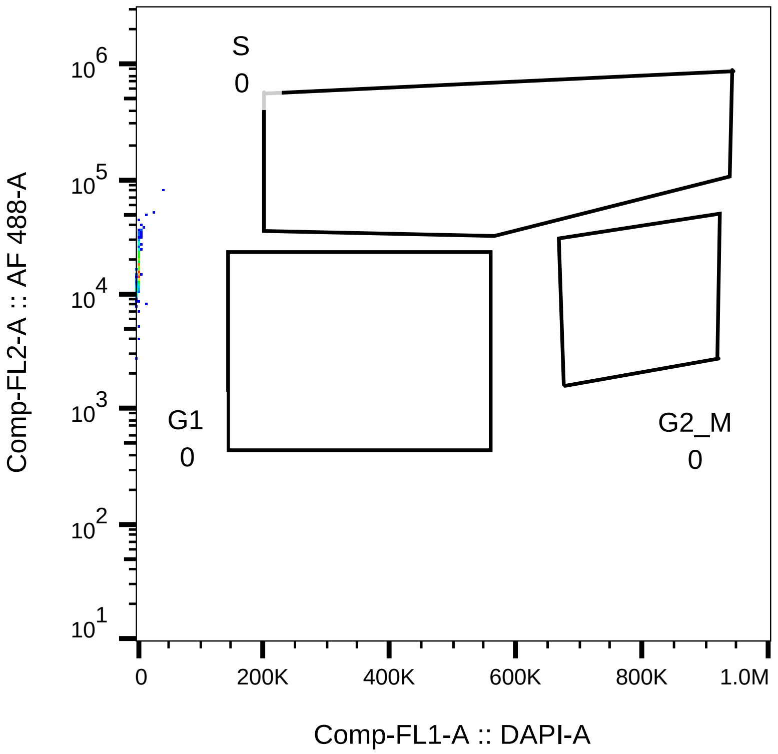


-EdU +488 -DAPI_Data Source - 1.fcs
Single Cells
321

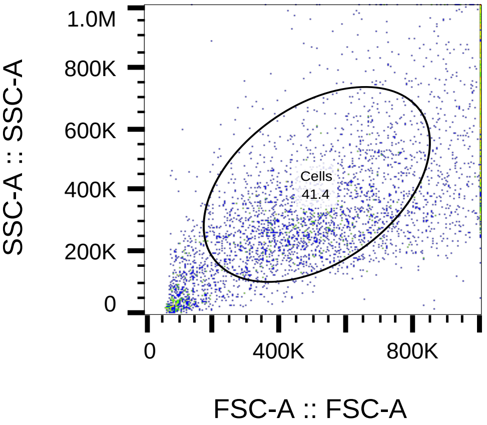


-EdU -488 +DAPI_Data Source - 1.fcs
Ungated
5065

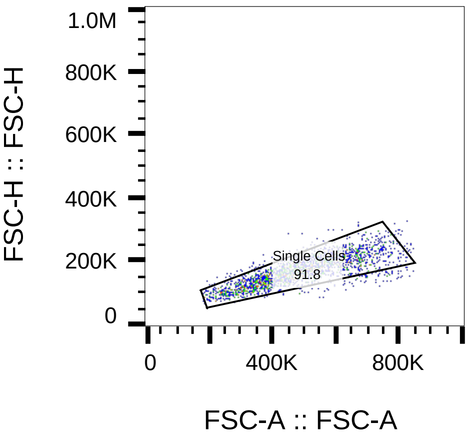


-EdU -488 +DAPI_Data Source - 1.fcs
Cells
2099

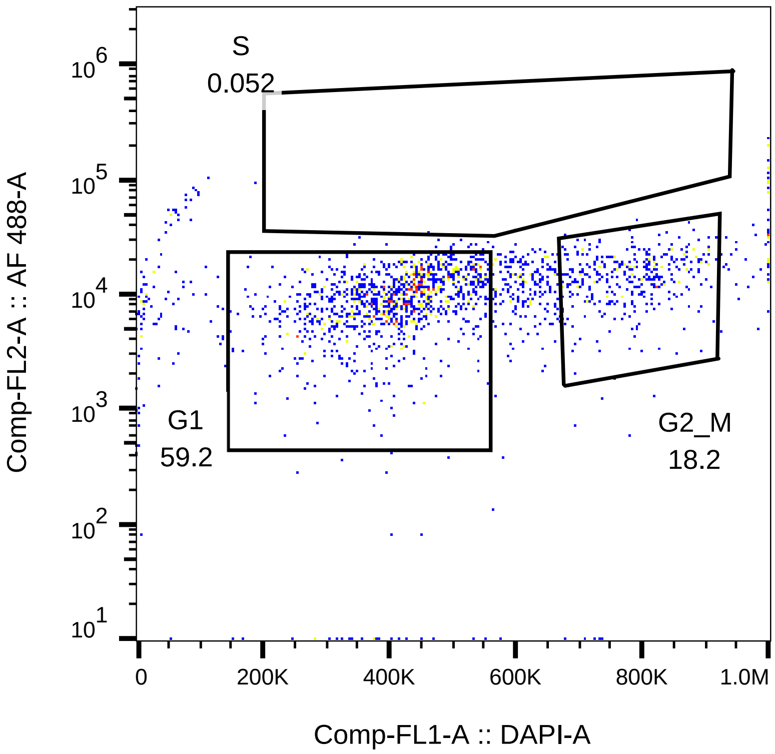


-EdU -488 +DAPI_Data Source - 1.fcs
Single Cells
1927

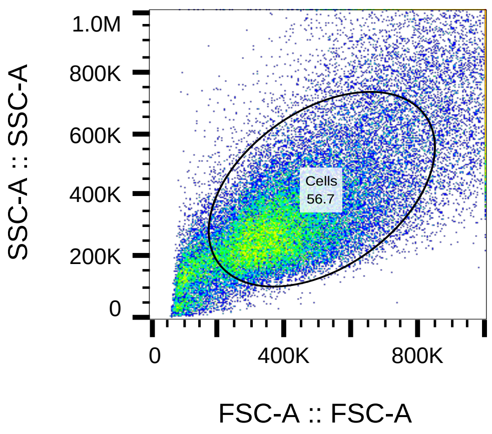


DMSO +EdU_Data Source - 1.fcs
Ungated
50124

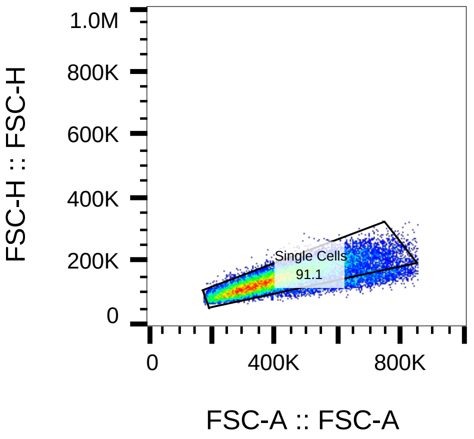


DMSO +EdU_Data Source - 1.fcs
Cells
28436

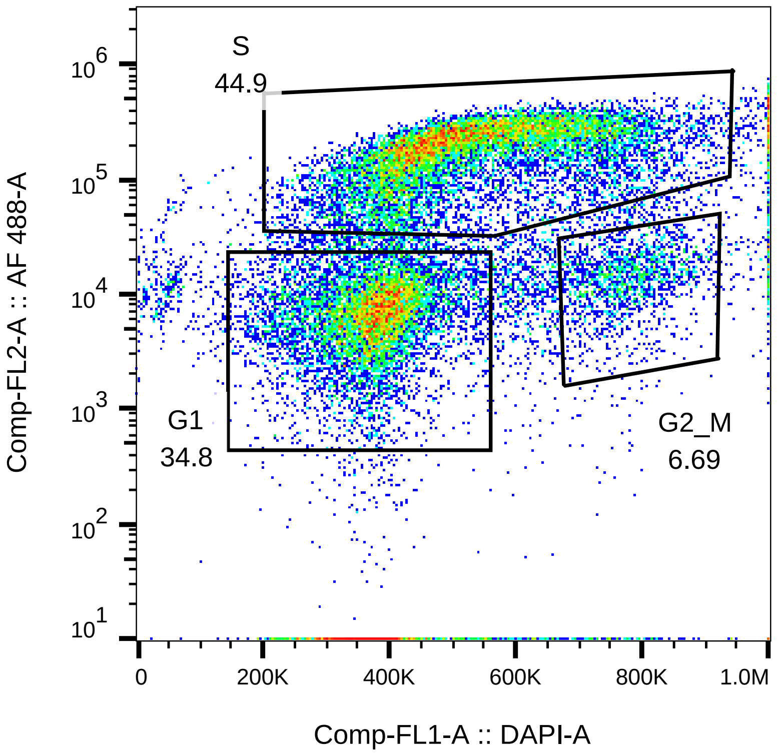


DMSO +EdU_Data Source - 1.fcs
Single Cells
25905

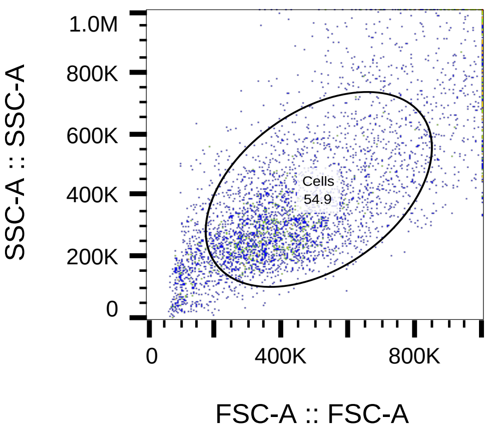


DMSO -EdU_Data Source - 1.fcs
Ungated
5196

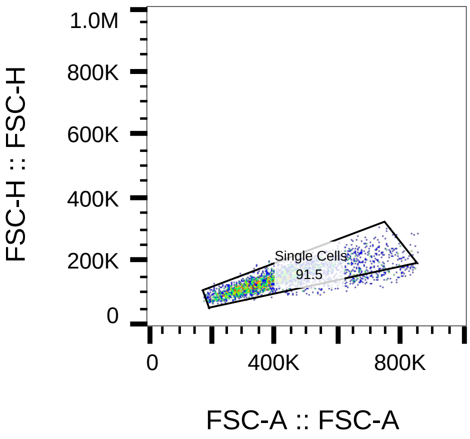


DMSO -EdU_Data Source - 1.fcs
Cells
2854

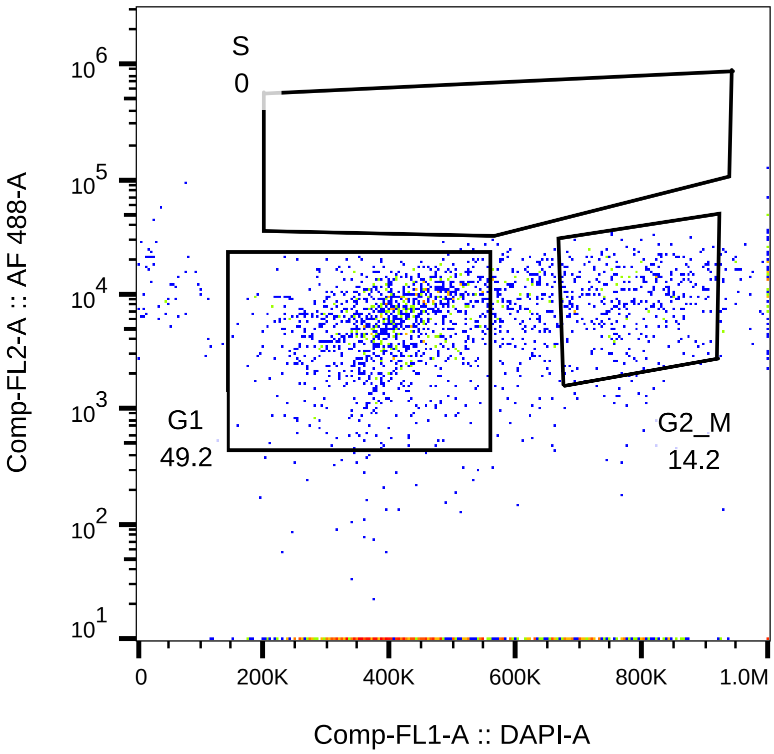


DMSO -EdU_Data Source - 1.fcs
Single Cells
2612

Paper labelling- treatment Fragment 1

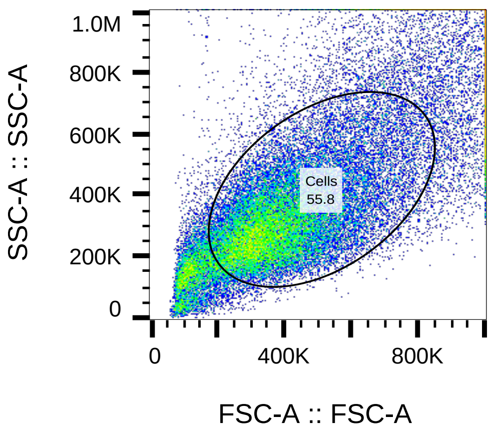


Fragment 1 +EdU_Data Source - 1.fcs
Ungated
50623

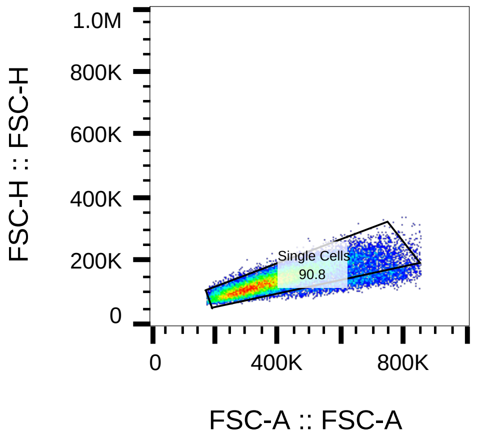


Fragment 1 +EdU_Data Source - 1.fcs
Cells
28258

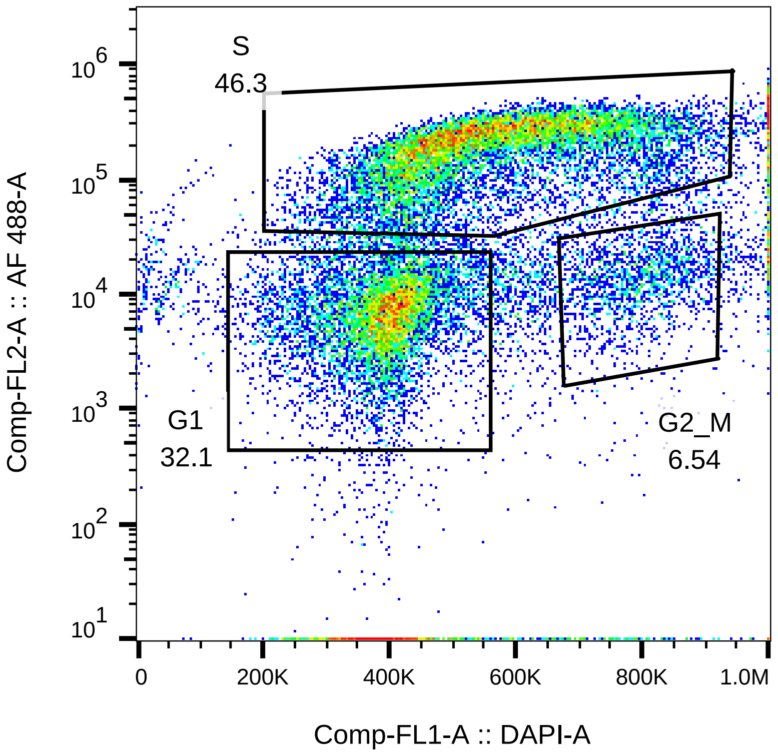


Fragment 1 +EdU_Data Source - 1.fcs
Single Cells
25646

Paper labelling- treatment Fragment 1, EdU-ve control

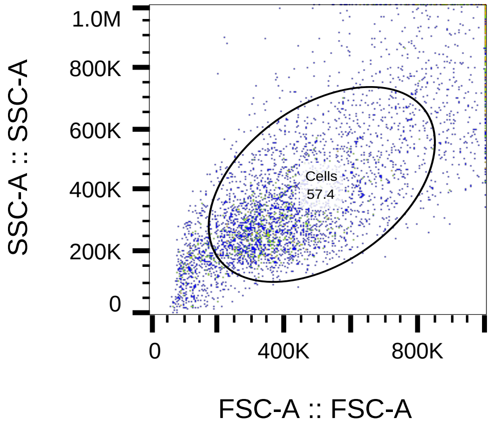


Fragment 1 -EdU_Data Source - 1.fcs
Ungated
5164

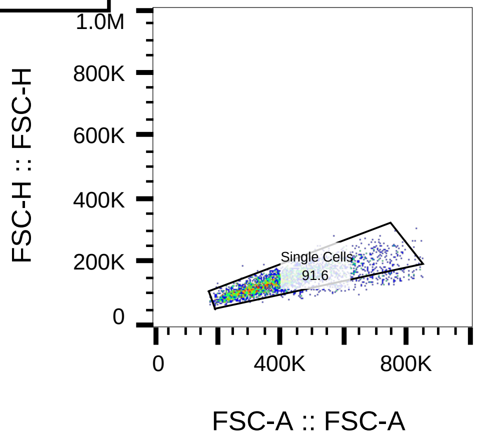


Fragment 1 -EdU_Data Source - 1.fcs
Cells
2966

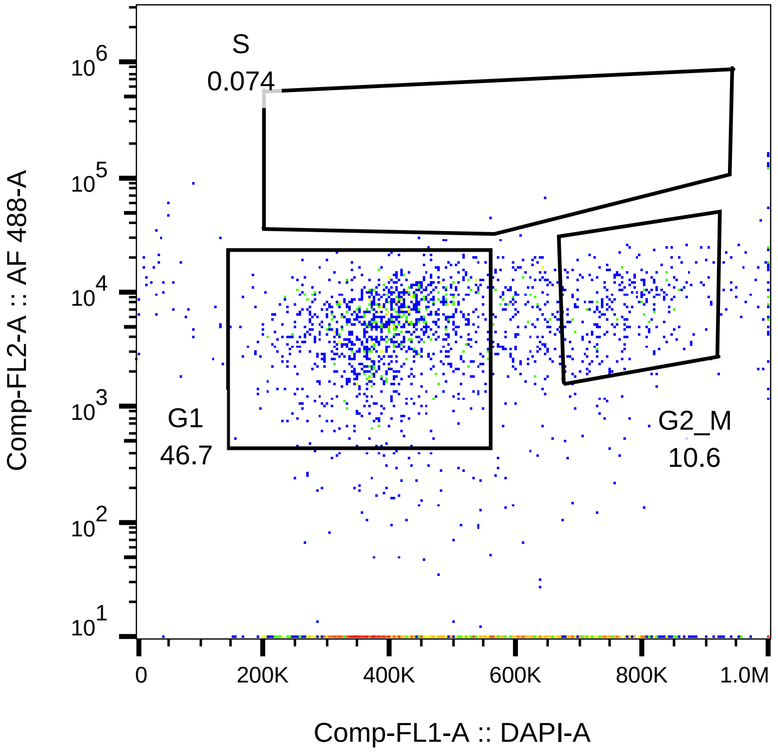


Fragment 1 -EdU_Data Source - 1.fcs
Single Cells
2718

Paper labelling- treatment Fragment 3

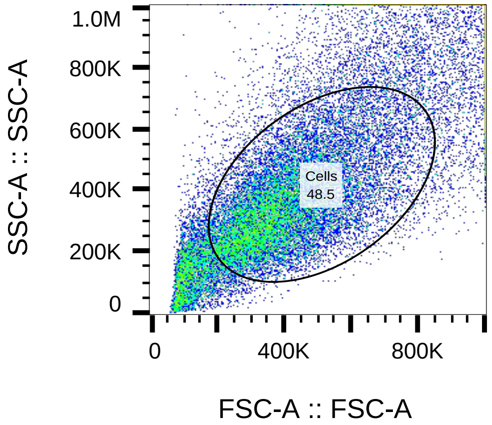


Fragment 4 +EdU_Data Source - 1.fcs
Ungated
37026

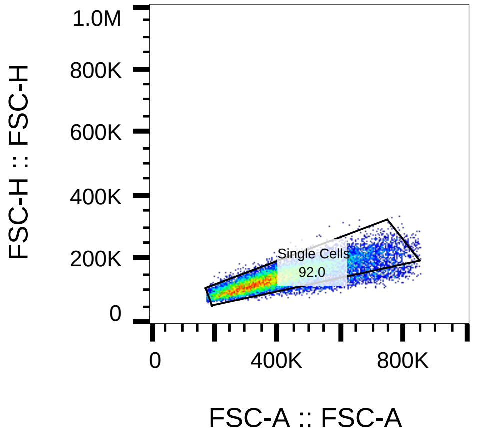


Fragment 4 +EdU_Data Source - 1.fcs
Cells
17950

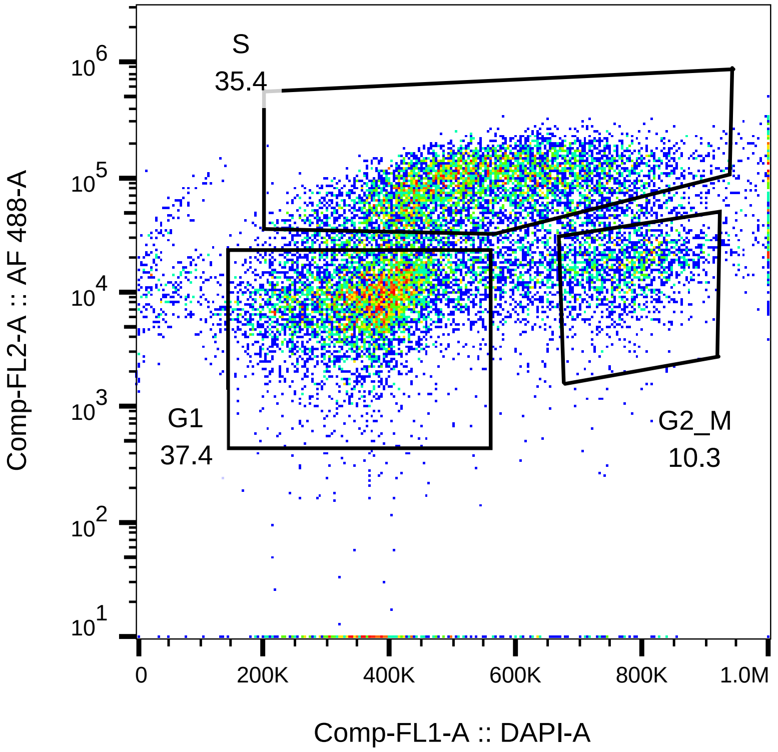


Fragment 4 +EdU_Data Source - 1.fcs
Single Cells
16521

Paper labelling- treatment Fragment 3 , EdU-ve control

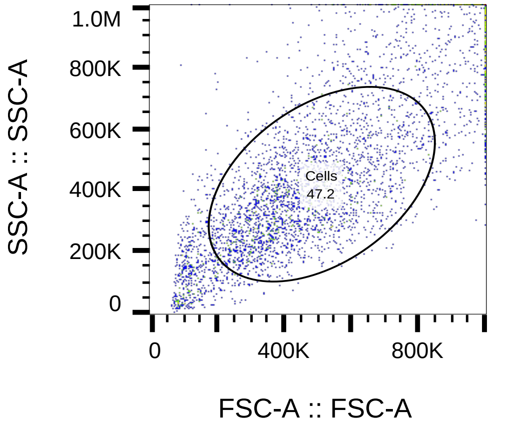


Fragment 4 -EdU_Data Source - 1.fcs
Ungated
5082

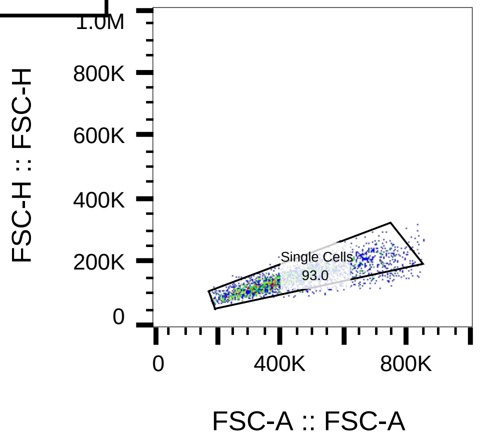


Fragment 4 -EdU_Data Source - 1.fcs
Cells
2400

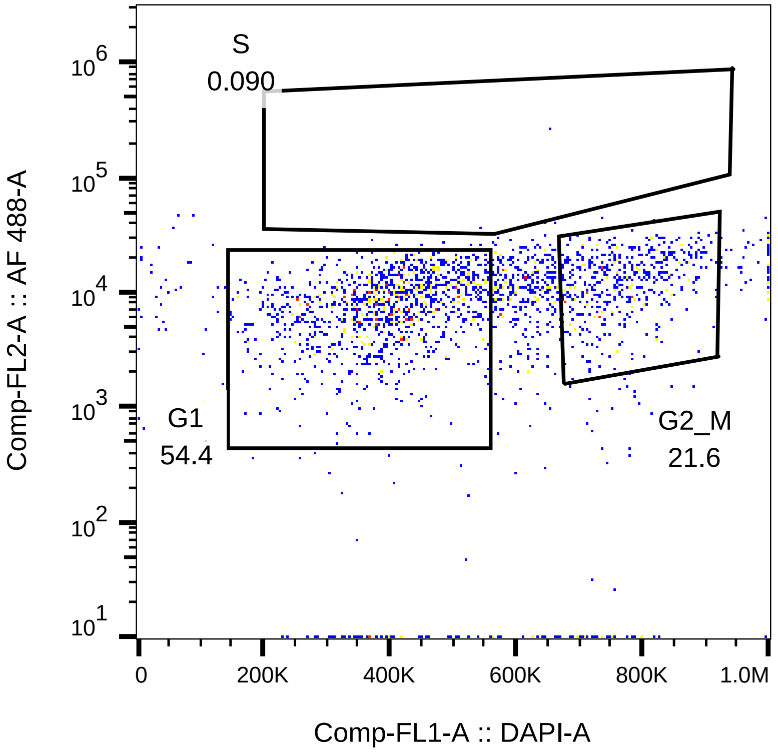


Fragment 4 -EdU_Data Source - 1.fcs
Single Cells
2231

Paper labelling- treatment Fragment 5

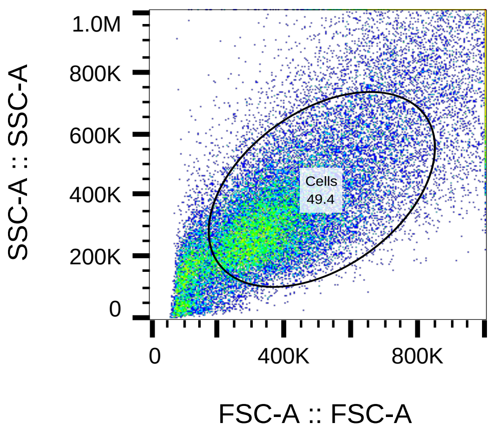


Fragment 6 +EdU_Data Source - 1.fcs
Ungated
40736

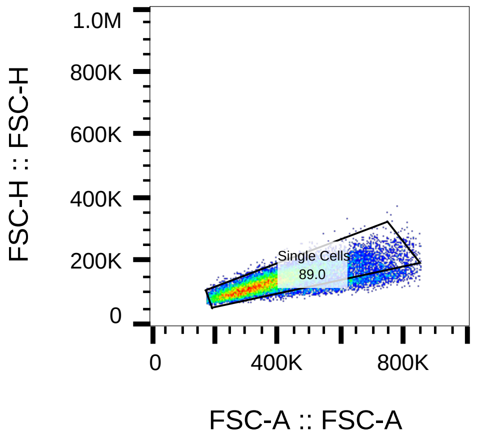


Fragment 6 +EdU_Data Source - 1.fcs
Cells
20126

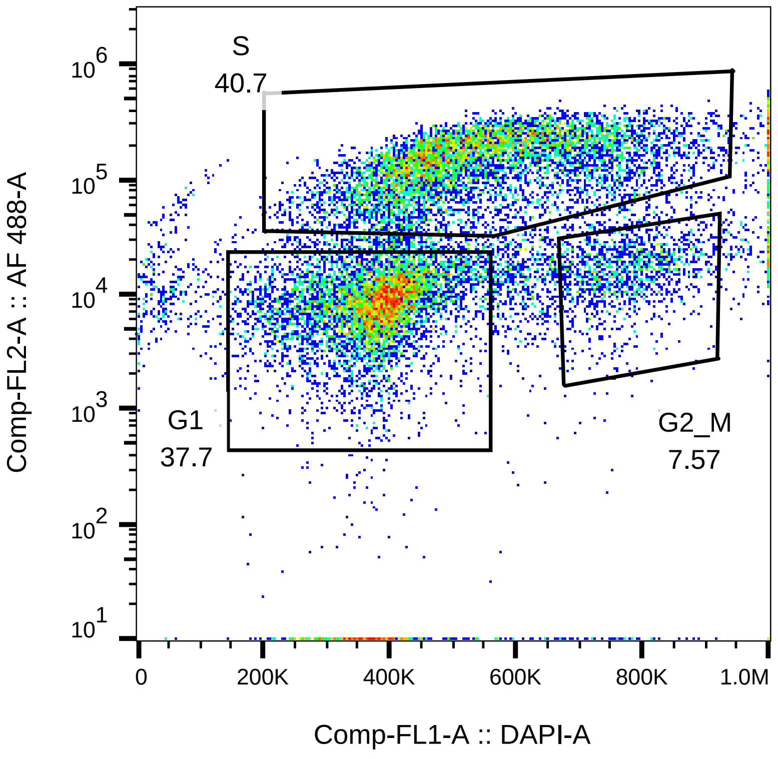


Fragment 6 +EdU_Data Source - 1.fcs
Single Cells
17908

Paper labelling- treatment Fragment 5, EdU-ve control

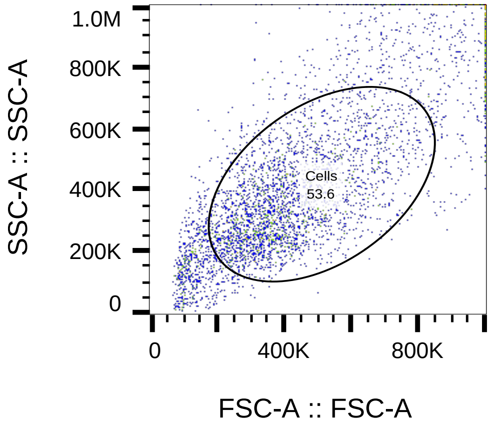


Fragment 6 -EdU_Data Source - 1.fcs
Ungated
5101

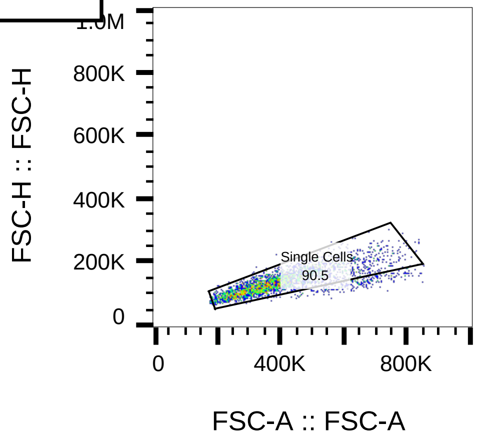


Fragment 6 -EdU_Data Source - 1.fcs
Cells
2734

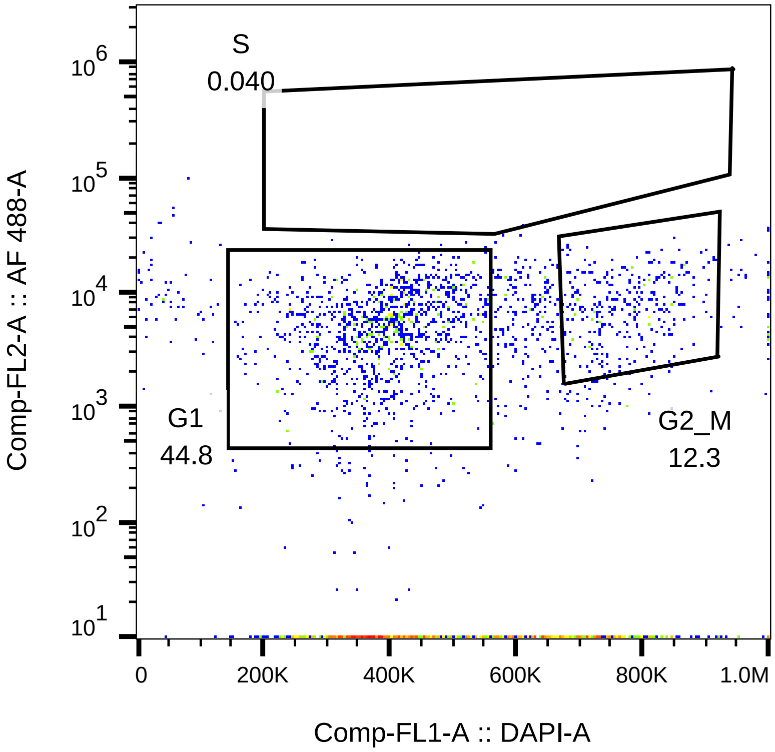


Fragment 6 -EdU_Data Source - 1.fcs
Single Cells
2473
