# Supplementary material for: CHD8 interacts with BCL11A to induce oncogenic transcription in triple negative breast cancer
Source: EMBO J. 2025 May 6;44(12):3448–67. doi: 10.1038/s44318-025-00447-8 (PMC12170886; doi:10.1038/s44318-025-00447-8)
Supplement: Supplementary file 34 — Appendix Figure Source Data [file 44318_2025_447_MOESM34_ESM.zip › Appendix_Figure_S1.zip!/Appendix Figure 1/CHD8 blot.pdf]

29-10-16

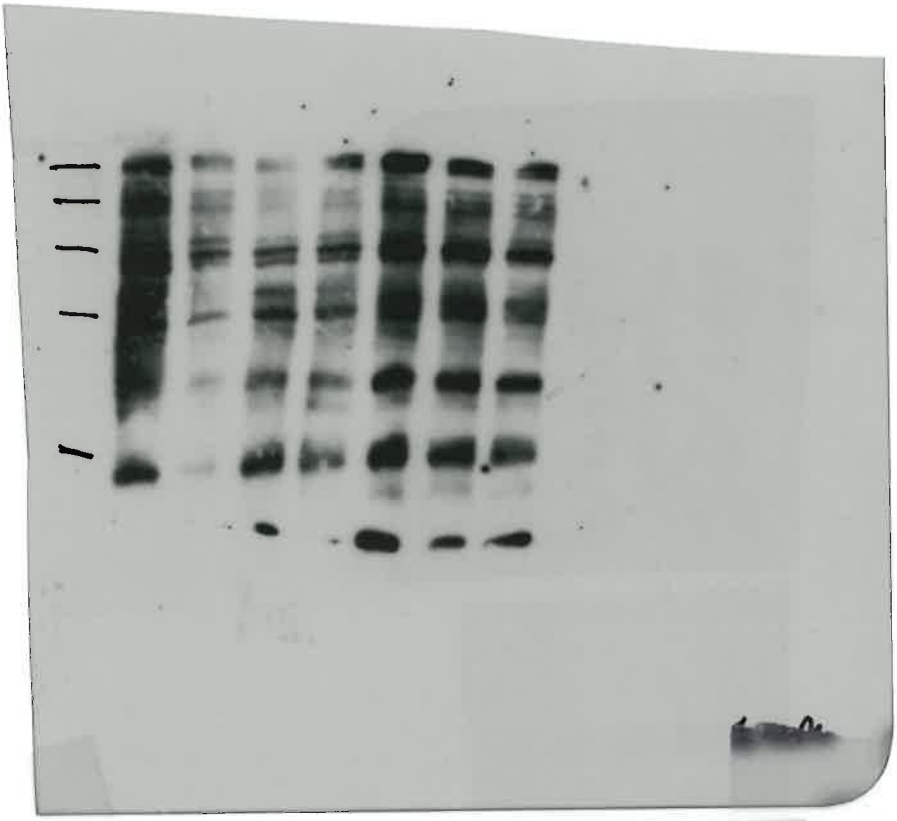

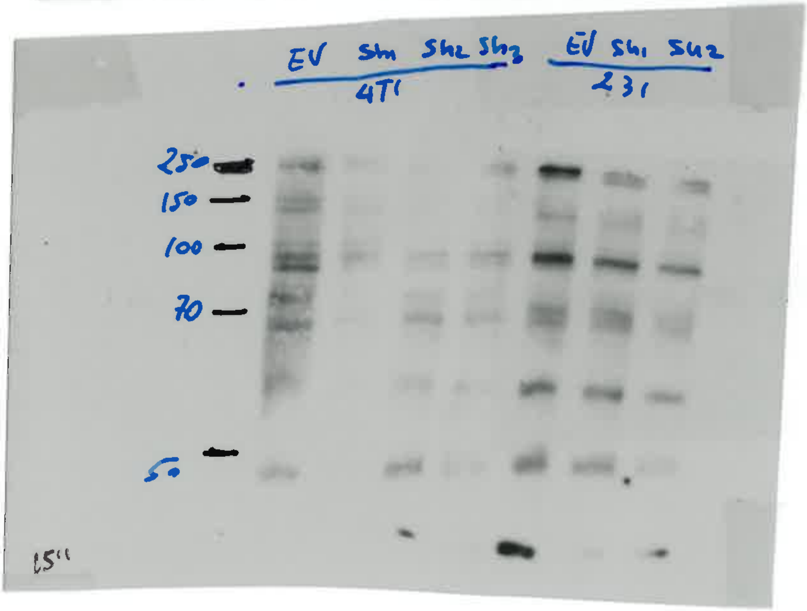

* CH Δ8 1:000
BSA 5%
Ab II 1,5 µl.

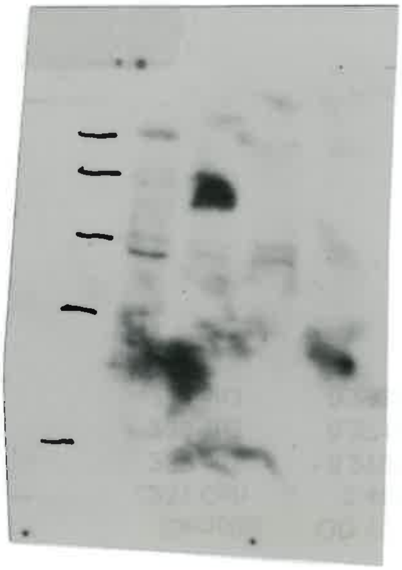

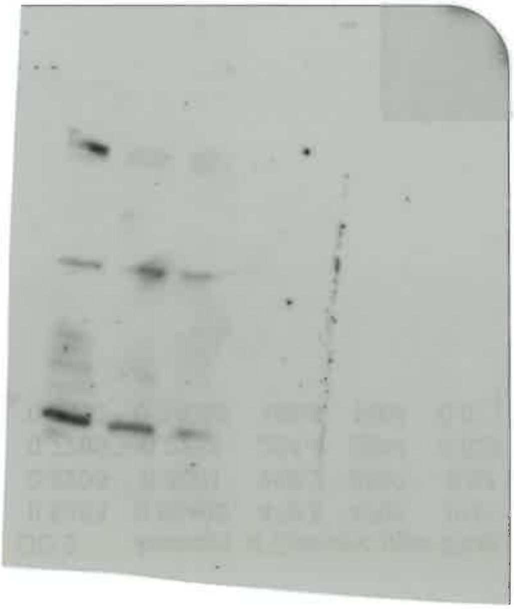

* CH Δ8 1:1000
Milk 5%
AbII 1,5 µl.
