# Supplementary material for: CHD8 interacts with BCL11A to induce oncogenic transcription in triple negative breast cancer
Source: EMBO J. 2025 May 6;44(12):3448–67. doi: 10.1038/s44318-025-00447-8 (PMC12170886; doi:10.1038/s44318-025-00447-8)
Supplement: Supplementary file 34 — Appendix Figure Source Data [file 44318_2025_447_MOESM34_ESM.zip › Appendix_Figure_S8.zip!/Appendix Figure S11/CHD8_19-9-15.pdf]

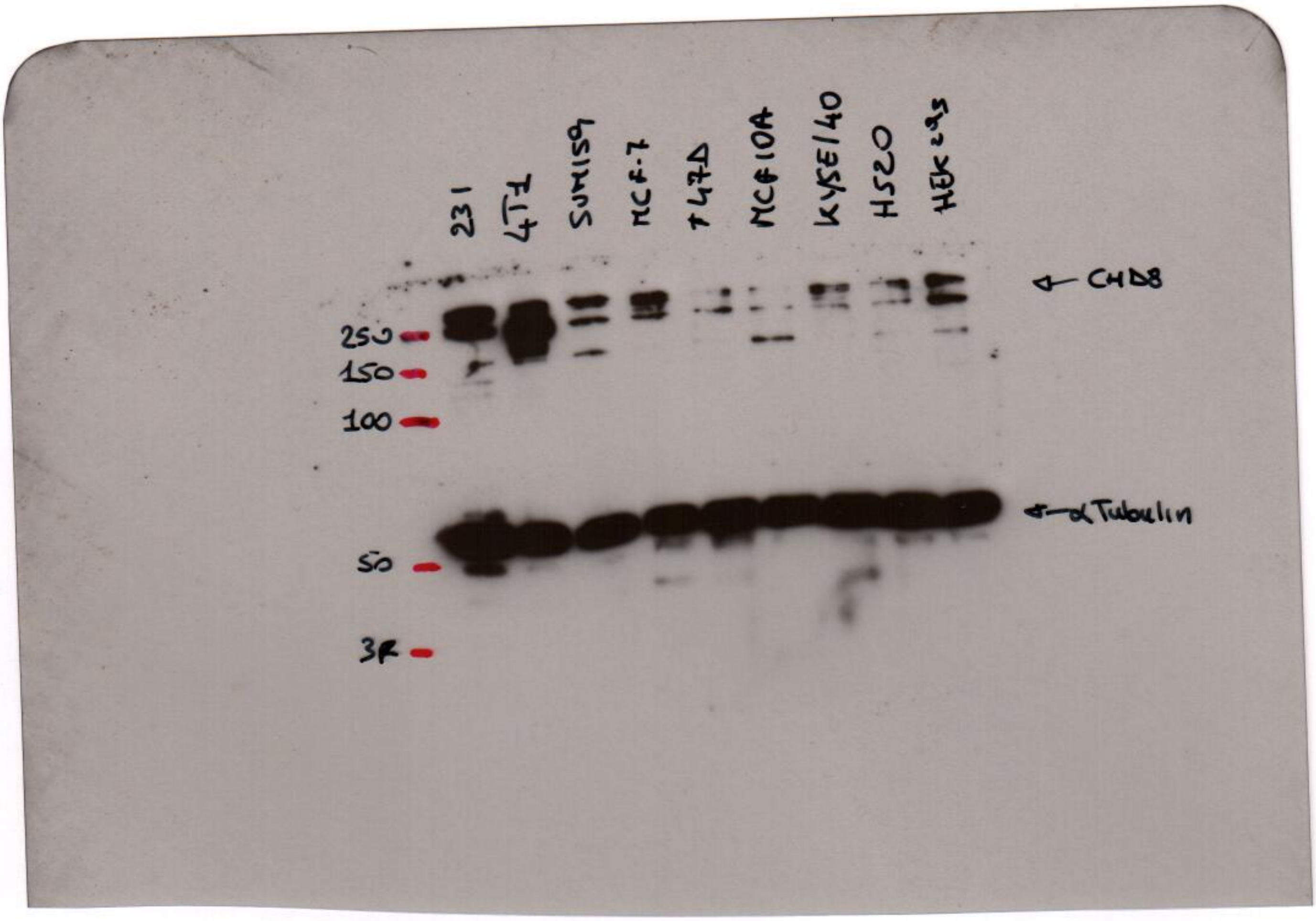
231
4T1
SUM159
MCF-7
T47D
MCF10A
KYSE140
H520
HEK293
CHD8
αTubulin
250
150
100
50
37
